# Synthesis of 3,4-Bis(Butylselanyl)Selenophenes and 4-Alkoxyselenophenes Promoted by Oxone^®^

**DOI:** 10.3390/molecules26082378

**Published:** 2021-04-19

**Authors:** Paola S. Hellwig, Jonatan S. Guedes, Angelita M. Barcellos, Gelson Perin, Eder J. Lenardão

**Affiliations:** LASOL—CCQFA, Universidade Federal de Pelotas—UFPel, P.O. Box 354, Pelotas 96010-900, RS, Brazil; psh.1996@hotmail.com (P.S.H.); vargsjon@gmail.com (J.S.G.); angelita.barcellos@gmail.com (A.M.B.)

**Keywords:** 1,3-diynes, electrophilic cyclization, heterocycle, organoselenium, selenophene

## Abstract

We describe herein an alternative transition-metal-free procedure to access 3,4-bis(butylselanyl)selenophenes and the so far unprecedented 3-(butylselanyl)-4-alkoxyselenophenes. The protocol involves the 5-*endo*-dig electrophilic cyclization of 1,3-diynes promoted by electrophilic organoselenium species, generated in situ through the oxidative cleavage of the Se-Se bond of dibutyl diselenide using Oxone^®^ as a green oxidant. The selective formation of the title products was achieved by controlling the solvent identity and the amount of dibutyl diselenide. By using 4.0 equiv of dibutyl diselenide and acetonitrile as solvent at 80 °C, four examples of 3,4-bis(butylselanyl)selenophenes were obtained in moderate to good yields (40–78%). When 3.0 equiv of dibutyl diselenide were used, in the presence of aliphatic alcohols as solvent/nucleophiles under reflux, 10 3-(butylselanyl)-4-alkoxyselenophenes were selectively obtained in low to good yields (15–80%).

## 1. Introduction

Selenophenes and their derivatives represent an important class of heterocyclic compounds. They have been extensively studied due to their intrinsic biological activities, e.g., antibacterial [1], anticonvulsant [2,3], antidepressant [4,5,6,7,8,9], antioxidant [10], antitumoral [11,12,13,14,15], hepatoprotective [16,17], and antinociceptive [18], among others [19]. In the field of materials science, they have interesting characteristics, such as organic light-emitting diodes (OLEDs) [20,21,22,23], organic field-effect transistors (OFETs) [24,25,26], organic solar cells (OSC) [27,28,29,30,31], and thin-film transistors [32,33,34]. In addition, selenophenes pre-activated with a halide or an organometallic (Li, Mg, Sn or Zn) can be used as building blocks in the formation of new C-C [35,36,37,38,39,40,41], C-N [42,43,44] and C-S [45] bonds under metal-catalyzed conditions. Alternatively, inactivated selenophenes have been used as reagents in several synthetic transformations, through palladium-catalyzed direct C-H bond activation [46,47,48,49,50].

**Scheme 1:**
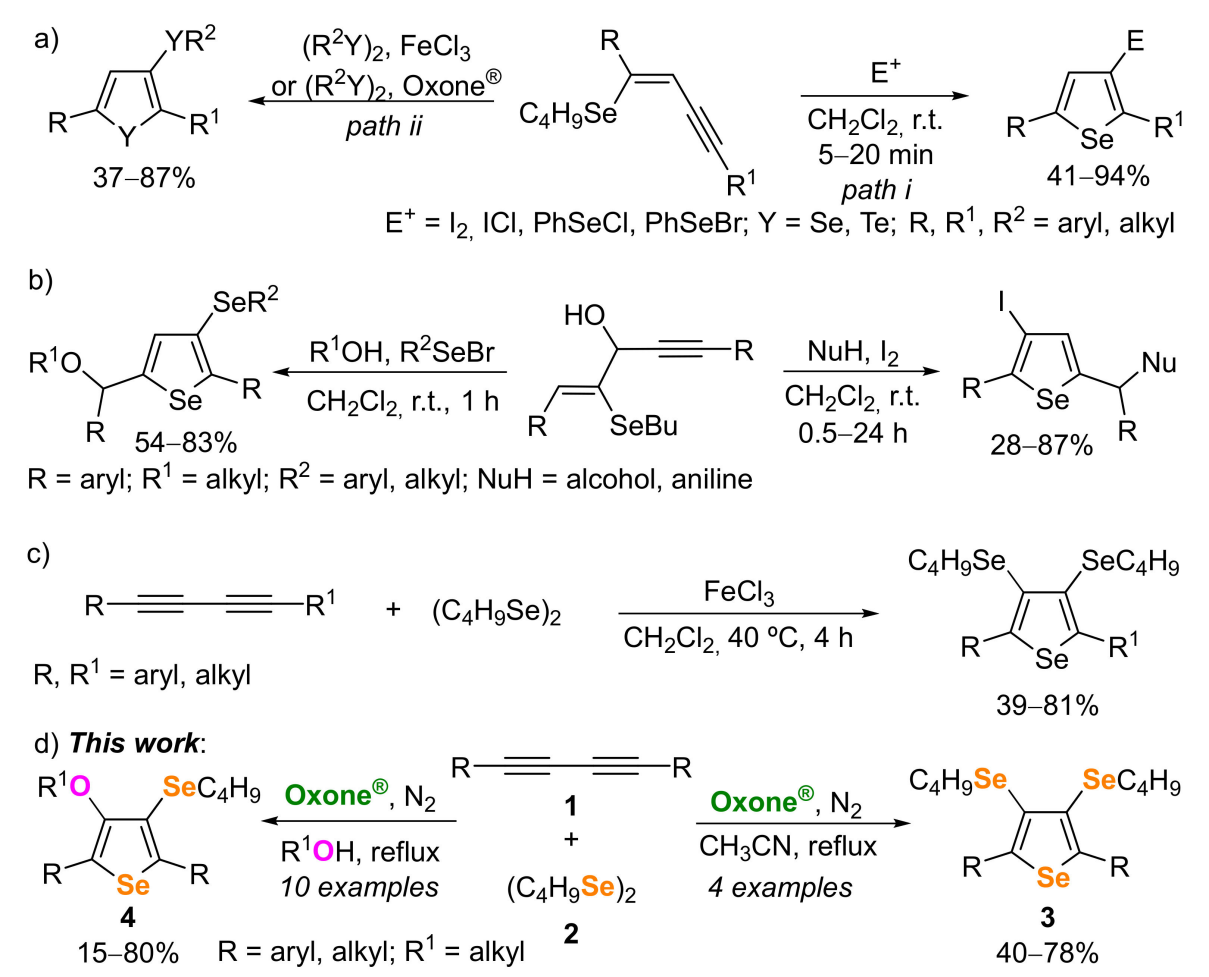
Previously reported methods for the synthesis of selenophenes and derivatives (**a**–**c**) and our general protocol for the synthesis of selenophenes promoted by Oxone^®^ (**d**).

Considering the growing potential utility of selenophenes in pharmaceutical, materials science, and organic synthesis, different methodologies have been reported for the preparation of this class of compounds [51]. Among these protocols, a general approach for the synthesis of 3-substituted selenophenes is the electrophilic cyclization of (*Z*)-selenoenynes with different electrophiles, such as I_2_, ICl, PhSeBr, and PhSeCl (Scheme 1a, path i) [52] or with electrophilic selenium species generated in situ from diorganyl dichalcogenides in the presence of FeCl_3_ [5] or Oxone^®^ [53] (Scheme 1a, path ii). In addition, in 2017 the electrophilic cyclization of selenoenynes in the presence of an appropriate nucleophile, affording 3-iodo-selenophenes and 3-organoselanyl-selenophenes (Scheme 1b), was reported [54]. The Bu_2_Se_2_/FeCl_3_ combination was also used in the cyclization of 1,3-diynes for the synthesis of 3,4-bis(butylselanyl)selenophenes (Scheme 1c) [55]. More recently this year, a three-component approach involving dialkyl acetylenedicarboxylate, ethyl 2-cyano-3-arylacrylates, and KSeCN was described to access functionalized selenophenes [56].

Thus, in view of the ample applicability of the selenophene core, and in continuation to our studies on the development of green protocols to prepare organochalcogen compounds, we described here a new and transition-metal-free protocol for the synthesis of selenophenes functionalized with organochalcogen groups. This protocol involves the 5-*endo*-dig electrophilic cyclization of 1,3-diynes **1** promoted by Oxone^®^ as a green oxidizing agent [57,58,59,60,61] and dibutyl diselenide **2** to prepare 3,4-bis(butylselanyl)selenophenes **3** and 3-butylselanyl-4-alkoxyselenophenes **4** (Scheme 1d).

## 2. Results and Discussion

To start our studies, 1,4-diphenylbuta-1,3-diyne **1a** and dibutyl diselenide **2a** were chosen as model substrates in the reaction with Oxone^®^. The reactions were monitored by TLC until total disappearance of the 1,3-diyne **1a**. Firstly, a mixture of **1a** (0.25 mmol), **2a** (0.50 mmol), and Oxone^®^ (0.50 mmol) in acetonitrile (3.0 mL) was stirred at 80 °C for 72 h in a conventional system under nitrogen atmosphere, affording the 3,4-bis(butylselanyl)-2,5-diphenylselenophene **3a** in 58% yield (Table 1, entry 1). Interested in reducing the reaction time and increasing the yield of the product **3a**, we decided to perform a reaction under ultrasonic irradiation (US, 20 kHz, 60% of amplitude) and, unfortunately, the expected product **3a** was obtained in only 15% yield after 2 h (Table 1, entry 2). Thus, studies using this energy were abandoned.

Based on this, the conventional heating system (oil bath) was utilized in the following experiments to verify the influence of different solvents in the reaction (Table 1, entries 3–6). In the reactions using dimethylformamide, PEG-400, and glycerol as solvents, the desired product **3a** was not obtained, as observed by GC/MS analysis, and the starting materials were recovered (Table 1, entries 3–5). Surprisingly, when ethanol was used as a solvent, after 24 h we observed the total consumption of 1,3-diyne **1a** by TLC, and the expected product **3a** was obtained in only 15% yield, combined with 43% yield of 3-(butylselanyl)-4-ethoxy-2,5-diphenylselenophene **4a** (Table 1, entry 6). Focused on selectively preparing selenophene **3a**, acetonitrile was set as the best solvent. Subsequently, the effect of using different quantities of Oxone^®^ was evaluated (Table 1, entries 7–10). When the amount of Oxone^®^ was increased to 0.75 mmol, product **3a** was obtained in 78% yield after 48 h of reaction (Table 1, entry 7). Lower yields were obtained with larger (1.0 mmol) or smaller amounts (0.25 mmol or 0.38 mmol) of Oxone^®^, affording the desired compound **3a** in 75%, 38%, and 50% yield, respectively (Table 1, entries 8–10).

In view of the interesting result obtained using ethanol as a solvent (Table 1, line 6), we decided to optimize the reaction conditions aiming to maximize the formation of product **4a**. Thus, when the amount of dibutyl diselenide **2a** was reduced to 0.38 mmol (3.0 equiv), the respective compound **4a** was obtained in 48% yield after 24 h of reaction (Table 1, entry 11). In addition, when the reaction was carried out using 0.25 mmol (2.0 equiv) of **2a**, the desired product **4a** was obtained in only 35% yield after 36 h, with incomplete consumption of 1,3-diyne **1a** (Table 1, entry 12). Thus, the amount of dibutyl diselenide **2a** was fixed at 0.38 mmol and different amounts of Oxone^®^ were then evaluated (Table 1, entries 13–16). The results indicated that a decrease in the amount of the oxidizing agent (to 0.25 and 0.38 mmol) caused a decrease in the reaction efficiency, affording compound **4a** in 39% and 42% yield after 48 h and 36 h, respectively (Table 1, entries 13 and 14). A better result was obtained using 0.75 mmol of Oxone^®^ (70% yield after 24 h), while, when 1.0 mmol of the Oxone^®^ was used, the yield of **4a** dropped to 65% (Table 1, entries 15 and 16). Thus, 0.75 mmol of Oxone^®^ was established as the ideal amount of oxidizing agent in the cyclization reaction. Finally, the reaction was carried out under air atmosphere (open flask), affording compound **4a** in only 15% yield after 48 h (Table 1, entry 17). Based on the results depicted in Table 1, the optimal condition to prepare 3,4-bis(butylselanyl)-2,5-diphenylselenophene **3a** was that of entry 7, which involved a mixture of 0.25 mmol of 1,4-diphenylbuta-1,3-diyne **1a**, 0.50 mmol (4.0 equiv) of dibutyl diselenide **2a**, and 0.75 mmol of Oxone^®^ in acetonitrile as the solvent (3.0 mL) at 80 °C for 48 h under nitrogen atmosphere (Table 1, entry 7). On the other hand, 3-(butylselanyl)-4-ethoxy-2,5-diphenylselenophene **4a** was selectively prepared using the conditions described in Table 1, entry 15, in which a mixture of the 1,3-diyne **1a** (0.25 mmol) was reacted with dibutyl diselenide **2a** (0.38 mmol, 3.0 equiv) and Oxone^®^ (0.75 mmol) in ethanol as solvent (3.0 mL) at reflux temperature under nitrogen atmosphere for 24 h.

Once the best conditions were determined for the synthesis of 3,4-bis(butylselanyl)-2,5-diphenylselenophene **3a**, the scope and limitations of the methodology were explored by reacting different 1,3-diynes **1b**–**f** with dibutyl diselenide **2a**, and the results are shown in Table 2. Firstly, the effect of electron donating groups (EDGs) and electron withdrawing groups (EWGs) bonded in the aromatic rings of 1,3-diyne **1** was examined in the reaction with **2a**. Thus, when the electron-rich 1,3-diynes **1b** (R = 4-CH_3_OC_6_H_4_) and **1c** (R = 4-CH_3_C_6_H_4_) were used, the corresponding products, **3b** and **3c**, were obtained in 50% and 70% yield after 2.5 h and 4 h of reaction, respectively (Table 2, compounds **3b** and **3c**). However, the presence of the EWG chlorine in the *para*-position of the phenyl ring negatively affected the reaction, and compounds **3d** (R = 4-ClC_6_H_4_) could not be obtained, even after refluxing for 72 h, as indicated by GC/MS analysis. The starting materials, **1d** and **2a**, were recovered (Table 2, compound **3d**).

In addition, when the reaction was carried out using 1,4-di(naphthalen-2-yl)buta-1,3-diyne **1e**, the respective selenophene **3e** was obtained in 40% yield, after 48 h (Table 2, compound **3e**). In contrast, despite the complete consumption of the starting materials, the reaction using dodeca-5,7-diyne **1f** gave a complex mixture of decomposition products after 5 h of reaction, instead of the expected alkyl-substituted selenophene **3f** (Table 2, compound **3f**). Finally, we evaluated the reaction of 1,4-diphenylbuta-1,3-diyne **1a** with other dialkyl dichalcogenides **2b** (Y = Te) and **2c** (Y = S). Unfortunately, in both cases the desired products, **3g** and **3h**, were not obtained, even after 72 h of reaction, as indicated by GC/MS analysis, and the starting materials were recovered (Table 2, compounds **3g** and **3h**).

Next, the versatility and limitations of this protocol for accessing the new 4-alkoxyselenophenes **4** was evaluated by reacting several 1,3-diynes **1a**–**j** with dialkyl dichalcogenide **2** in the presence of different solvents/nucleophiles (Table 3). In general, we observed that the reactivity was affected both by electronic and steric effects in the 1,3-diynes and in the solvent. 1,4-diphenylbuta-1,3-diyne **1a** reacted with dibutyl diselenide **2a** in methanol as the solvent, affording the respective 4-methoxyselenophene **4b** in 75% yield after 24 h (Table 3, compound **4b**). When the secondary alcohol *iso*-propanol was used instead of methanol, the alkoxy derivative **4c** was obtained in only 35% yield, while the products’ derivative of *tert*-butanol **4d** and phenol **4e** were not observed under the optimal conditions, even after 72 h of reaction, as indicated by GC/MS analysis (Table 3, compounds **4c**–**e**). The lower reactivity of *iso*-propanol and *tert*-butanol can be explained by steric effects, while phenol was not sufficiently nucleophilic to form the reactive intermediate in the reaction (see below a plausible mechanism). Next, the same reaction was performed in the presence of thiols and amines, aiming to verify the possibility of preparing thio- and amino-substituted selenophenes through the cyclization of 1,3-diyne **1a**. Unfortunately, the limitation of this protocol was observed when aryl and alkyl thiols and amines (2 equiv) were used in the presence of acetonitrile as solvent (3.0 mL). In these cases, the starting materials were not consumed, even after 48 h of reaction, as indicated by GC/MS analysis.

In the sequence, we investigated the reactivity of several symmetrical 1,3-diynes **1** with dibutyl diselenide **2a** in the presence of ethanol or methanol. Similar to what we observed in the synthesis of selenophenes **3** (Table 2), the presence of EDGs and EWGs at the *para*-position of the pendant phenyl ring of the diyne remarkably influenced the reactivity. Accordingly, selenophenes **4f** (R = 4-CH_3_OC_6_H_4_, R^1^ = C_2_H_5_), **4g** (R = 4-CH_3_OC_6_H_4_, R^1^ = CH_3_), and **4h** (R = 4-CH_3_C_6_H_4_, R^1^ = C_2_H_5_) were obtained in 35%, 40%, and 80% yield, respectively, while **4i** (R = 4-ClC_6_H_4_, R^1^ = C_2_H_5_) was not observed (Table 3, compounds **4f**–**i**). The lack of reactivity of 1,4-bis(4-chlorophenyl)buta-1,3-diyne **1d** was probably due to the low stability of the intermediate involved in the cyclization to form the 4-alcoxyselenophene **4i**. The structure of compound **4h** was confirmed by an additional NMR analysis, which is available in the SI (Figures S25–S27). The presence of the 2-naphthyl groups in diyne **1e** negatively influenced the reaction, and the respective product, **4j**, did not form, even after 48 h, presumably due to the steric congestion around the triple bonds (Table 3, compound **4j**). Additionally, we investigated the reactivity of the alkyl-substituted dodeca-5,7-diyne **1f** and of the propargyl alcohol derivative **1g**. In these cases, despite the starting materials being totally consumed, the corresponding selenophenes, **4k** and **4l**, were not observed, and a complex mixture of compounds was formed (Table 3, compounds **4k** and **4l**). In contrast, when sterically hindered *ortho*-substituted 1,4-diaryl diynes were used, a similar reactivity was observed when EDG (**1h**, R = 2-CH_3_C_6_H_4_) or EWG (**1i**, R = 2-ClC_6_H_4_) groups were present, and the respective 4-ethoxyselenophenes, **4m** and **4o**, were obtained, both in 15% yield after 72 h of reaction (Table 3, compounds **4m** and **4o**). When methanol was used instead of ethanol, the 4-methoxyselenophenes **4n** and **4p** were obtained, both in 25% yield after 60 h (Table 3, compounds **4n** and **4p**). As observed in the reactions in CH_3_CN, dibutyl ditelluride **2b** and dimethyl disulfide **2c** were not suitable substrates in the reaction. After 48 h of refluxing in ethanol, no products were observed and the starting materials were recovered (Table 3, compounds **4q** and **4r**).

**Scheme 2:**
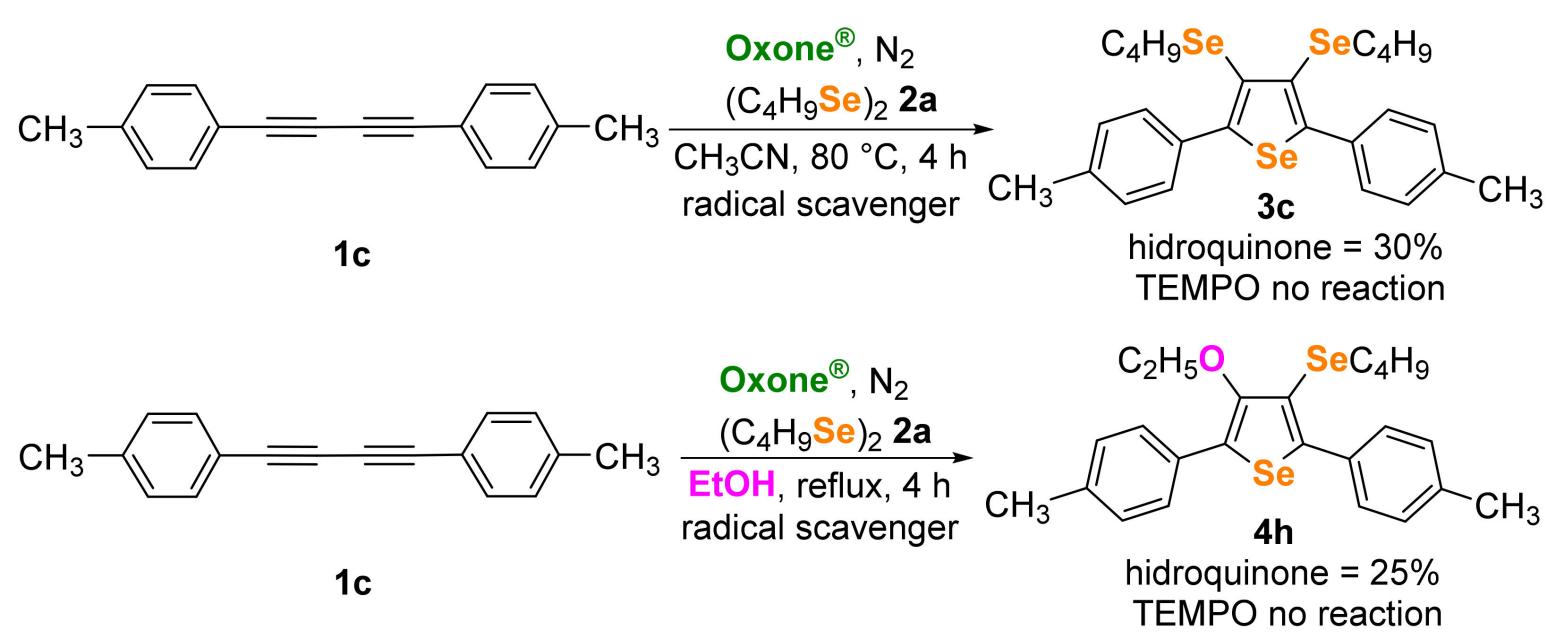
Reactions in the presence of radical scavenger hydroquinone and TEMPO.

In order to collect data to elucidate the mechanism of the synthesis of 3,4-bis(butylselanyl)selenophenes **3** and 3-(butylselanyl)-4-alkoxy-selenophenes **4**, some control experiments were conducted (Scheme 2). Thus, the reaction between 1,4-bis-4-tolylbuta-1,3-diyne **1c** and dibutyl diselenide **2a** was conducted in the presence of 3.0 equiv of the radical scavenger benzene-1,4-diol (hydroquinone) and 2,2,6,6-tetramethylpiperidin-1-oxyl (TEMPO). In these experiments, after 4 h of reaction the products, **3c** and **4h,** were obtained in 30% and 25% yield, respectively, when hydroquinone was used. In contrast, the formation of the products **3c** and **4h** was not observed in the presence of TEMPO. Considering that products **3c** and **4h** were formed in the presence of hydroquinone, even in lower yields, the reaction could have occurred via ionic and radical pathways. Thus, based on our knowledge and the literature [57,58,59], we believe that the step of formation of the activation can occur by both radical and ionic mechanisms, whereas the cyclization step proceeds via ionic pathway.

**Scheme 3:**
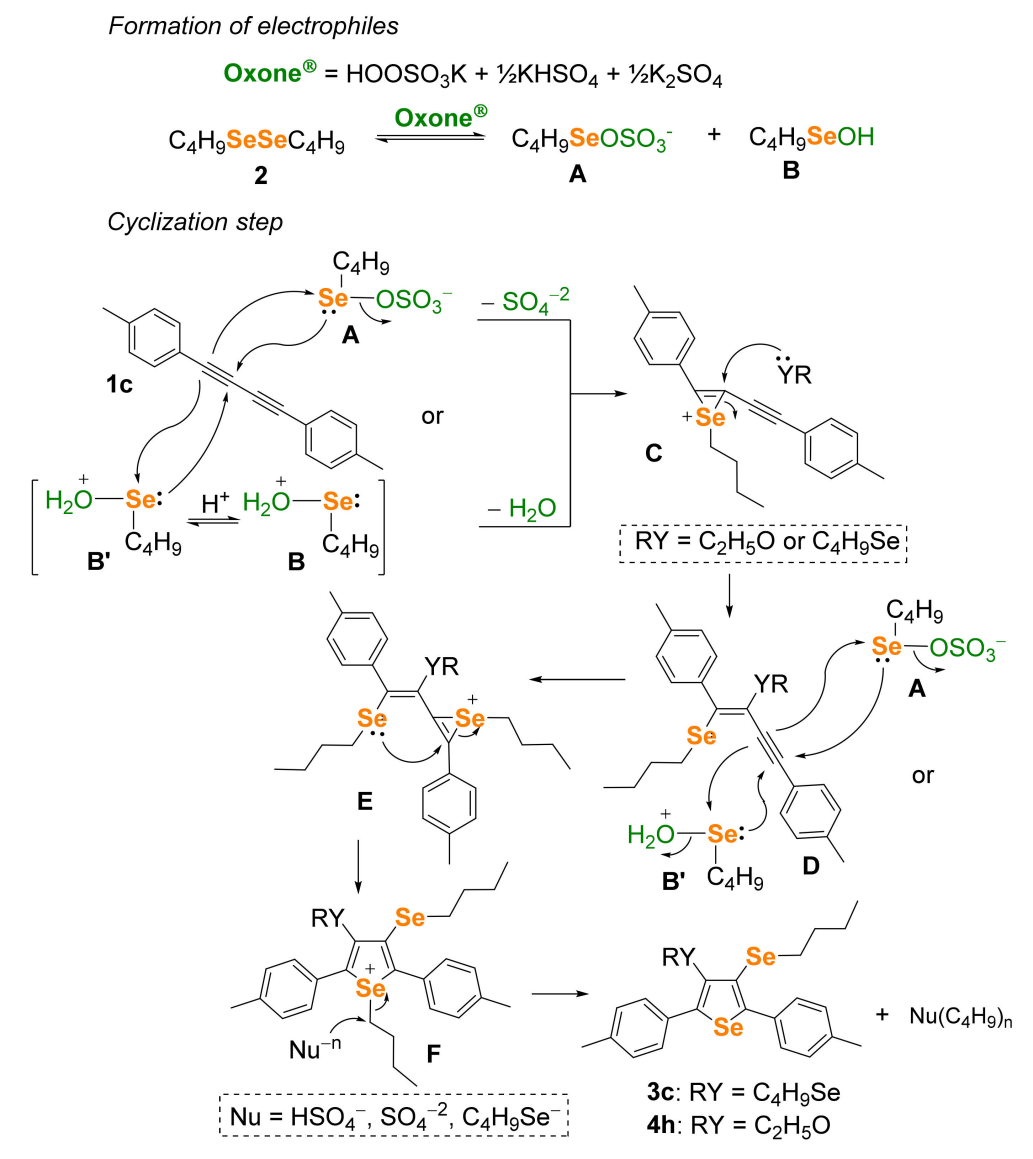
Plausible mechanism for the synthesis of **3c** and **4h**.

Thus, based on our own results and in the literature on the reactivity of Oxone^®^ [57,58,59,60,61] and electrophilic cyclization reactions [53,54,55], a plausible mechanism for the formation of 3,4-bis(butylselanyl)selenophene **3c** and 3-(butylselanyl)-4-ethoxy-2,5-di-4-tolylselenophene **4h**, through the reaction of 1,4-bis-4-tolylbuta-1,3-diyne **1c** with dibutyl diselenide **2a** promoted by Oxone^®^, is presented in Scheme 3. The first step for the synthesis of **3c** and **4h** consisted of the formation of the electrophilic selenium species, **A** and **B**, via ionic or radical pathways, from the reaction between dibutyl diselenide **2a** and potassium peroxymonosulfate (KHSO_5_), the active component of Oxone^®^ [57,58,59]. In the cyclization step, diyne **1c** reacted with **A** or **B’**, affording seleniranium intermediate **C**, and releasing hydrogen sulfate anion (HSO_4_^−^) and water (H_2_O) to the medium. This was the common intermediate in both reactions, to prepare **3c** or **4h**. In the sequence, a nucleophilic attack by RY (BuSe- or EtOH) occurred in the double bond of seleniranium intermediate **C**, producing the respective enyne intermediate **D**. After, the interaction of the electrophilic species **A** or **B’** with the C-C triple bond of intermediate **D** occurred, affording the seleniranium intermediate **E** (Scheme 3). In the sequence, an intramolecular nucleophilic attack of the selenium atom to seleniranium intermediate **E** afforded the cationic selenophene **F**, via a 5-*endo*-dig electrophilic cyclization process. In the last step, the displacement of the butyl group from intermediate **F** by a nucleophile (Nu = HSO_4_^−^, SO_4_^2−^ or C_4_H_9_Se^−^) afforded the desired 3,4-bis(butylselanyl)selenophene **3c** or 4-ethoxyselenophene **4h**, respectively.

## 3. Materials and Methods

The reactions were monitored by TLC sheets ALUGRAM^®^ Xtra SIL G/UV_254_. For visualization, TLC plates were either placed under UV light or stained with iodine vapor and 5% vanillin in 10% H_2_SO_4_ and heat. Preparative layer with UV_254_ (20 × 20 cm—500 microns) was used in the chromatographic purification of compounds **3** and **4**. Hydrogen nuclear magnetic resonance spectra (^1^H NMR) were obtained on Bruker Avance III HD 400 MHz employing a direct broadband probe at 400 MHz. The spectra were recorded in CDCl_3_ solutions. The chemical shifts are reported in ppm, referenced to tetramethylsilane (TMS) as the internal reference. Coupling constants (*J*) are reported in Hertz. Abbreviations to denote the multiplicity of a particular signal are s (singlet), d (doublet), dd (doublet of doublet), t (triplet), quint (quintet), sext (sextet), sept (septet), and m (multiplet). Carbon-13 nuclear magnetic resonance spectra (^13^C NMR) were obtained on Bruker Avance III HD 400 MHz employing a direct broadband probe at 100 MHz. The chemical shifts are reported in ppm, referenced to the solvent peak of CDCl_3_ (*δ* 77.0 ppm). Selenium-77 nuclear magnetic resonance (^77^Se NMR) spectra were obtained at 76 MHz, using (PhSe)_2_ as an internal standard. Low-resolution mass spectra (MS) were measured on a Shimadzu GC-MS-QP2010 mass spectrometer. The high-resolution atmospheric pressure chemical ionization (APCI-QTOF) analyses were performed on a Bruker Daltonics micrOTOF-Q II instrument operating in the positive ion detection mode. For data acquisition and processing, Compass 1.3 for micrOTOF-Q II software (Bruker Daltonics, Billerica, MA, USA) was used. Melting point (m.p.) values were measured in a Marte PFD III instrument with a 0.1 °C precision. Oxone^®^ was purchased from Sigma-Aldrich. The 1,3-diynes **1** were prepared as described in the Supplementary Materials. To work safely with selenium compounds, these must be handled carefully in a chemical fume hood, and gloves and goggles should be worn. No other special lab practice is required.

### 3.1. General Procedure for the Synthesis of 3,4-Bis(Butylselanyl)Selenophenes **3**

To a 25.0-mL, two-necked, round-bottomed flask equipped with magnetic stirring and a reflux system containing the appropriate 1,3-diyne **1a**–**f** (0.25 mmol), a solution of dibutyl diselenide **2a** (0.50 mmol, 0.14 g) in acetonitrile (3.0 mL) and Oxone^®^ (KHSO_5_.^1^/_2_KHSO_4_.^1^/_2_K_2_SO_4_, MM = 307 g·mol^−1^, 0.75 mmol, 0.23 g) were added under nitrogen atmosphere. The resulting mixture was stirred at reflux temperature for the time indicated in Table 2. The reactions were monitored by TLC until total disappearance of the 1,3-diyne 1. After this time, the resulting solution was received in water (10.0 mL) and the product was extracted with ethyl acetate (3 × 10.0 mL). The organic layer was separated, dried with MgSO_4_, and concentrated under vacuum. The desired product was isolated by preparative thin-layer chromatography using hexane as eluent. Yield: 40–78%.

**3,4-Bis(butylselanyl)-2,5-diphenylselenophene 3a**: [55] Yield: 0.108 g (78%); yellow oil. ^1^H NMR (CDCl_3_, 400 MHz) *δ* (ppm) = 7.52–7.50 (m, 4H); 7.34–7.25 (m, 6H); 2.56 (t, *J* = 7.4 Hz, 4H); 1.36 (quint, *J* = 7.4 Hz, 4H); 1.12 (sext, *J* = 7.4 Hz, 4H); 0.69 (t, *J* = 7.4 Hz, 6H). ^13^C NMR (CDCl_3_, 100 MHz) *δ* (ppm) = 149.89, 136.87, 129.77, 128.98, 128.11, 127.91, 31.83, 29.63, 22.70, 13.49. ^77^Se NMR (CDCl_3_, 76 MHz) *δ* (ppm) = 716.0, 233.8. MS (rel. int., %) *m*/*z*: 556 (M^+^, 23.6), 362 (45.8), 262 (100.0), 202 (98.4), 57 (21.8).

**3,4-Bis(butylselanyl)-2,5-bis(4-methoxyphenyl)selenophene 3b**: [55] Yield: 0.077 g (50%); yellow oil. ^1^H NMR (CDCl_3_, 400 MHz) *δ (*ppm) = 7.51 (d, *J* = 8.6 Hz, 4H); 6.93 (d, *J* = 8.6 Hz, 4H); 3.85 (s, 6H); 2.64 (t, *J* = 7.4 Hz, 4H); 1.45 (quint, *J* = 7.4 Hz, 4H); 1.22 (sext, *J* = 7.4 Hz, 4H); 0.78 (t, *J* = 7.4 Hz, 6H). ^13^C NMR (CDCl_3_, 100 MHz) *δ (*ppm) = 159.35, 149.33, 130.94, 129.43, 128.41, 113.52, 55.31, 31.88, 29.61, 22.76, 13.53. ^77^Se NMR (CDCl_3_, 76 MHz) *δ* (ppm) = 709.0, 231.6. MS (rel. int., %) *m*/*z*: 616 (M^+^, 1.17), 343 (6.0), 281 (13.1), 207 (41.3), 73 (20.6), 44 (100.0).

**3,4-Bis(butylselanyl)-2,5-di-4-tolylselenophene 3c**: [55] Yield: 0.102 g (70%); yellow oil. ^1^H NMR (CDCl_3_, 400 MHz) *δ (*ppm) = 7.47 (d, *J* = 8.0 Hz, 4H); 7.20 (d, *J* = 8.0 Hz, 4H); 2.65 (t, *J* = 7.4 Hz, 4H); 2.39 (s, 6H); 1.45 (quint, *J* = 7.4 Hz, 4H); 1.26–1.17 (m, 4H); 0.78 (t, *J* = 7.4 Hz, 6H). ^13^C NMR (CDCl_3_, 100 MHz) *δ (*ppm) = 149.91, 137.80, 134.06, 129.61, 128.82, 128.60, 31.88, 29.66, 22.76, 21.29, 13.52. ^77^Se NMR (CDCl_3_, 76 MHz) *δ* (ppm) = 712.9, 232.4. MS (rel. int., %) *m*/*z*: 584 (M^+^, 23.0), 389 (35.6), 310 (100.0), 230 (46.0), 207 (56.0), 44 (65.9).

**3,4-Bis(butylselanyl)-2,5-di(naphthalene-2-yl)selenophene 3e**: [55] Yield: 0.0656 g (40%); light brown solid, m.p: 69–72 °C (Lit.[55]: 70–72 °C). ^1^H NMR (CDCl_3_, 400 MHz) *δ (*ppm) = 8.05 (s, 2H); 7.89–7.86 (m, 6H); 7.82–7.79 (m, 2H); 7.52–7.50 (m, 4H); 2.67 (t, *J* = 7.4 Hz, 4H); 1.46 (quint, *J* = 7.4 Hz, 4H); 1.18 (sext, *J* = 7.4 Hz, 4H); 0.72 (t, *J* = 7.4 Hz, 6H). ^13^C NMR (CDCl_3_, 100 MHz) *δ (*ppm) = 150.10, 134.35, 133.05, 132.80, 129.49, 128.78, 128.17, 127.72, 127.67, 127.64, 126.40, 31.91, 29.77, 22.71, 13.47. ^77^Se NMR (CDCl_3_, 76 MHz) *δ* (ppm) = 718.3, 235.6. MS (rel. int., %) *m*/*z*: 656 (M^+^, 2.35), 459 (4.0), 381 (13.0), 207 (100.0), 73 (55.7).

### 3.2. General Procedure for the Synthesis of 3-(Butylselanyl)-4-Alkoxyselenophenes **4**

To a 25.0-mL, two-necked, round-bottomed flask equipped with magnetic stirring and a reflux system containing the appropriate 1,3-diyne **1a**–**i** (0.25 mmol), a solution of dibutyl diselenide **2a** (0.38 mmol, 0.10 g) in the alcohol corresponding (3.0 mL) and Oxone^®^ (KHSO_5_.^1^/_2_KHSO_4_.^1^/_2_K_2_SO_4_, MM = 307 g.mol^−1^, 0.75 mmol, 0.23 g) were added under nitrogen atmosphere. The resulting mixture was stirred at reflux temperature for the time indicated in Table 3. The reactions were monitored by TLC until total disappearance of the 1,3-diyne 1. After this time, the resulting solution was received in water (10.0 mL) and the product was extracted with ethyl acetate (3 × 10.0 mL). The organic layer was separated, dried with MgSO_4_, and concentrated under vacuum. The desired product was isolated by preparative thin-layer chromatography using hexane as eluent. Yield: 15–80%.

**3-(Butylselanyl)-4-ethoxy-2,5-diphenylselenophene 4a**: Yield: 0.081 g (70%); yellow oil. ^1^H NMR (CDCl_3_, 400 MHz) *δ* (ppm) = 7.64 (d, *J* = 7.6 Hz, 2H); 7.50 (d, *J* = 7.2 Hz, 2H); 7.34–7.24 (m, 5H); 7.20–7.16 (m, 1H); 3.91 (q, *J* = 7.0 Hz, 2H); 2.69 (t, *J* = 7.4 Hz, 2H); 1.42 (quint, *J* = 7.4 Hz, 2H); 1.25 (t, *J* = 7.0 Hz, 3H); 1.21–1.13 (m, 2H); 0.72 (t, *J* = 7.4 Hz, 3H). ^13^C NMR (CDCl_3_, 100 MHz) *δ* (ppm) = 154.08, 145.39, 136.82, 134.64, 132.11, 129.42, 128.64, 128.17, 127.94, 127.83, 127.19, 120.01, 69.19, 32.13, 27.89, 22.65, 15.71, 13.49. MS (rel. int., %) *m*/*z*: 464 (M^+^, 73.8), 408 (22.9), 327 (10.0), 298 (18.3), 218 (40.2), 191 (37.8), 169 (100.0). HRMS (APCI-QTOF) calculated mass for C_22_H_24_OSe_2_ [M]^+^: 464.0156, found: 464.0181.

**3-(Butylselanyl)-4-methoxy-2,5-diphenylselenophene 4b**: Yield: 0.084 g (75%); yellow oil. ^1^H NMR (CDCl_3_, 400 MHz) *δ* (ppm) = 7.72–7.70 (m, 2H); 7.59–7.56 (m, 2H); 7.42–7.35 (m, 5H); 7.30–7.24 (m, 1H); 3.79 (s, 3H); 2.74 (t, *J* = 7.4 Hz, 2H); 1.50 (quint, *J* = 7.4 Hz, 2H); 1.25 (sext, *J* = 7.4 Hz, 2H); 0.79 (t, *J* = 7.4 Hz, 3H). ^13^C NMR (CDCl_3_, 100 MHz) *δ (*ppm) = 154.93, 145.66, 136.73, 134.39, 131.76, 129.43, 128.73, 128.20, 128.00, 127.84, 127.31, 119.57, 60.77, 32.15, 27.98, 22.65, 13.49. ^77^Se NMR (CDCl_3_, 76 MHz) *δ* (ppm) = 582.0, 195.1. MS (rel. int., %) *m*/*z*: 450 (M^+^, 100.0), 394 (46.6), 312 (42.3), 202 (51.2), 169 (90.1), 44 (58.9). HRMS (APCI-QTOF) calculated mass for C_21_H_22_OSe_2_ [M]^+^: 449.9999, found: 450.0034.

**3-(Butylselanyl)-4-isopropoxy-2,5-diphenylselenophene 4c**: Yield: 0.042 g (35%); yellow oil. ^1^H NMR (CDCl_3_, 400 MHz) *δ* (ppm) = 7.64–7.61 (m, 2H); 7.52–7.49 (m, 2H); 7.35–7.27 (m, 5H); 7.20–7.17 (m, 1H); 4.35 (sept, *J* = 6.1 Hz, 1H); 2.67 (t, *J* = 7.4 Hz, 2H); 1.41 (quint, *J* = 7.4 Hz, 2H); 1.22–1.13 (m, 2H); 1.11 (d, *J* = 6,1 Hz, 6H); 0.72 (t, *J* = 7,4 Hz, 3H). ^13^C NMR (CDCl_3_, 100 MHz) *δ (*ppm) = 152.51, 145.28, 136.98, 135.16, 132.45, 129.45, 128.44, 128.34, 128.17, 127.90, 127.04, 120.69, 76.00, 32.05, 29.66, 27.85, 22.40, 13.50. ^77^Se NMR (CDCl_3_, 76 MHz) *δ* (ppm) = 578.8, 202.0. MS (rel. int., %) *m*/*z*: 478 (M^+^, 11.3), 436 (23.0), 300 (15.3), 207 (24.7), 169 (38.4), 44 (100.0). HRMS (APCI-QTOF) calculated mass for C_23_H_26_OSe_2_ [M]^+^: 478.0313, found: 478.0333.

**3-(Butylselanyl)-4-ethoxy-2,5-bis(4-methoxyphenyl)selenophene 4f**: Yield: 0.046 g (35%); yellow oil. ^1^H NMR (CDCl_3_, 400 MHz) *δ* (ppm) = 8.13 (d, *J* = 8.7 Hz, 1H); 7.64 (d, *J* = 8.7 Hz, 2H); 7.50 (d, *J* = 8.7 Hz, 2H); 6.95–6.90 (m, 3H); 3.97 (q, *J* = 7.0 Hz, 2H); 3.85 (s, 3H); 3.84 (s, 3H); 2.76 (t, *J* = 7.3 Hz, 2H); 1.60–1.47 (m, 4H); 1.32 (t, *J* = 7.0 Hz, 3H); 0.81 (t, *J* = 7.3 Hz, 3H). ^13^C NMR (CDCl_3_, 100 MHz) *δ* (ppm) = 159.38, 158.75, 153.19, 144.29, 130.61, 129.05, 128.08, 127.37, 123.68, 119.24, 114.02, 113.60, 68.97, 55.30 (2C), 32.17, 27.86, 22.70, 15.73, 13.53. MS (rel. int., %) *m*/*z*: 524 (M^+^, 2.3), 436 (23.0), 281 (10.3), 207 (22.9), 44 (100.0). HRMS (APCI-QTOF) calculated mass for C_24_H_28_O_3_Se_2_ [M]^+^: 524.0368, found: 524.0363.

**3-(Butylselanyl)-4-methoxy-2,5-bis(4-methoxyphenyl)selenophene 4g**: Yield: 0.051 g (40%); yellow oil. ^1^H NMR (CDCl_3_, 400 MHz) *δ* (ppm) = 7.63 (d, *J* = 8.6 Hz, 2H); 7.50 (d, *J* = 8.6 Hz, 2H); 6.97–6.91 (m, 4H); 3.85 (s, 3H); 3.84 (s, 3H); 3.76 (s, 3H); 2.74 (t, *J* = 7.4 Hz, 2H); 1.50 (quint, 7.4 Hz, 2H); 1.31–1.24 (m, 2H); 0.80 (t, *J* = 7.4 Hz, 3H). ^13^C NMR (CDCl_3_, 100 MHz) *δ* (ppm) = 159.42, 158.84, 154.04, 144.57, 130.94, 130.62, 129.35, 129.06, 127.09, 118.78, 114.11, 113.62, 60.60, 55.30 (2C), 32.17, 27.94, 22.69, 13.51. MS (rel. int., %) *m*/*z*: 510 (M^+^, 8.3), 207 (55.7), 73 (86.2), 44 (100.0). HRMS (APCI-QTOF) calculated mass for C_23_H_26_O_3_Se_2_ [M]^+^: 510.0212, found: 510.0220.

**3-(Butylselanyl)-4-ethoxy-2,5-di-4-tolylselenophene 4h**: Yield: 0.098 g (80%); yellow oil. ^1^H NMR (CDCl_3_, 400 MHz) *δ* (ppm) = 7.60 (d, *J* = 8.0 Hz, 2H); 7.46 (d, *J* = 8.0 Hz, 2H); 7.19 (t, *J* = 8.7 Hz, 4H); 3.98 (q, *J* = 7.0 Hz, 2H); 2.78 (t, *J* = 7.4 Hz, 2H); 2.38 (s, 3H); 2.36 (s, 3H); 1.51 (quint, *J* = 7.4 Hz, 2H); 1.33 (t, J = 7 Hz, 3H); 1.26 (sext, *J* = 7.4 Hz, 2H); 0.81 (t, *J* = 7.4 Hz, 3H). ^13^C NMR (CDCl_3_, 100 MHz) *δ* (ppm) = 153.70, 145.05, 137.82, 136.98, 134.04, 131.84, 129.32, 129.28, 128.89, 127.72, 127.68, 119.58, 69.03, 32.17, 27.87, 22.70, 21.27, 21.21, 15.74, 13.51. ^77^Se NMR (CDCl_3_, 76 MHz) *δ* (ppm) = 578.7, 198.5. MS (rel. int., %) *m*/*z*: 492 (M^+^, 89.4), 246 (62.0), 207 (48.2), 183 (100.0), 91 (27.7), 44 (27.9). HRMS (APCI-QTOF) calculated mass for C_24_H_29_OSe_2_ [M + H]^+^: 493.0548, found: 493.0539.

**3-(Butylselanyl)-4-ethoxy-2,5-di-2-tolylselenophene 4m**: Yield: 0.018 g (15%); yellow oil. ^1^H NMR (CDCl_3_, 400 MHz) *δ* (ppm) = 7.42 (d, *J* = 7.3 Hz, 1H); 7.29–7.26 (m, 4H); 7.23–7.20 (m, 3H); 3.76 (q, *J* = 7.0 Hz, 2H); 2.69 (t, *J* = 7.4 Hz, 2H); 2.37 (s, 3H); 2.31 (s, 3H); 1.47 (quint, *J* = 7.4 Hz, 2H); 1.31–1.21 (m, 2H); 1.14 (t, *J* = 7.0 Hz, 3H); 0.82 (t, *J* = 7.4 Hz, 3H). ^13^C NMR (CDCl_3_, 100 MHz) *δ* (ppm) = 145.28, 137.67, 137.11, 136.51, 134.07, 130.96, 130.69, 130.07, 129.92, 128.34, 127.96, 125.41, 125.17, 119.99, 68.96, 32.35, 29.70, 26.97, 22.73, 20.49, 20.46, 15.56, 13.54. MS (rel. int., %) *m*/*z*: 492 (M^+^, 87.4), 246 (94.4), 207 (93.9), 183 (58.5), 91 (41.9), 44 (100.0). HRMS (APCI-QTOF) calculated mass for C_24_H_28_OSe_2_ [M]^+^: 492.0470, found: 492.0477.

**3-(Butylselanyl)-4-methoxy-2,5-di-2-tolylselenophene 4n**: Yield: 0.030 g (25%); yellow oil. ^1^H NMR (CDCl_3_, 400 MHz) *δ* (ppm) = 7.44 (d, *J* = 7.2 Hz, 1H); 7.29–7.21 (m, 7H); 3.56 (s, 3H); 2.67 (t, *J* = 7.3 Hz, 2H); 2.36 (s, 3H); 2.31 (s, 3H); 1.49 (quint, *J* = 7.3 Hz, 2H); 1.29–1.22 (m, 2H); 0.82 (t, *J* = 7.3 Hz, 3H). ^13^C NMR (CDCl_3_, 100 MHz) *δ* (ppm) = 153.39, 145.68, 137.76, 137.09, 136.39, 133.94, 130.97, 130.70, 130.07, 129.93, 128.38, 128.09, 127.08, 125.45, 125.17, 119.40, 60.66, 32.36, 27.07, 22.71, 20.50, 20.39, 13.53. ^77^Se NMR (CDCl_3_, 76 MHz) *δ* (ppm) = 617.2, 210.3. MS (rel. int., %) *m*/*z*: 478 (M^+^, 84.8), 416 (9.0), 341 (37.6), 310 (9.4), 143 (100.0), 115 (88.7), 91 (55.7), 44 (67.6). HRMS (APCI-QTOF) calculated mass for C_23_H_27_OSe_2_ [M + H]^+^: 479.0391, found: 479.0388.

**3-(Butylselanyl)-2,5-bis(2-chlorophenyl)-4-ethoxyselenophene 4o**: Yield (determined by ^1^H NMR): 15%; yellow oil. Mixture of compounds **4m** and diyne **1i** (ratio 69:31%). Asterisk denotes the chemical shifts of the diyne **1i**. ^1^H NMR (CDCl_3_, 400 MHz) *δ* (ppm) = 7.63–7.61 (m, 1H); 7.57* (d, *J* = 7.4 Hz, 2H); 7.47 (d, *J* = 6.4 Hz, 2H); 7.42* (d, *J* = 7.4 Hz, 2H); 7.32–7.29 (m, 5H), 7.26–7.22* (m, 2H); 3.87 (q, *J* = 7.0 Hz, 2H); 2.75 (t, *J* = 7.3 Hz, 2H); 1.51 (quint, *J* = 7.3 Hz, 2H); 1.31–1.22 (m, 2H); 1.18 (t, *J* = 7.0 Hz, 3H); 0.82 (t, *J* = 7.3 Hz, 3H). ^13^C NMR (CDCl_3_, 100 MHz) *δ* (ppm) = 154.35, 143.72, 136.95*, 135.77, 134.36*, 134.09, 133.98, 133.43, 132.57, 132.41, 130.28*, 129.76, 129.56, 129.44*, 129.15, 126.54*, 126.46, 126.22, 121.80*, 121.34, 79.40*, 78.37*, 69.23, 32.23, 27.29, 22.69, 15.54, 13.53. ^77^Se NMR (CDCl_3_, 76 MHz) *δ* (ppm) = 623.1, 208.5. MS (rel. Int.) *m*/*z*: 532 (M^+^; 13.2), 441 (14.3), 203 (58.8), 123 (17.9), 41 (100.0). HRMS (APCI-QTOF) calculated mass for C_22_H_23_Cl_2_OSe_2_ [M + H]^+^: 532.9446, found: 532.9438.

**3-(Butylselanyl)-2,5-bis(2-chlorophenyl)-4-methoxyselenophene 4p**: Yield: 0.032 g (25%); yellow oil. ^1^H NMR (CDCl_3_, 400 MHz) *δ* (ppm) = 7.63–7.57 (m, 1H); 7.49–7.46 (m, 2H); 7.43–7.40 (m, 1H); 7.35–7.29 (m, 4H); 3.66 (s, 3H); 2.74 (t, *J* = 7.4 Hz, 2H); 1.51 (quint, *J* = 7.4 Hz, 2H); 1.31–1.22 (m, 2H); 0.82 (t, *J* = 7.4 Hz, 3H). ^13^C NMR (CDCl_3_, 100 MHz) *δ* (ppm) = 154.89, 144.03, 135.67, 134.36, 134.08, 133.34, 132.54, 132.44, 130.27, 129.77, 129.59, 129.30, 126.52, 126.23, 125.06, 120.82, 60.77, 32.25, 27.39, 22.69, 13.53. ^77^Se NMR (CDCl_3_, 76 MHz) *δ* (ppm) = 626.5, 207.6. MS (rel. Int.) *m*/*z*: 518 (M^+^; 9.1), 427 (22.0), 203 (20.3), 123 (14.4), 41 (100.0). HRMS (APCI-QTOF) calculated mass for C_21_H_21_Cl_2_OSe_2_ [M + H]^+^: 518.9290, found: 518.9276.

## 4. Conclusions

In this work, we developed an alternative and transition-metal-free procedure for accessing 3,4-bis(butylselanyl)selenophenes by the electrophilic cyclization of 1,3-diynes with dibutyl diselenide using Oxone^®^ as a green oxidant and acetonitrile as solvent. In addition, we demonstrated for the first time the synthesis of 3-(butylselanyl)-4-alkoxyselenophenes starting from several 1,3-diynes and dibutyl diselenide in the presence of Oxone^®^ using aliphatic alcohols as solvent/nucleophiles. This protocol was sensitive to electronic effect in the 1,3-diynes, as well as to steric effects of the alkyl chain of the alcohols.

## Figures and Tables

**Table 1:**
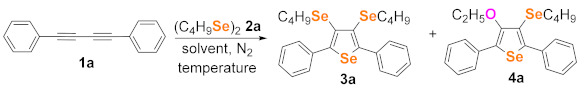
Optimization of the reaction conditions ^a^.

#	2a (mmol)	Oxone^®^ (mmol)	Solvent	Temp.	Time (h)	Yield 3a (%) ^b^	Yield 4a (%) ^b^
1	0.50	0.50	CH_3_CN	80 °C	72	58	-
2 ^c^	0.50	0.50	CH_3_CN	-	2	15	-
3	0.50	0.50	DMF	110 °C	72	NR ^d^	-
4	0.50	0.50	PEG-400	90 °C	72	NR ^d^	-
5	0.50	0.50	glycerol	90 °C	72	NR ^d^	-
6	0.50	0.50	EtOH	reflux	24	15	43
7	0.50	0.75	CH_3_CN	80 °C	48	78	-
8	0.50	1.0	CH_3_CN	80 °C	48	75	-
9	0.50	0.25	CH_3_CN	80 °C	72	38	-
10	0.50	0.38	CH_3_CN	80 °C	72	50	-
11	0.38	0.50	EtOH	reflux	24	12	48
12	0.25	0.50	EtOH	reflux	36	5	35
13	0.38	0.25	EtOH	reflux	48	8	39
14	0.38	0.38	EtOH	reflux	36	7	42
15	0.38	0.75	EtOH	reflux	24	3	70
16	0.38	1.0	EtOH	reflux	24	5	65
17 ^e^	0.38	0.75	EtOH	reflux	48	8	15

^a^ Reaction conditions: A mixture of 1,3-diyne **1a** (0.25 mmol), Oxone^®^, and dibutyl diselenide **2a** in the solvent (3.0 mL) under nitrogen atmosphere was stirred at the temperature and time indicated. The progress of the reaction was monitored by TLC. ^b^ Isolated yield after purification by preparative thin-layer chromatography. ^c^ Reaction performed under ultrasonic irradiation (US) at 60% of amplitude. ^d^ Product **3a** was not formed, and the starting materials were recovered. ^e^ Reaction performed in open flask.

**Table 2:** Synthesis of 3,4-bis(butylselanyl)selenophenes **3** ^a,b^.

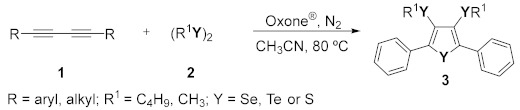
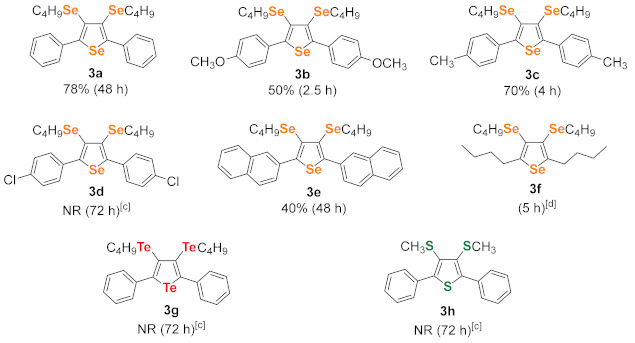

^a^ Reaction conditions: A mixture of diyne **1** (0.25 mmol), dialkyl dichalcogenide **2** (0.50 mmol), and Oxone^®^ (0.75 mmol) in acetonitrile (3.0 mL) under nitrogen atmosphere was stirred at 80 °C by the time indicated. The progress of the reaction was monitored by TLC. ^b^ Isolated yields after purification by preparative thin-layer chromatography. ^c^ No product was detected and the starting materials were recovered. ^d^ The starting materials were completely consumed and a complex mixture of decomposition products was formed.

**Table 3:** Synthesis of 4-alkoxyselenophenes **4** ^a,b^.

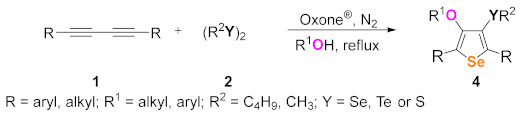
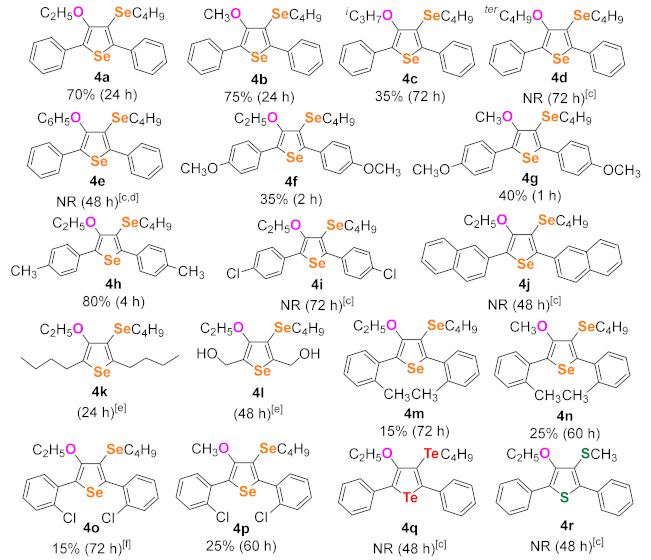

^a^ Reaction conditions: A mixture of diyne **1** (0.25 mmol), dialkyl dichalcogenide **2** (0.38 mmol), and Oxone^®^ (0.75 mmol) in the alcohol (3.0 mL) under nitrogen atmosphere was stirred under reflux by the time indicated. The progress of the reaction was monitored by TLC. ^b^ Isolated yields after purification by preparative thin-layer chromatography. ^c^ No product was detected, and the starting materials were recovered. ^d^ Reaction performed using acetonitrile (3.0 mL) as solvent and 0.50 mmol of phenol. ^e^ The starting materials were completely consumed, providing a complex mixture of products. ^f^ Conversion determined by ^1^H NMR.

## Data Availability

The data presented in this study are available on request from the corresponding authors.

## Supplementary Materials

The following are available online. General procedure for the synthesis of (2,2-dibromovinyl)benzene **6a–i**, general procedure for the synthesis of symmetric 1,3-diynes **1a–i** and copies of 1H, 13C, and 77Se NMR spectra of the prepared compounds. Figure S1: 1H NMR (400 MHz, CDCl3) spectrum of compound **3a**, Figure S2: 13C NMR (100 MHz, CDCl3) spectrum of compound **3a**, Figure S3: 1H NMR (400 MHz, CDCl3) spectrum of compound **3b**, Figure S4: 13C NMR (100 MHz, CDCl3) spectrum of compound **3b**, Figure S5: 77Se NMR (76 MHz, CDCl3) spectrum of compound **3b**, Figure S6: 1H NMR (400 MHz, CDCl3) spectrum of compound **3c**, Figure S7: 13C NMR (100 MHz, CDCl3) spectrum of compound **3c**, Figure S8: 77Se NMR (76 MHz, CDCl3) spectrum of compound **3c**, Figure S9: 1H NMR (400 MHz, CDCl3) spectrum of compound **3e**, Figure S10: 13C NMR (100 MHz, CDCl3) spectrum of compound **3e**, Figure S11: 77Se NMR (76 MHz, CDCl3) spectrum of compound **3e**, Figure S12: 1H NMR (400 MHz, CDCl3) spectrum of compound **4a**, Figure S13: 13C NMR (100 MHz, CDCl3) spectrum of compound **4a**, Figure S14: 1H NMR (400 MHz, CDCl3) spectrum of compound **4b**, Figure S15: 13C NMR (100 MHz, CDCl3) spectrum of compound **4b**, Figure S16: 77Se NMR (76 MHz, CDCl3) spectrum of compound **4b**, Figure S17: 1H NMR (400 MHz, CDCl3) spectrum of compound **4c**, Figure S18: 13C NMR (100 MHz, CDCl3) spectrum of compound **4c**, Figure S19: 77Se NMR (76 MHz, CDCl3) spectrum of compound **4c**, Figure S20: 1H NMR (400 MHz, CDCl3) spectrum of compound **4f**, Figure S21: 13C NMR (100 MHz, CDCl3) spectrum of compound **4f**, Figure S22: 1H NMR (400 MHz, CDCl3) spectrum of compound **4g**, Figure S23: 13C NMR (100 MHz, CDCl3) spectrum of compound **4g**, Figure S24: 1H NMR (400 MHz, CDCl3) spectrum of compound **4h**, Figure S25: 13C NMR (100 MHz, CDCl3) spectrum of compound **4h**, Figure S26: 77Se NMR (76 MHz, CDCl3) spectrum of compound **4h**, Figure S27: COSY NMR-2D (400 MHz, CDCl3) spectrum of compound **4h**, Figure S28: 1H-13C HSQC NMR-2D (400 MHz, CDCl3) spectrum of compound **4h**, Figure S29: 1H-13C HMBC NMR-2D (400 MHz, CDCl3) spectrum of compound **4h**, Figure S30: 1H NMR (400 MHz, CDCl3) spectrum of compound **4m**, Figure S31: 13C NMR (100 MHz, CDCl3) spectrum of compound **4m**, Figure S32: 1H NMR (400 MHz, CDCl3) spectrum of compound **4n**, Figure S33: 13C NMR (100 MHz, CDCl3) spectrum of compound **4n**, Figure S34: 77Se NMR (76 MHz, CDCl3) spectrum of compound **4n**, Figure S35: 1H NMR (400 MHz, CDCl3) spectrum of compound **4o**, Figure S36: 13C NMR (100 MHz, CDCl3) spectrum of compound **4o**, Figure S37: 77Se NMR (76 MHz, CDCl3) spectrum of compound **4o**, Figure S38: 1H NMR (400 MHz, CDCl3) spectrum of compound **4p**, Figure S39: 13C NMR (100 MHz, CDCl3) spectrum of compound **4p**, Figure S40: 77Se NMR (76 MHz, CDCl3) spectrum of compound **4p**.

## Sample Availability

Samples of the compounds are available with the authors.
