# Supplementary material for: Synthesis of 3,4-Bis(Butylselanyl)Selenophenes and 4-Alkoxyselenophenes Promoted by Oxone®
Source: Molecules. 2021 Apr 19;26(8):2378. doi: 10.3390/molecules26082378 (PMC8073937; doi:10.3390/molecules26082378)
Supplement: Supplementary file 1 [file molecules-26-02378-s001.zip › molecules-1174846-supplementary.pdf]

Synthesis of 3,4-Bis(butylselanyl)selenophenes and 4-Alkoxyselenophenes Promoted by Oxone®

Paola S. Hellwig,[1] Jonatan S. Guedes,[1] Angelita M. Barcellos,[1] Gelson Perin,*[1] and Eder J. Lenardão*[1]

[1] LASOL - CCQFA, Universidade Federal de Pelotas - UFPel, P.O. Box 354, 96010-900 Pelotas, RS, Brazil.

* Correspondence: gelson_perin@ufpel.edu.br (G.P.); lenardao@ufpel.edu.br (E.J.L.); Tel.: +55-53-32757356 (G.P. & E.J.L.)

Contents

General procedure for the synthesis of (2,2-dibromovinyl)benzene **6a-i**.................................. S2

General procedure for the synthesis of symmetric 1,3-diynes **1a-i**... S2

References.. S3

Copies of (^{1}H, ^{13}C and ^{77}Se) NMR spectra S4

General procedure for the synthesis of (2,2-dibromovinyl)benzene 6a-i

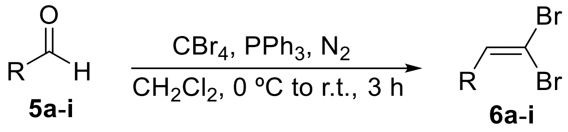

The compounds were prepared according to a published procedure.[1,2] To a round-bottomed flask containing the appropriate aldehyde **5** (10.0 mmol) in dry dichloromethane (30.0 mL) under nitrogen atmosphere and magnetic stirring at room temperature, a solution of CBr_4 (3.93 g, 12.0 mmol) in dichloromethane (20.0 mL) was added. The reaction system was cooled at 0 °C and Ph_3P (6.55 g; 25.0 mmol) was added dropwise during 30 min. Then, the resulting mixture was stirred for additional 3 h at room temperature. After that, hexane (50.0 mL) was added, and the crude mixture was filtered using silica gel. The eluted part was concentrated for further purification by column chromatography (100-200 mesh silica gel) using hexane as the eluent. Yield: 75-95%.

General procedure for the synthesis of symmetric 1,3-diynes 1a-i

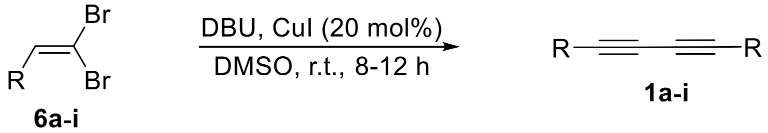

1a R = C_6H_5, 90%
1b R = 4-$CH_3OC_6H_4$, 85%
1c R = 4-$CH_3C_6H_4$, 80%
1d R = 4-ClC_6H_4, 65%
1e R = 2-naphthyl, 85%
1f R = C_4H_9, 45%
1g R = C_2H_2OH, 48%
1h R = 2-$CH_3C_6H_4$, 60%
1i R = 2-ClC_6H_4, 50%

The compounds were prepared according to a published procedure.[3] To a sealed tube were added DMSO (2.0 mL), the appropriate (2,2 dibromovinyl)benzene (1.0 mmol), CuI (20 mol%, 0.038 g), DBU (2.0 mmol, 0.304 g). The mixture was stirred at room temperature (25 °C) for 8-12 h. Then a saturated sodium chloride solution (10.0 mL) was added, and the product was extracted with ethyl acetate (3x 15.0 mL). The organic layer was separated, dried with $MgSO_4$ and concentrated under vacuum. The residue was purified

by column chromatography using silica gel and eluted with hexane/ethyl acetate in different proportions. Yields: 45-90%.

References:

1. Corey, E.J.; Fuchs, P.L. A Synthetic Method for Formyl→Ethynyl Conversion (RCHO→RC-CH or RC-CR′). *Tetrahedron Lett*. **1972**, *36*, 3769-3772; doi: 10.1016/S0040-4039(01)94157-7.
2. Huh, D.H.; Jeong, J.S.; Lee, H.B.; Ryu, H.; Kim, Y.G. An Efficient Method for One-carbon Elongation of Aryl Aldehydes via their Dibromoalkene Derivatives. *Tetrahedron* **2002**, *58*, 9925-9932; doi: 10.1016/S0040-4020(02)01324-8.
3. Hui, J.; Chunxiang, K. Ligand-free Copper-catalyzed Synthesis of Symmetrical Diynes from 1,1-Dibromo-1-alkenes. *Chin. J. Chem.* **2011**, *29*, 592-594; doi: 10.1002/chin.201130087.

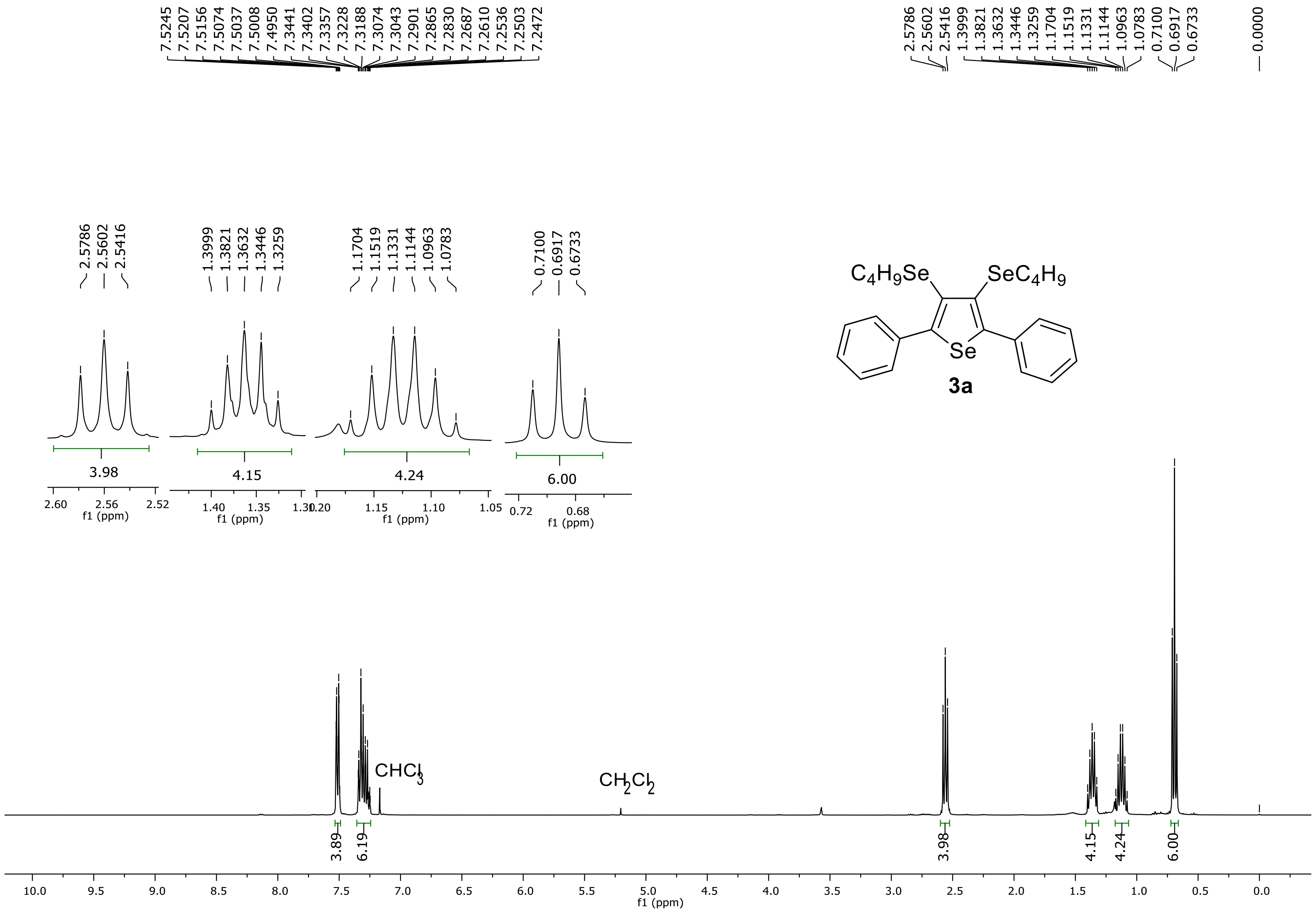

Figure S1: ^{1}H NMR (400 MHz, $CDCl_3$) spectrum of compound **3a**.

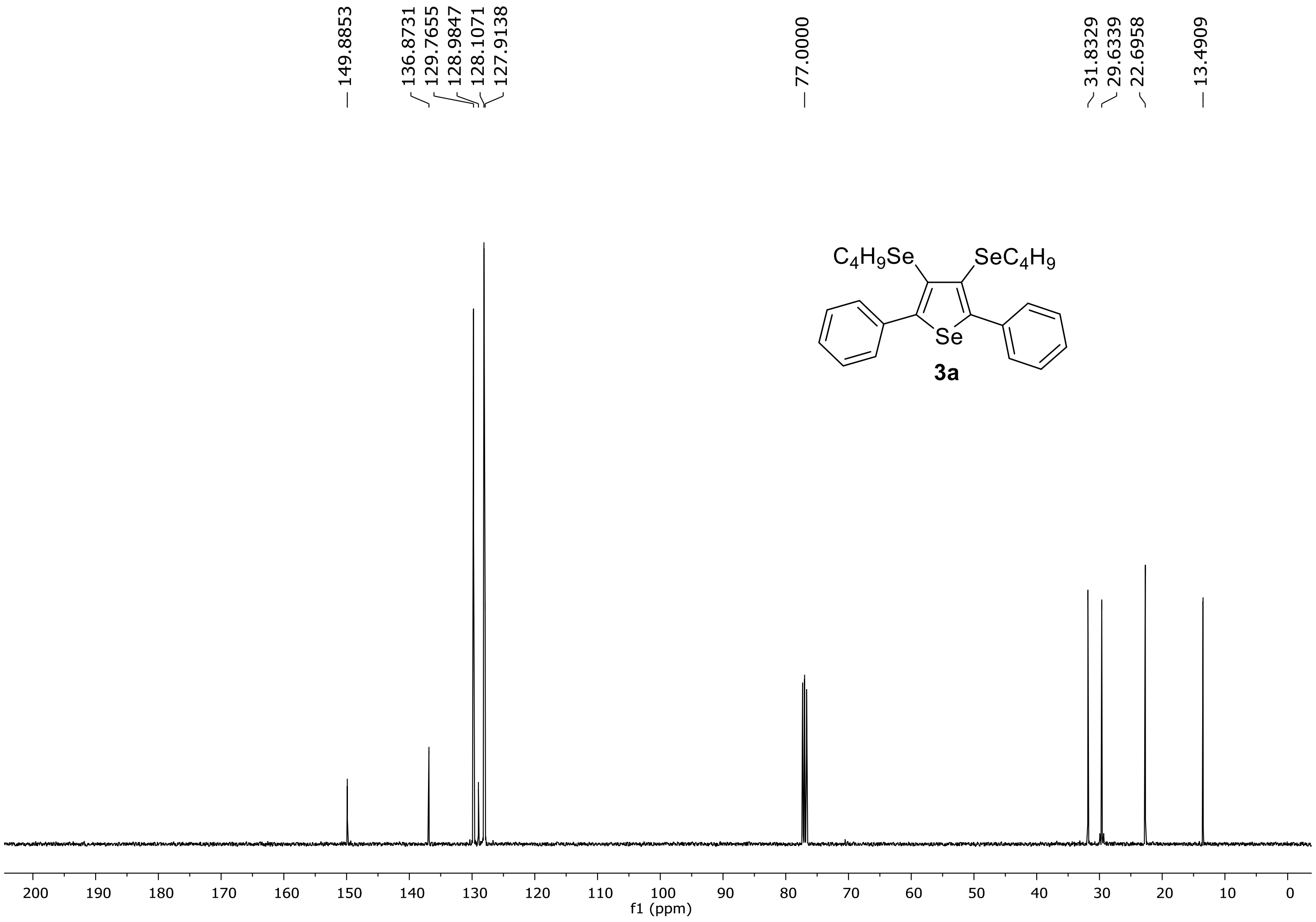

Figure S2: ^{13}C NMR (100 MHz, $CDCl_3$) spectrum of compound **3a**.

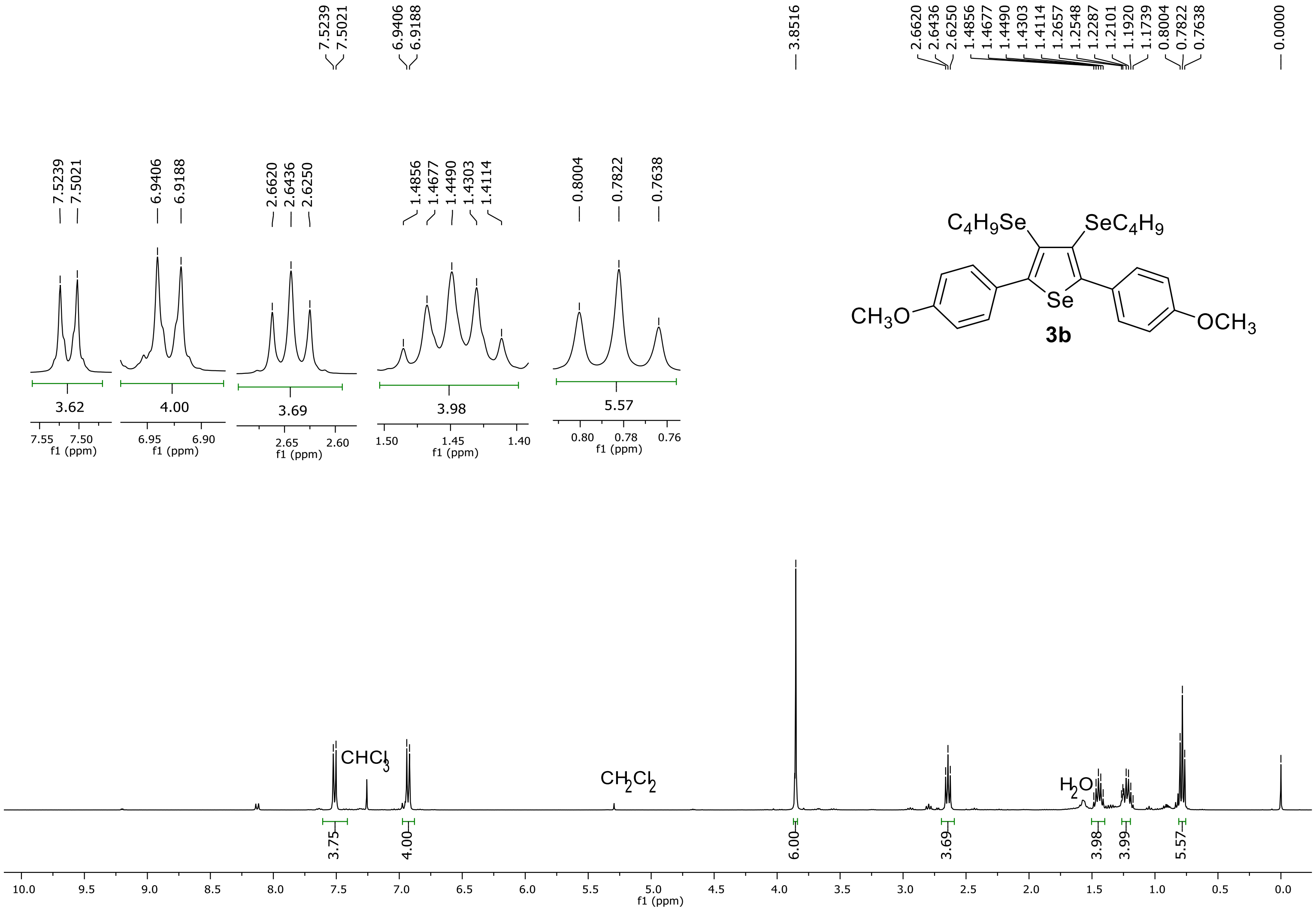

Figure S3: ^{1}H NMR (400 MHz, $CDCl_3$) spectrum of compound **3b**.

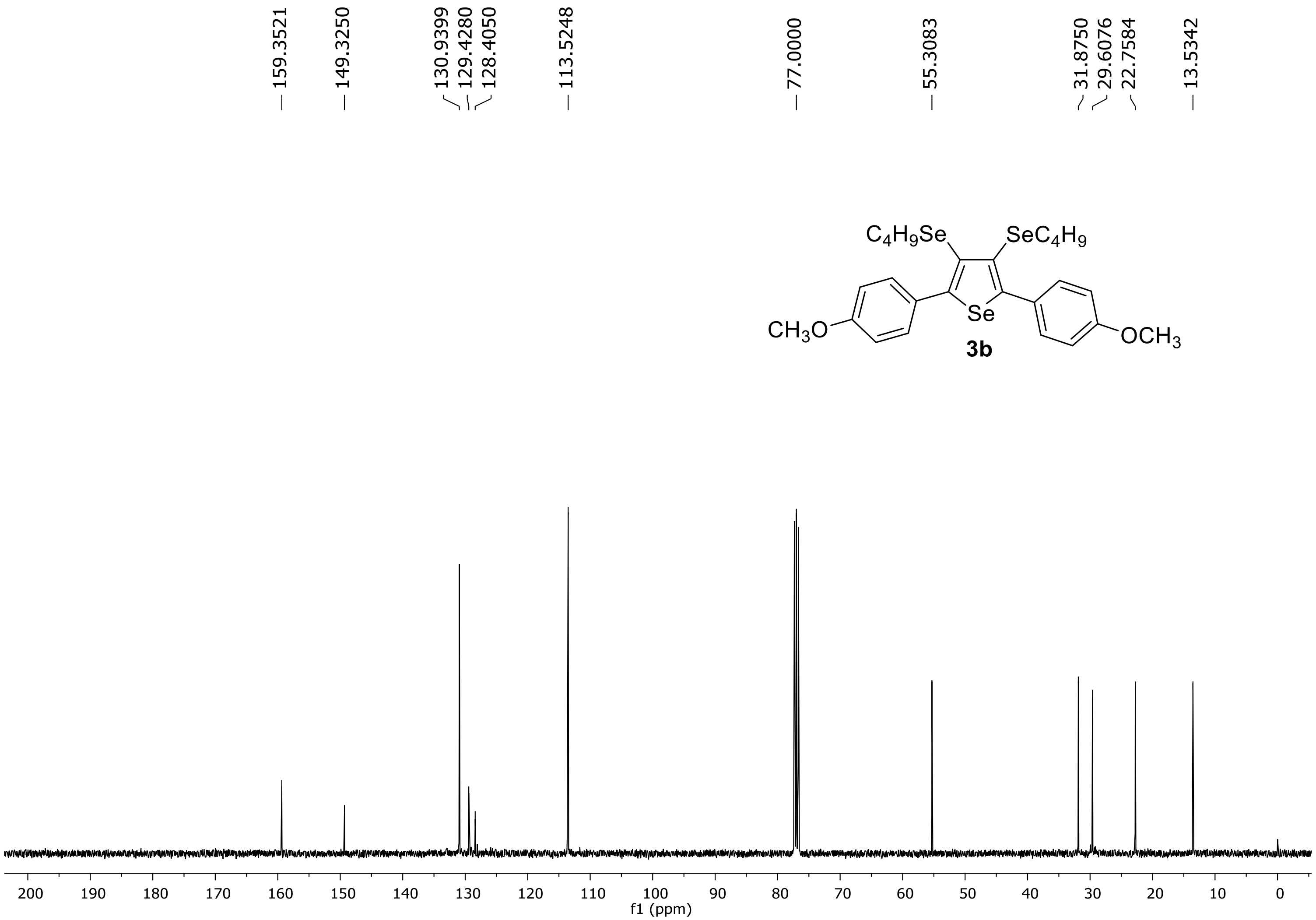

Figure S4: ^{13}C NMR (100 MHz, $CDCl_3$) spectrum of compound **3b**.

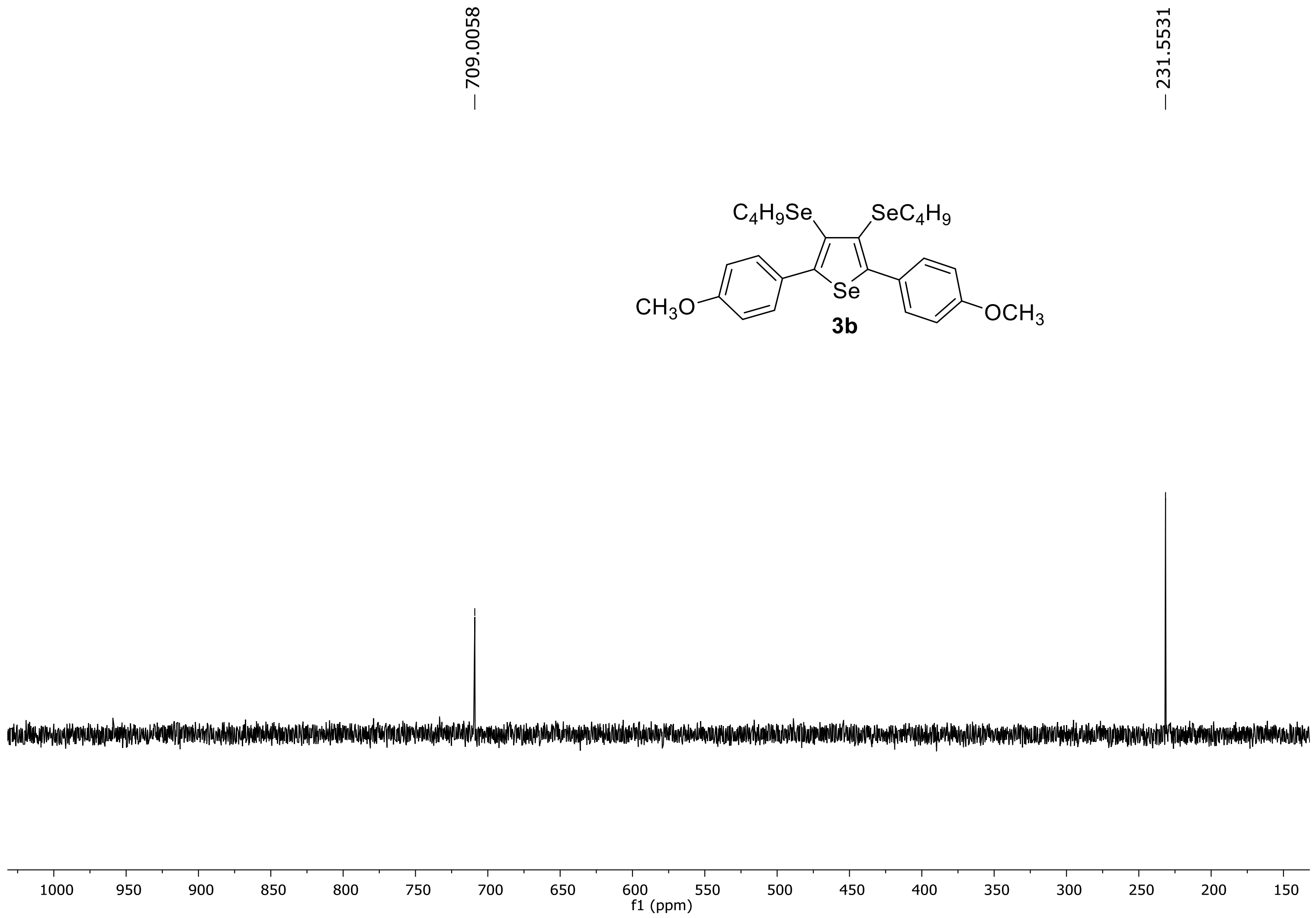

Figure S5: ^{77}Se NMR (76 MHz, $CDCl_3$) spectrum of compound **3b**.

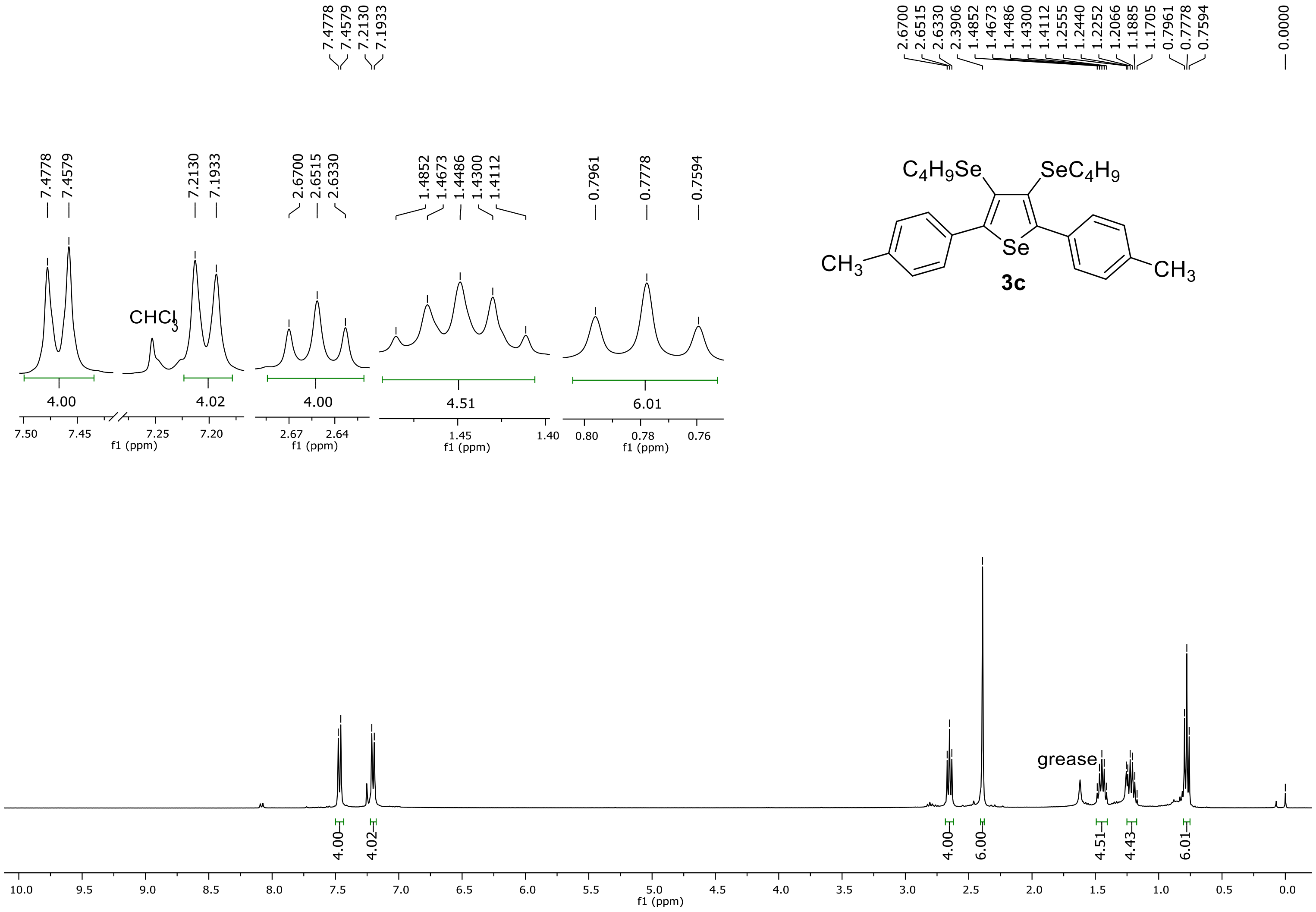

Figure S6: ^{1}H NMR (400 MHz, $CDCl_3$) spectrum of compound **3c**.

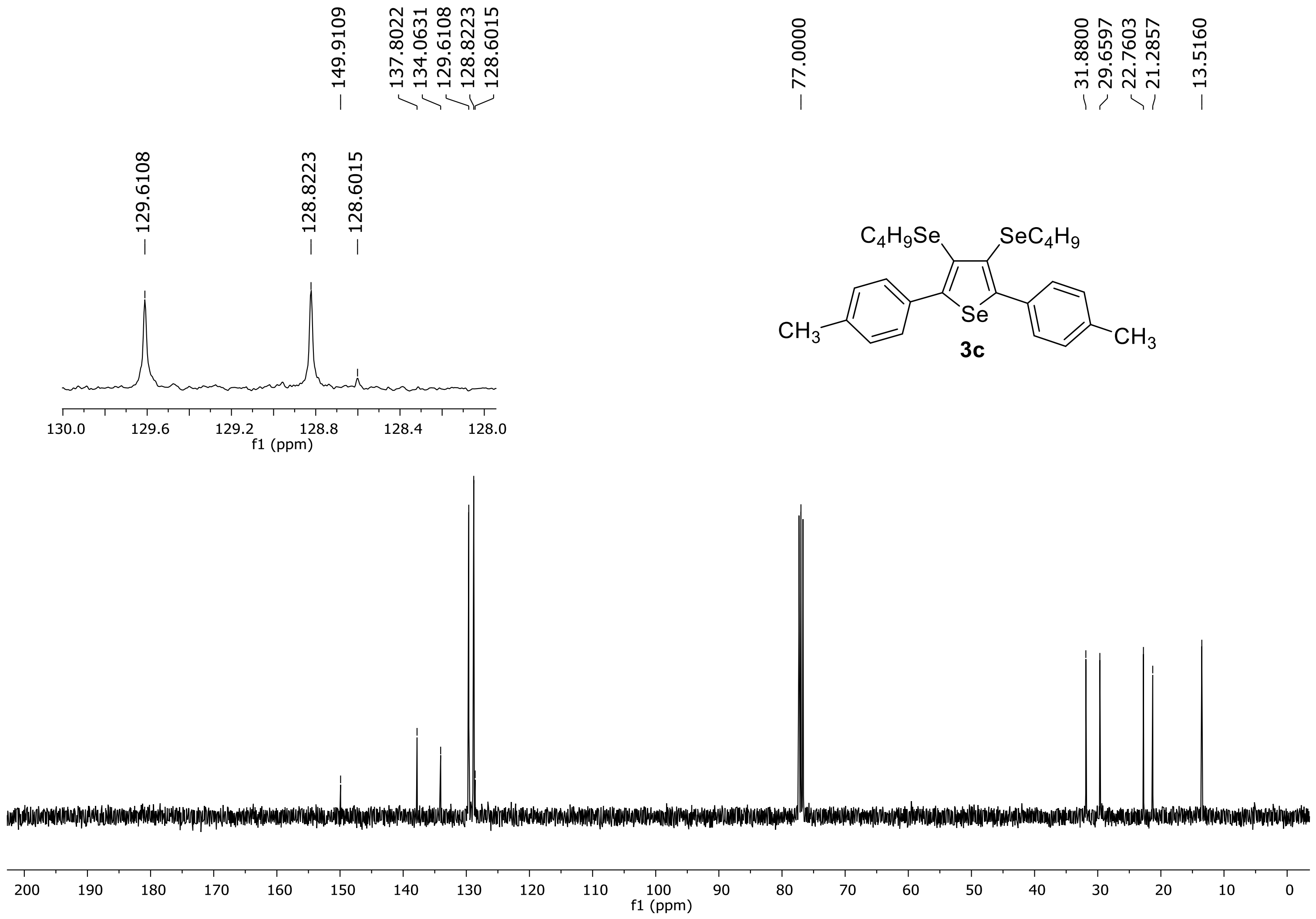

Figure S7: ^{13}C NMR (100 MHz, $CDCl_3$) spectrum of compound **3c**.

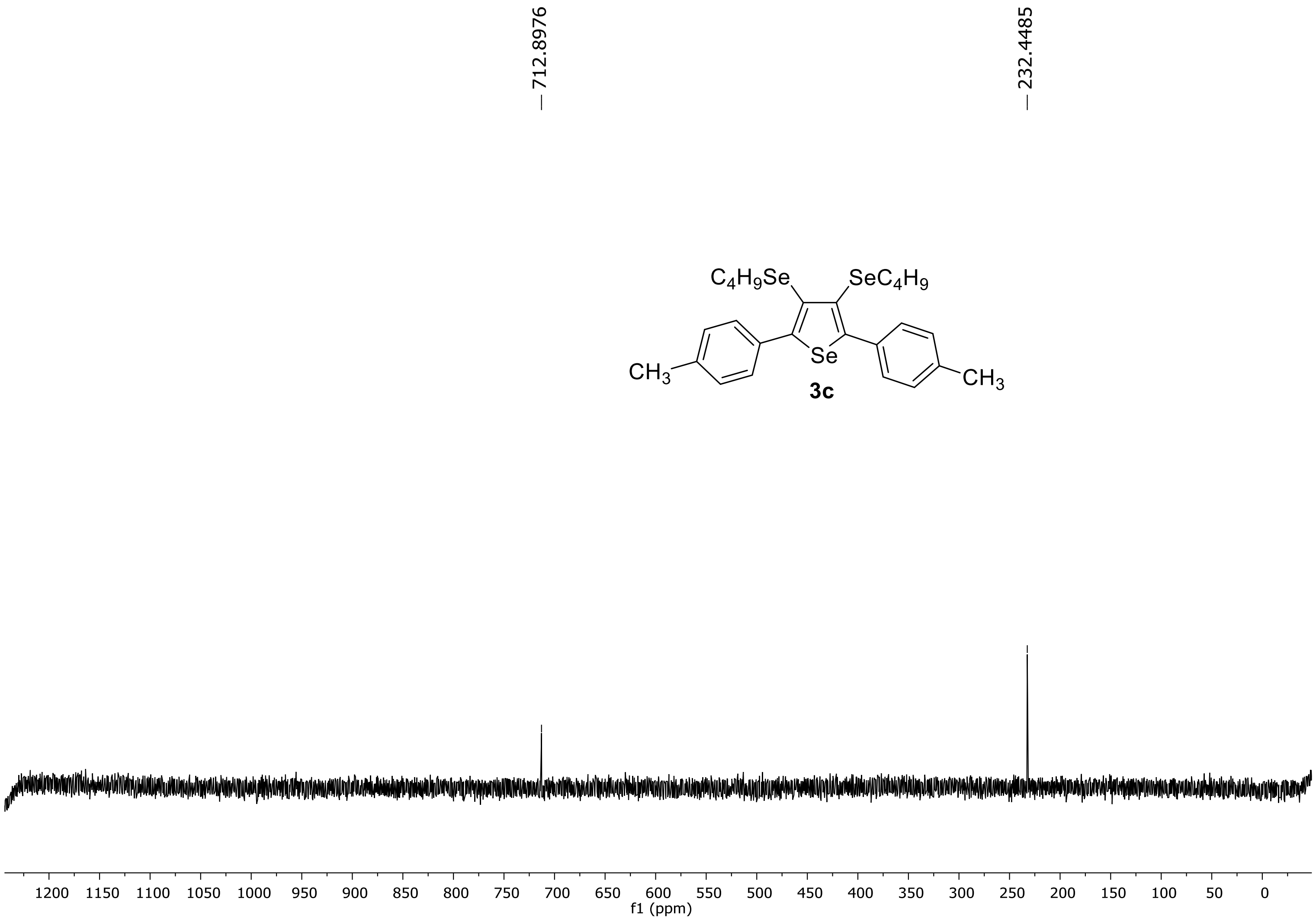

Figure S8: ^{77}Se NMR (76 MHz, $CDCl_3$) spectrum of compound **3c**.

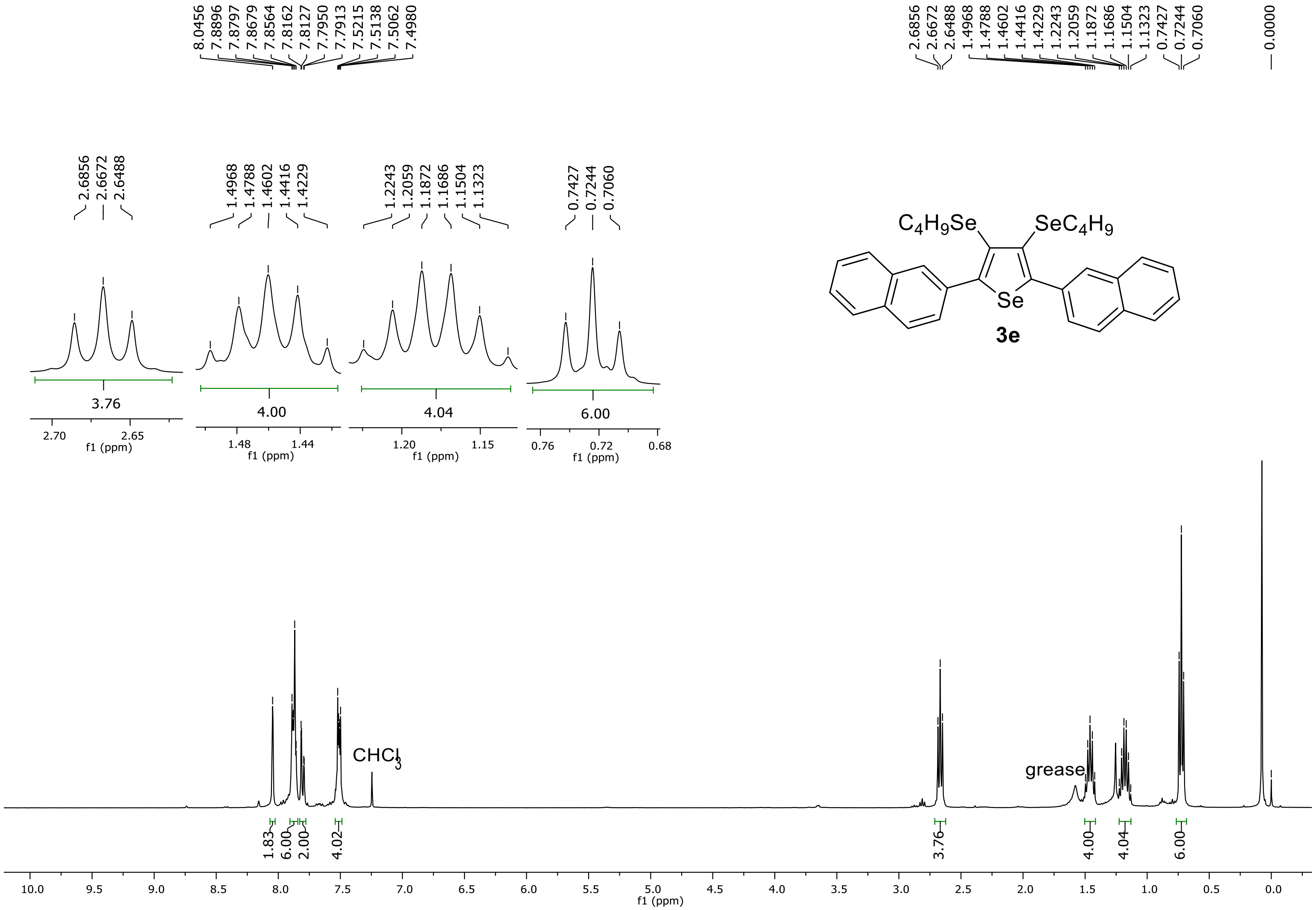

Figure S9: ^{1}H NMR (400 MHz, $CDCl_3$) spectrum of compound **3e**.

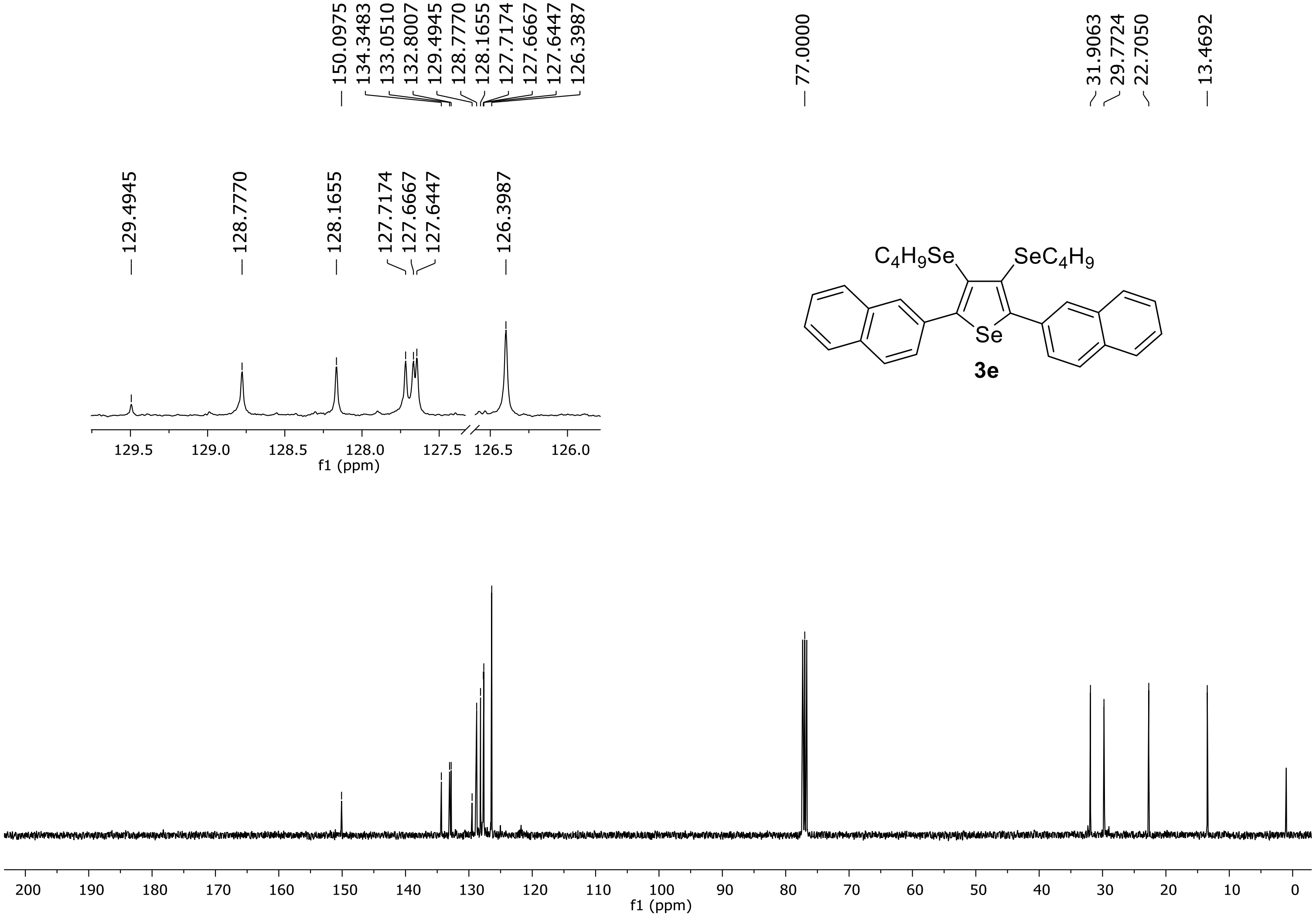

Figure S10: ^{13}C NMR (100 MHz, $CDCl_3$) spectrum of compound **3e**.

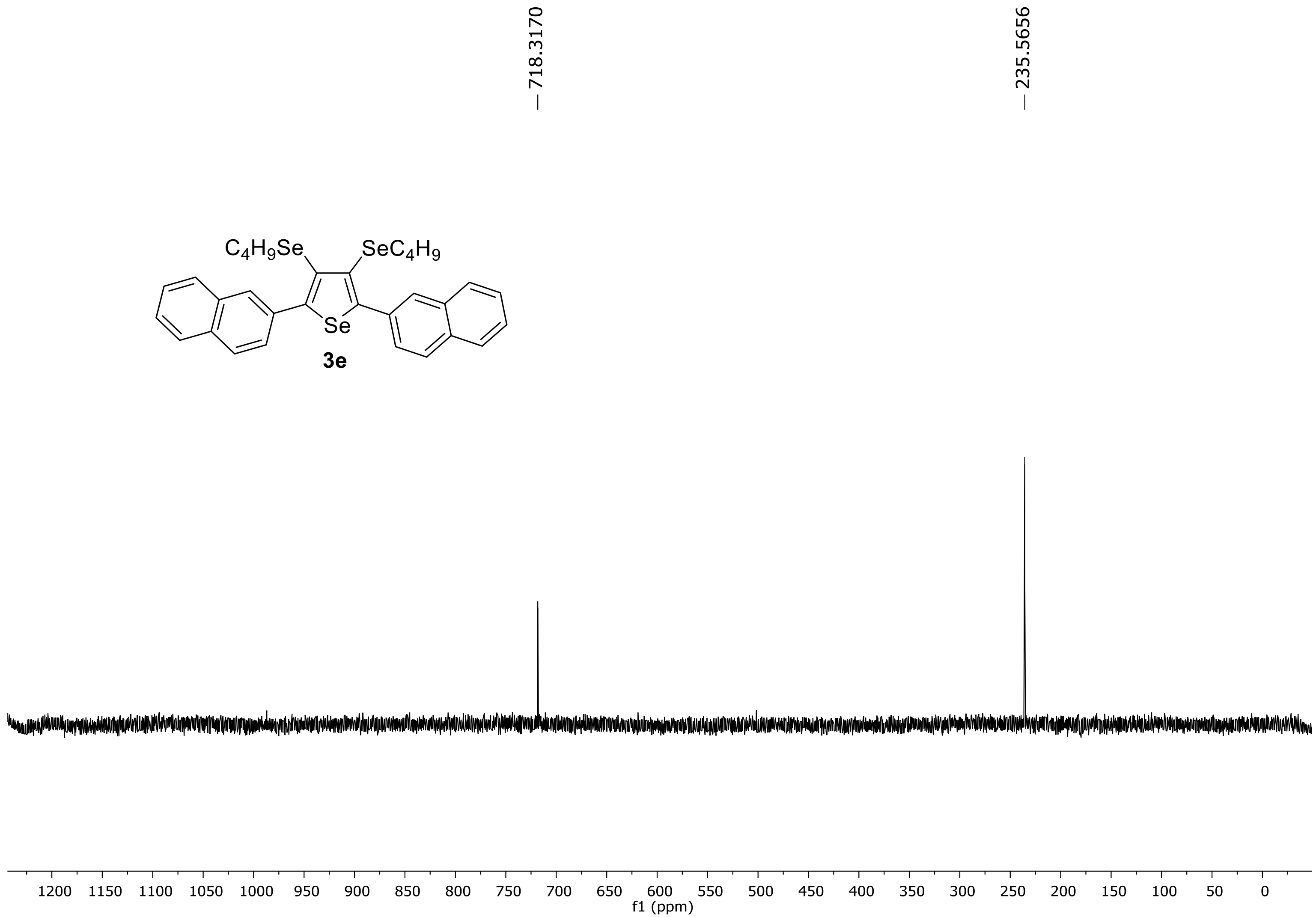

Figure S11: ^{77}Se NMR (76 MHz, $CDCl_3$) spectrum of compound **3e**.

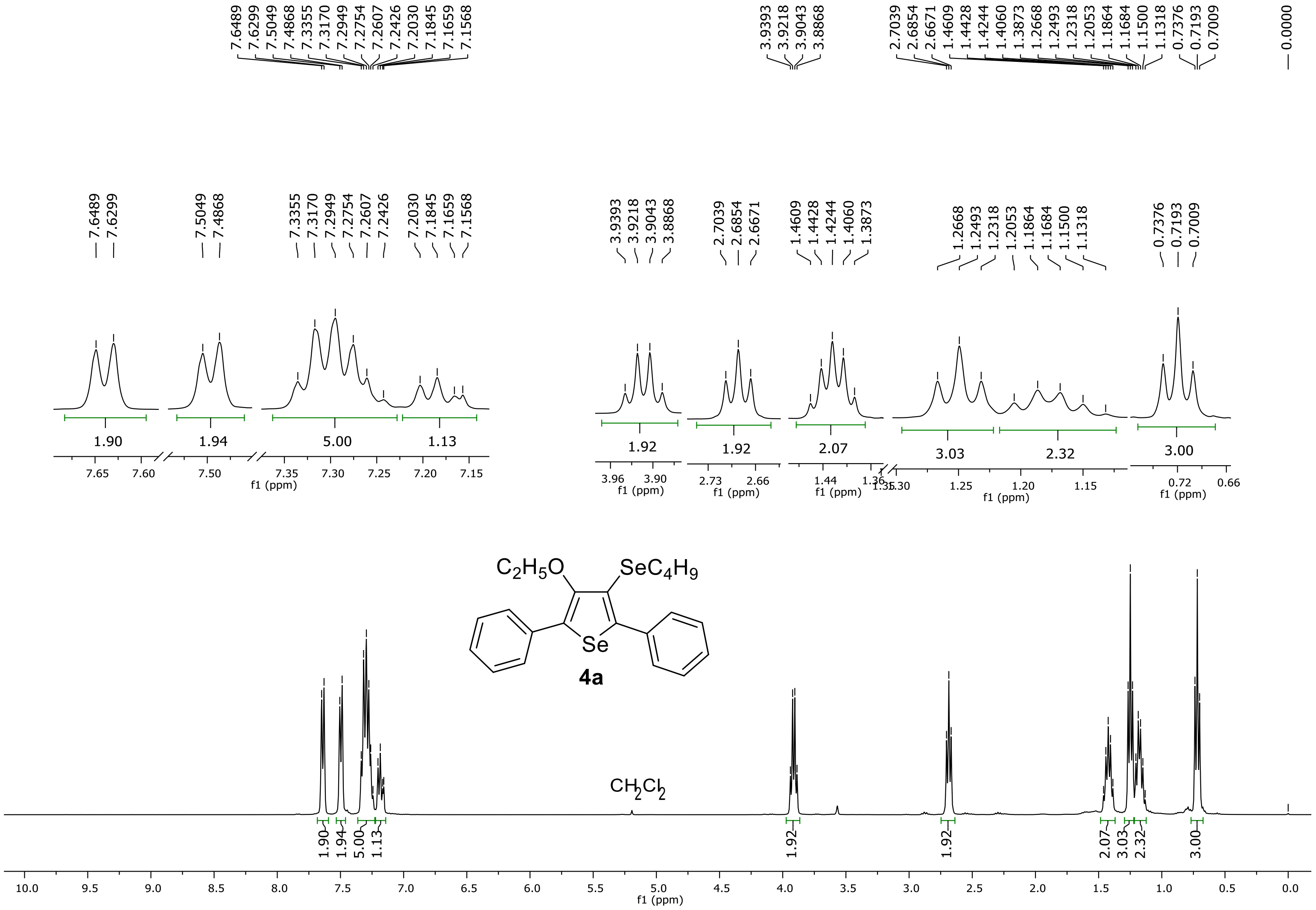

Figure S12: ^{1}H NMR (400 MHz, $CDCl_3$) spectrum of compound **4a**.

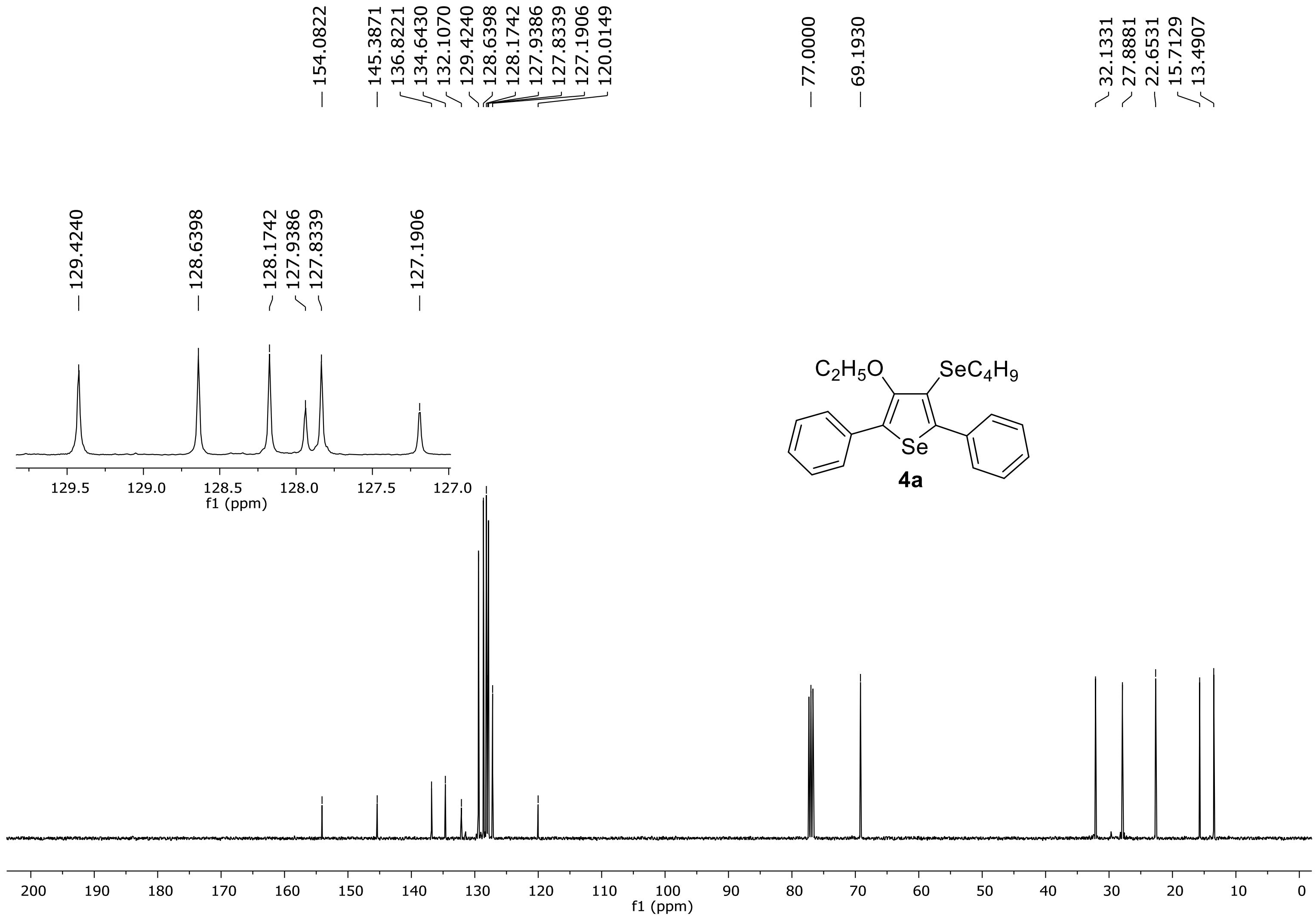

Figure S13: ^{13}C NMR (100 MHz, $CDCl_3$) spectrum of compound **4a**.

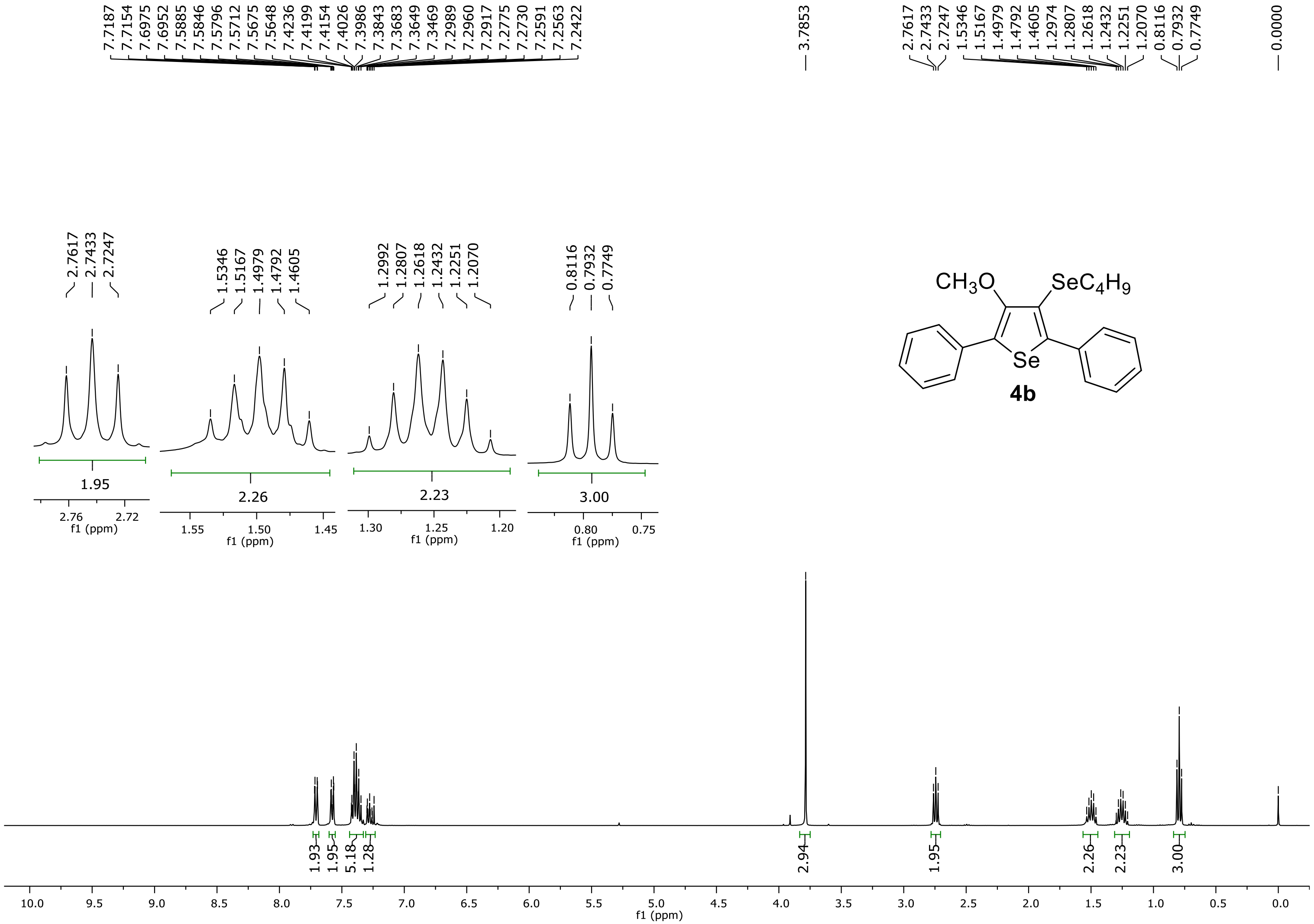

Figure S14: ^{1}H NMR (400 MHz, $CDCl_3$) spectrum of compound **4b**.

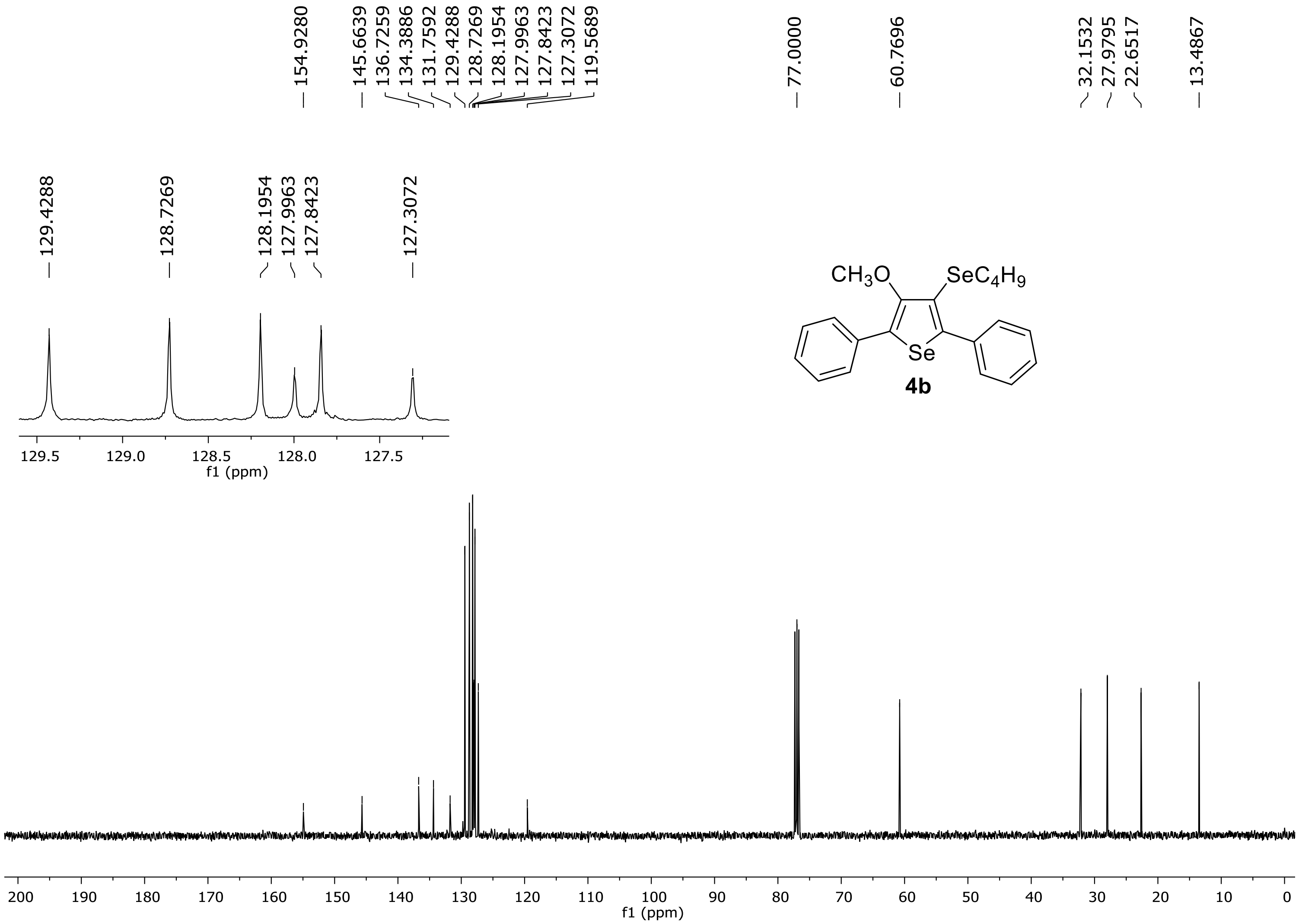

Figure S15: ^{13}C NMR (100 MHz, $CDCl_3$) spectrum of compound **4b**.

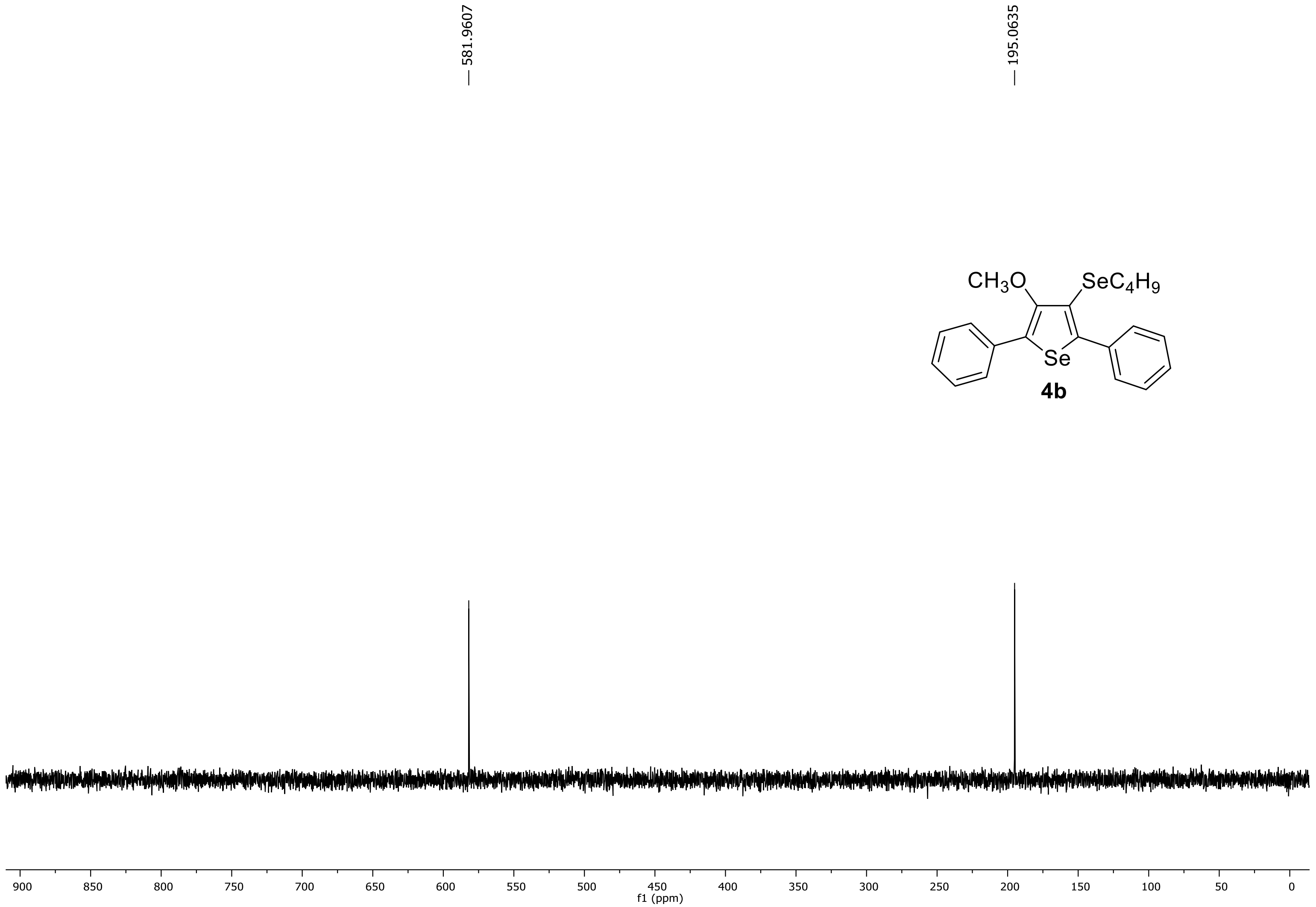

Figure S16: ^{77}Se NMR (76 MHz, $CDCl_3$) spectrum of compound **4b**.

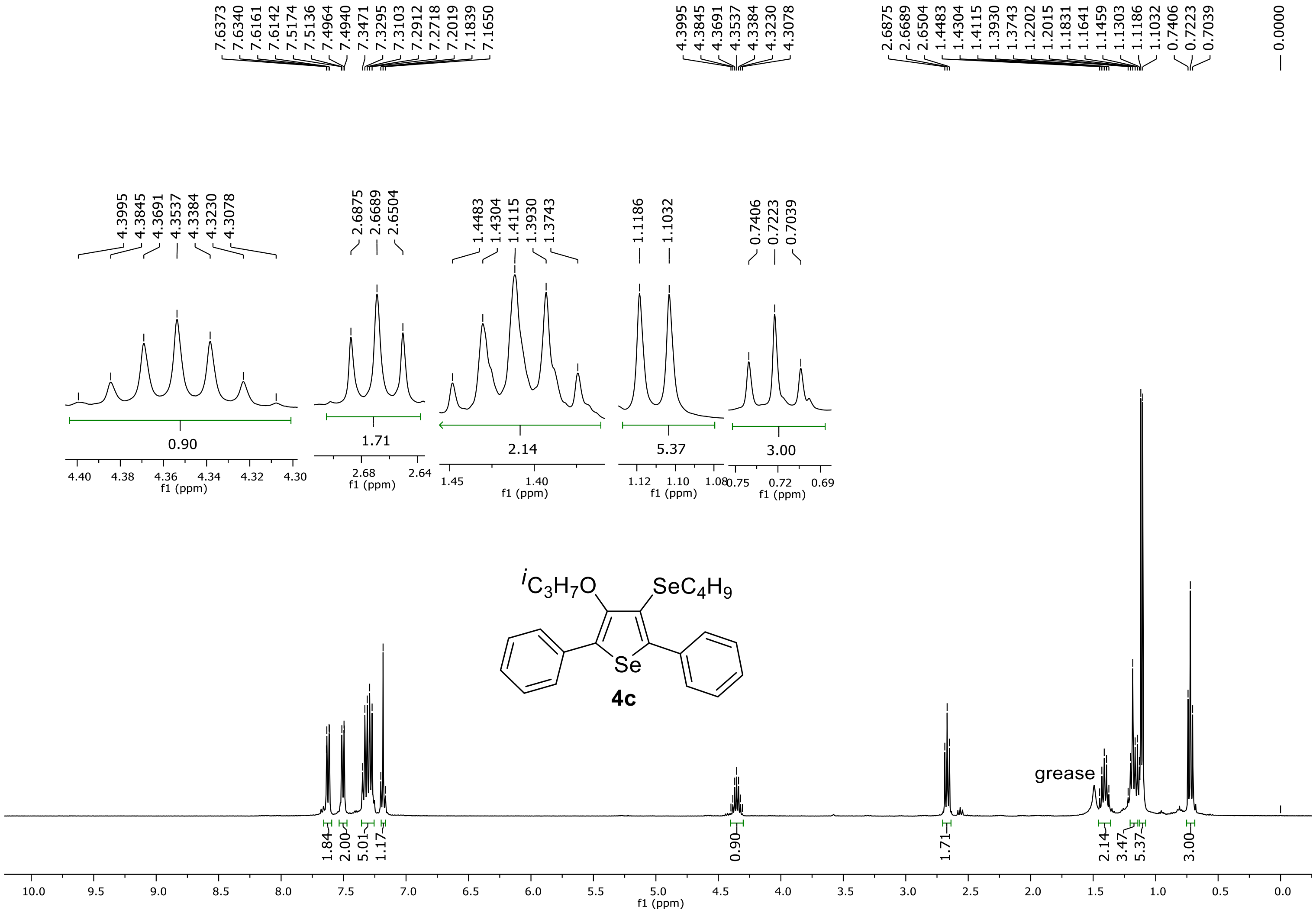

Figure S17: ^{1}H NMR (400 MHz, CDCl₃) spectrum of compound **4c**.

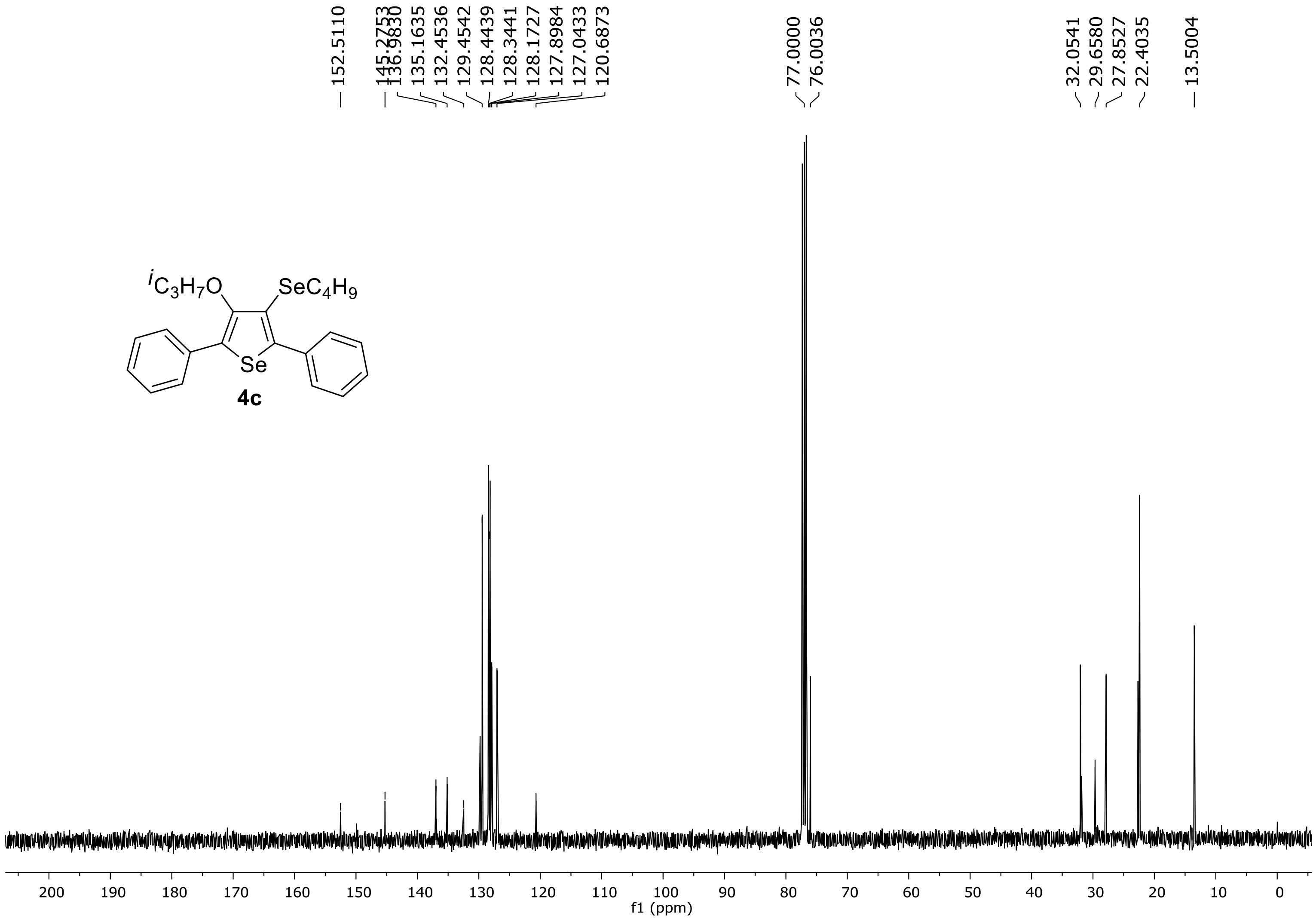

Figure S18: ^{13}C NMR (100 MHz, $CDCl_3$) spectrum of compound **4c**.

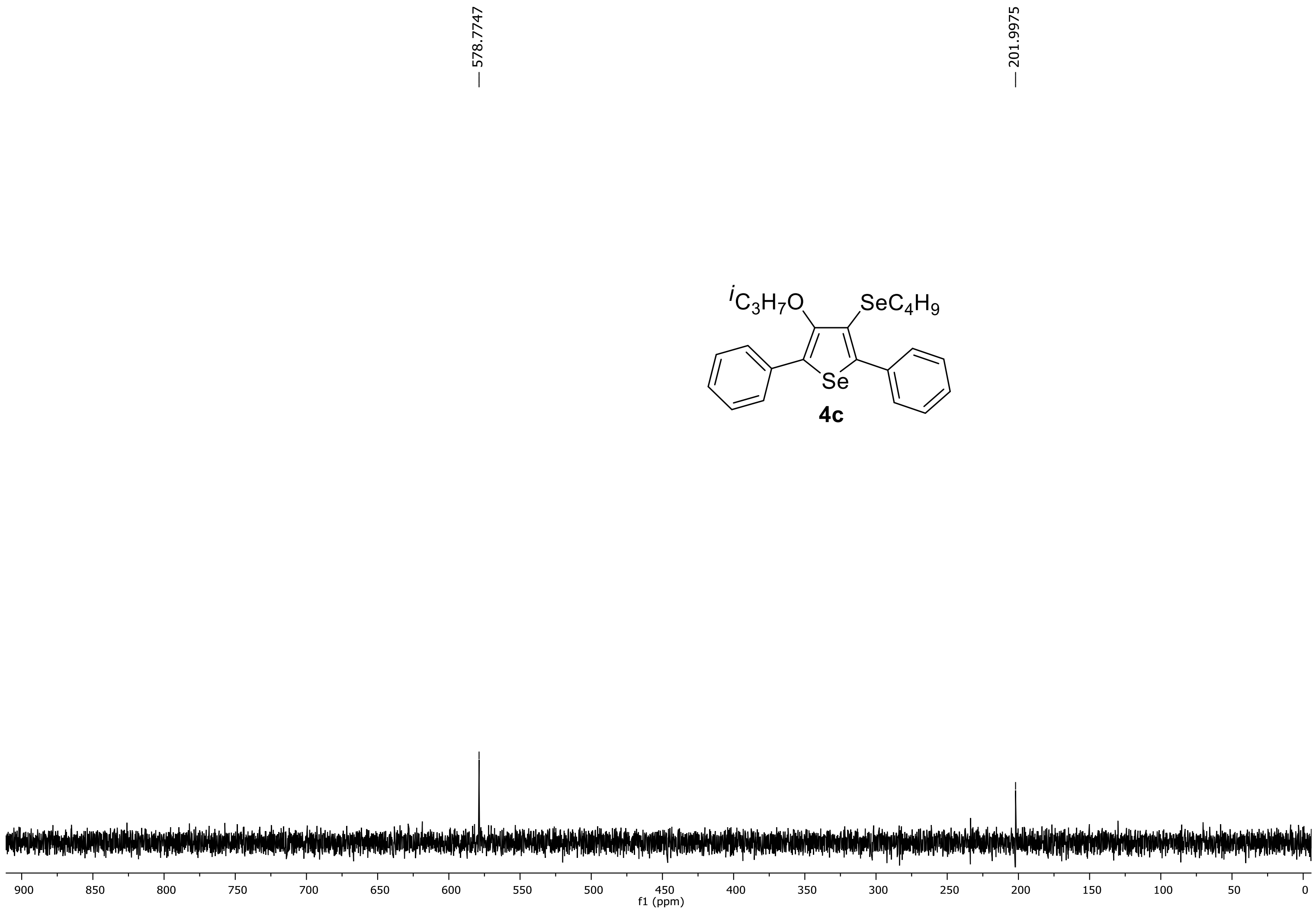

Figure S19: ^{77}Se NMR (76 MHz, $CDCl_3$) spectrum of compound **4c**.

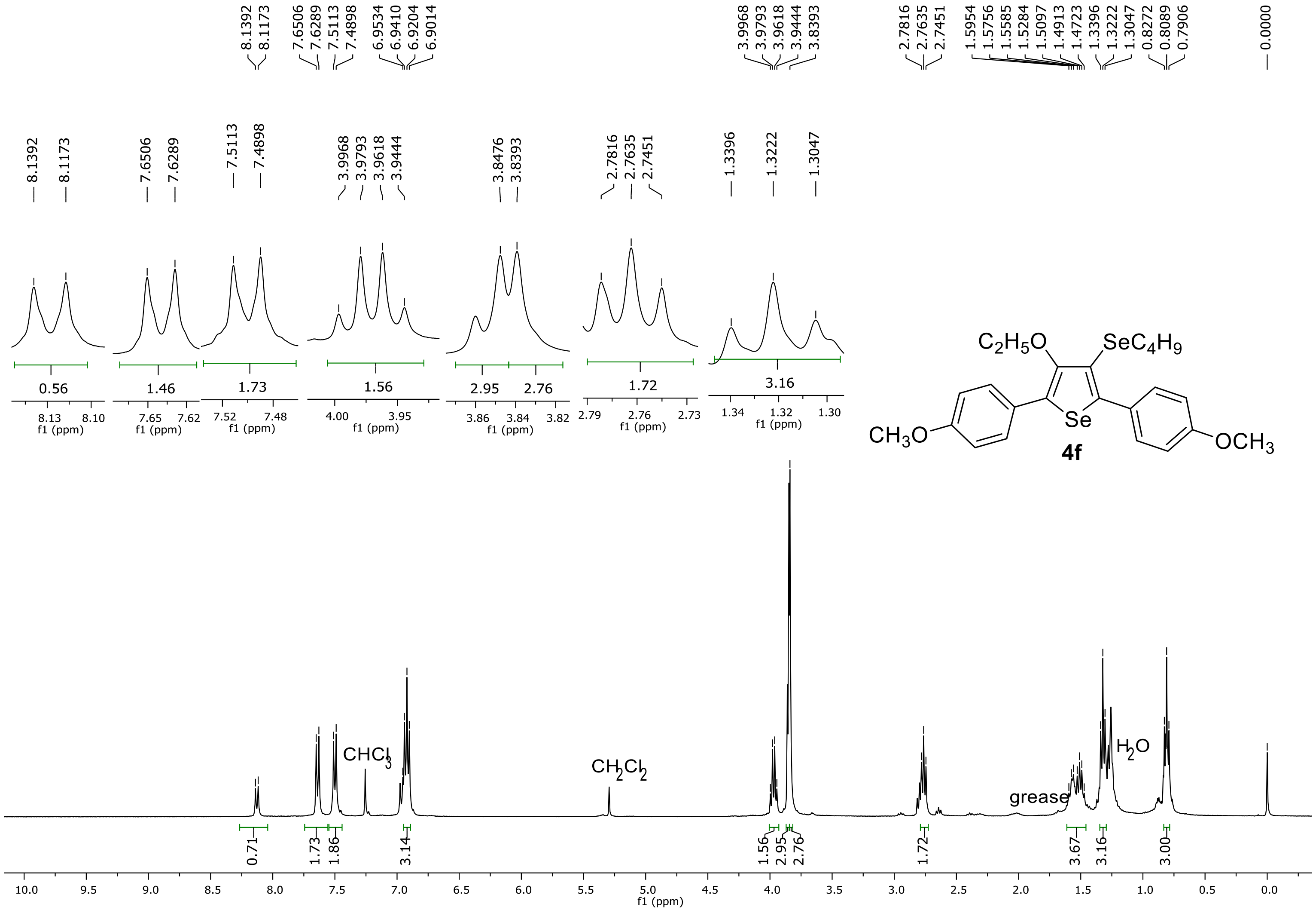

Figure S20: ^{1}H NMR (400 MHz, CDCl$_3$) spectrum of compound **4f**.

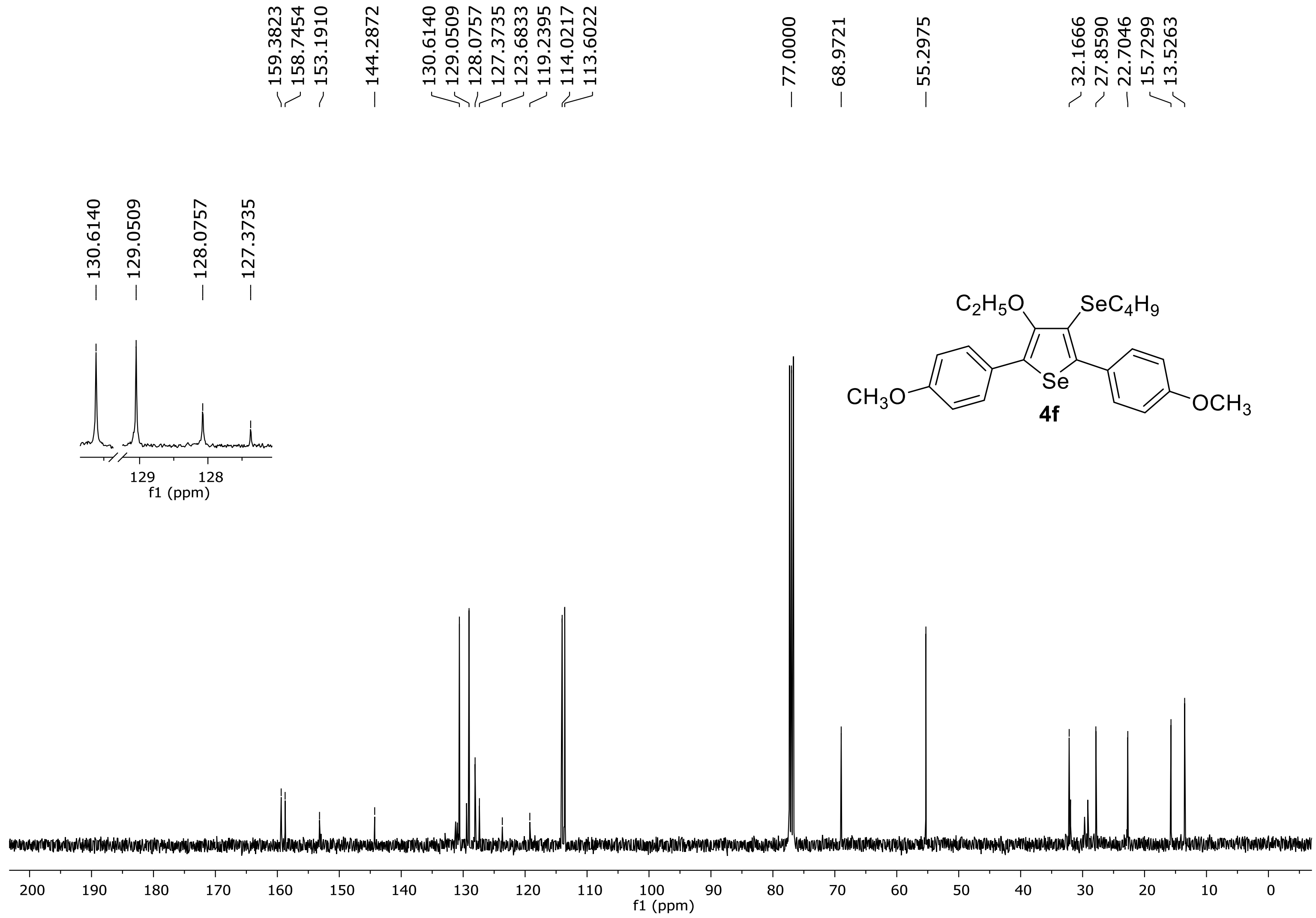

Figure S21: ${}^{13}C$ NMR (100 MHz, $CDCl_3$) spectrum of compound **4f**.

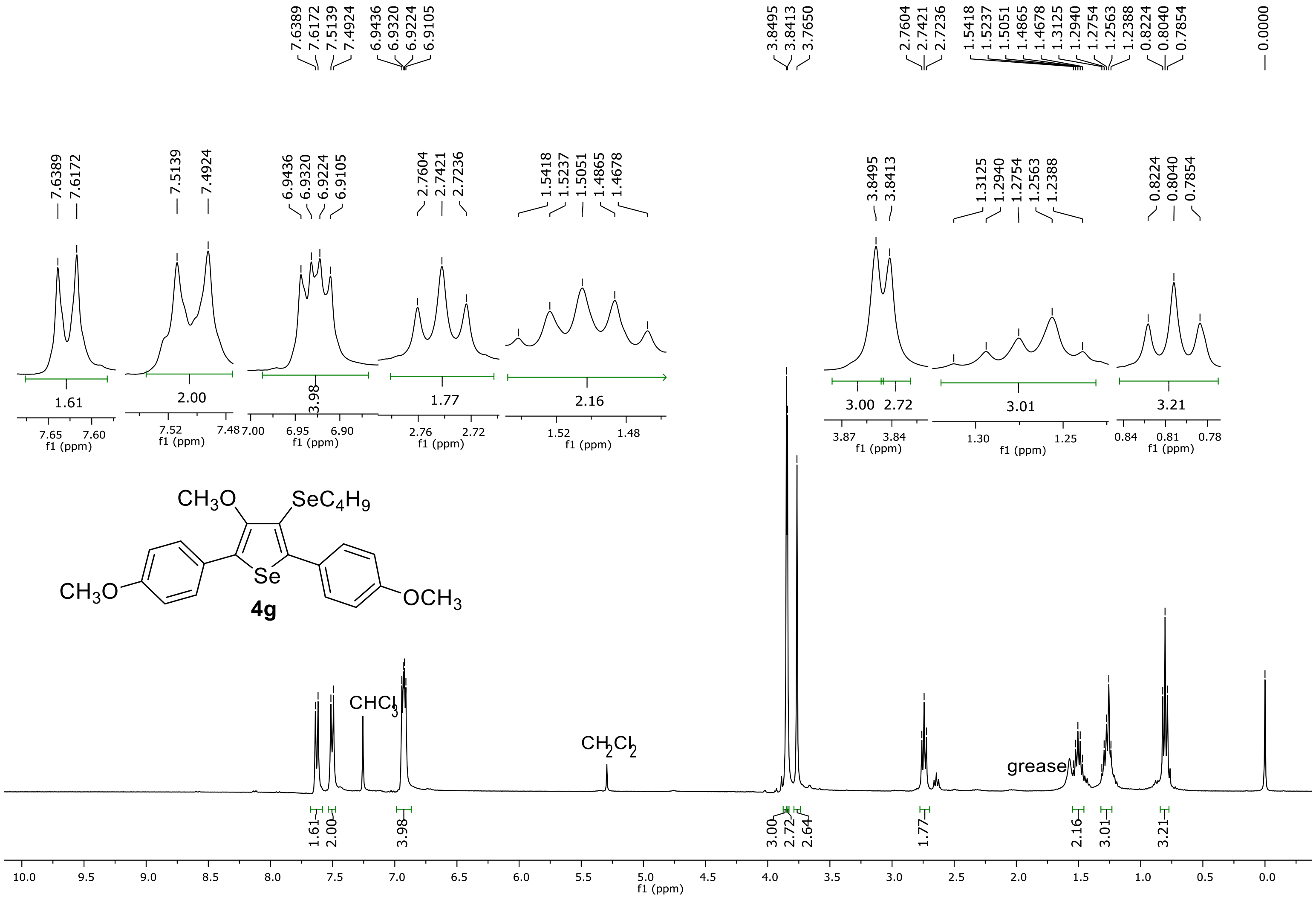

Figure S22: ^{1}H NMR (400 MHz, CDCl3) spectrum of compound **4g**.

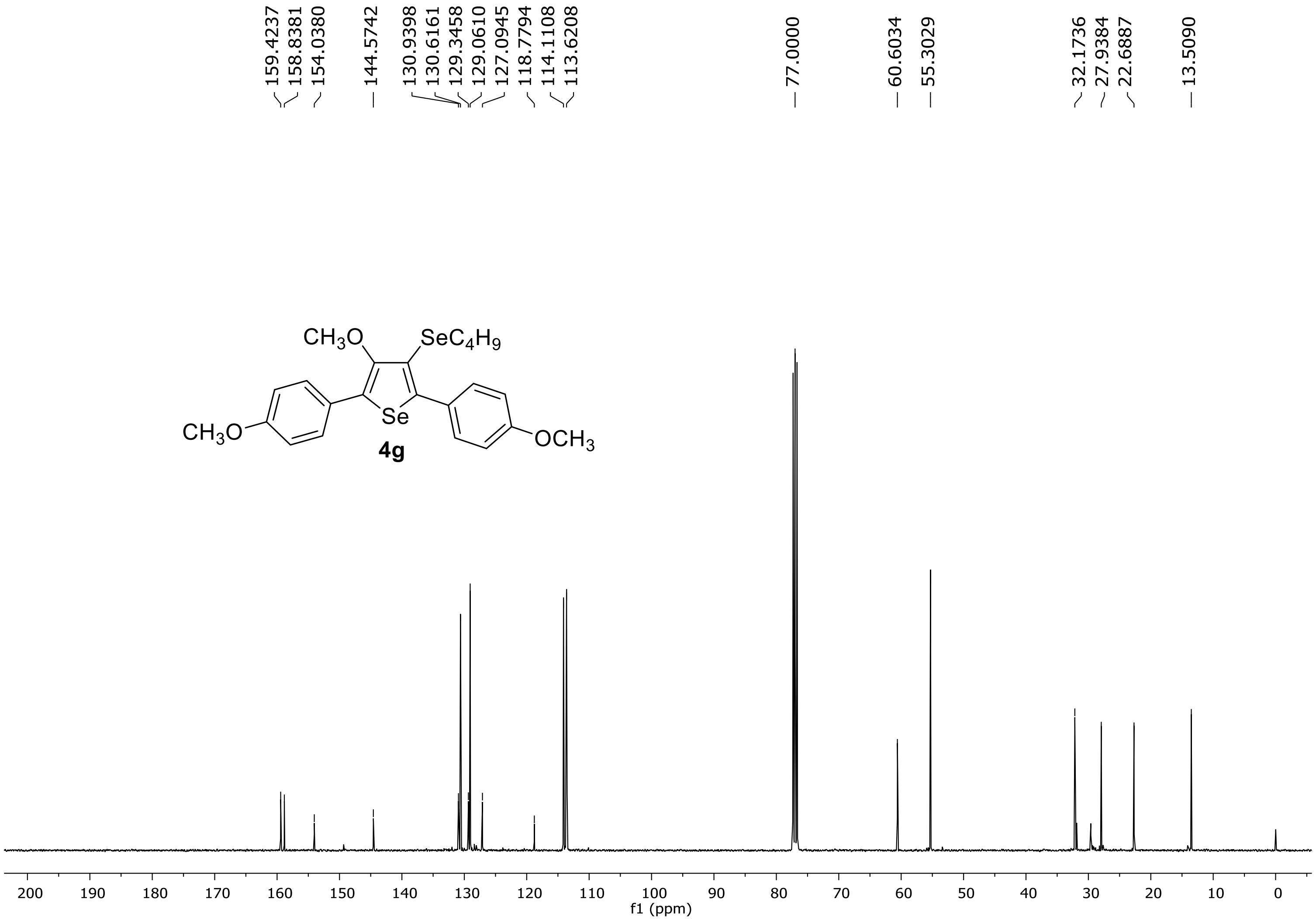

Figure S23: ^{13}C NMR (100 MHz, $CDCl_3$) spectrum of compound **4g**.

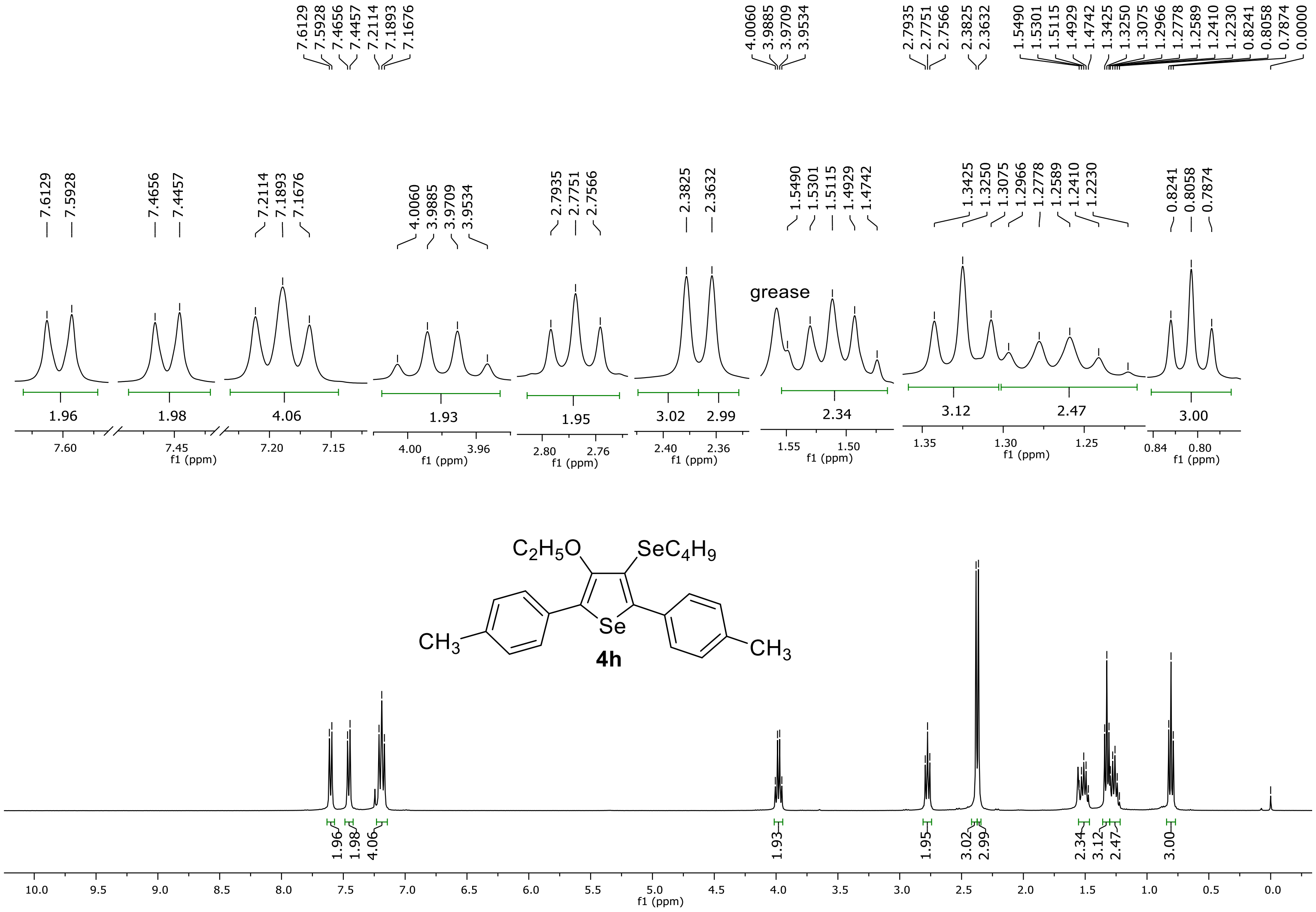

Figure S24: ^{1}H NMR (400 MHz, $CDCl_3$) spectrum of compound **4h**.

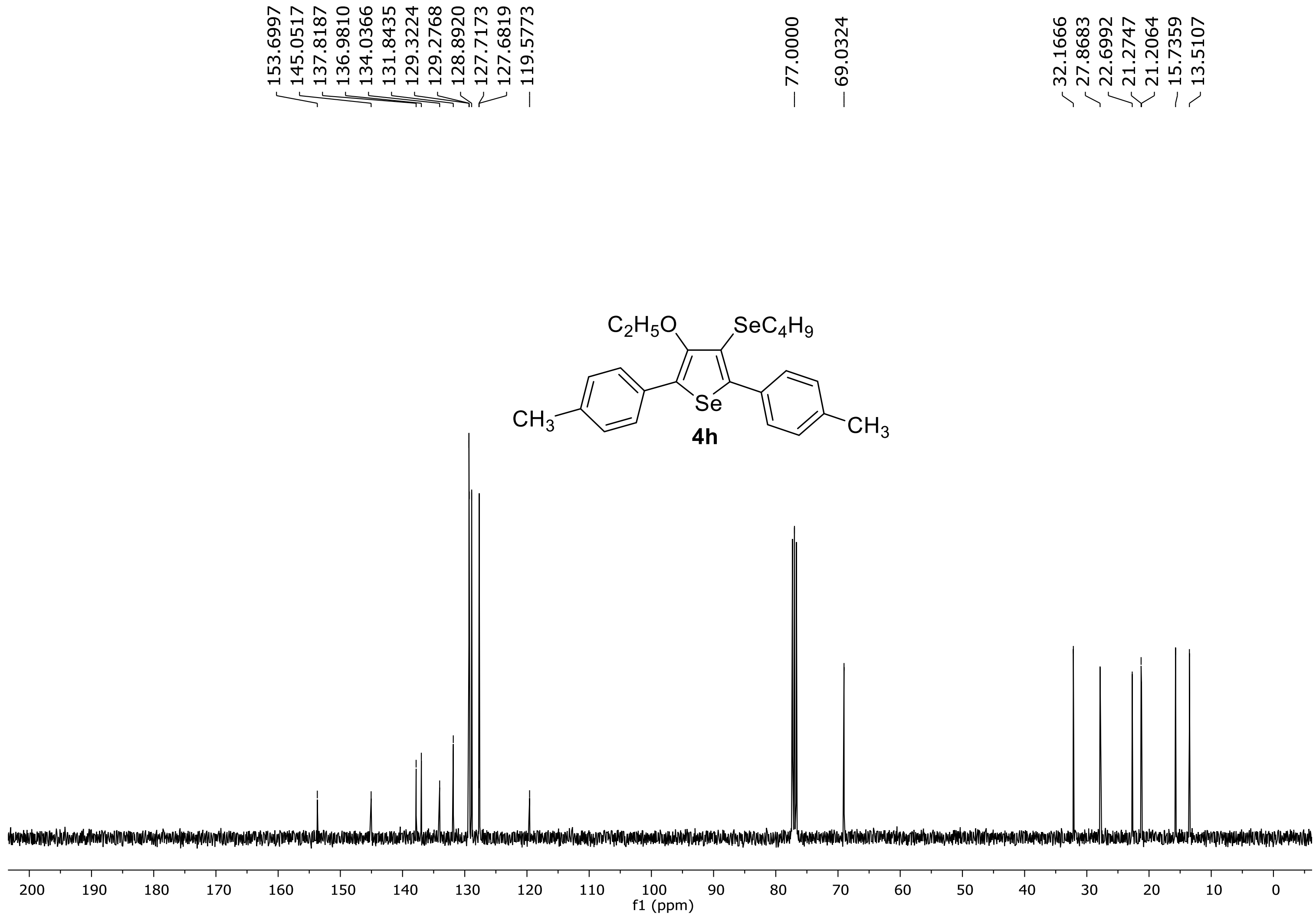

Figure S25: ^{13}C NMR (100 MHz, $CDCl_3$) spectrum of compound **4h**.

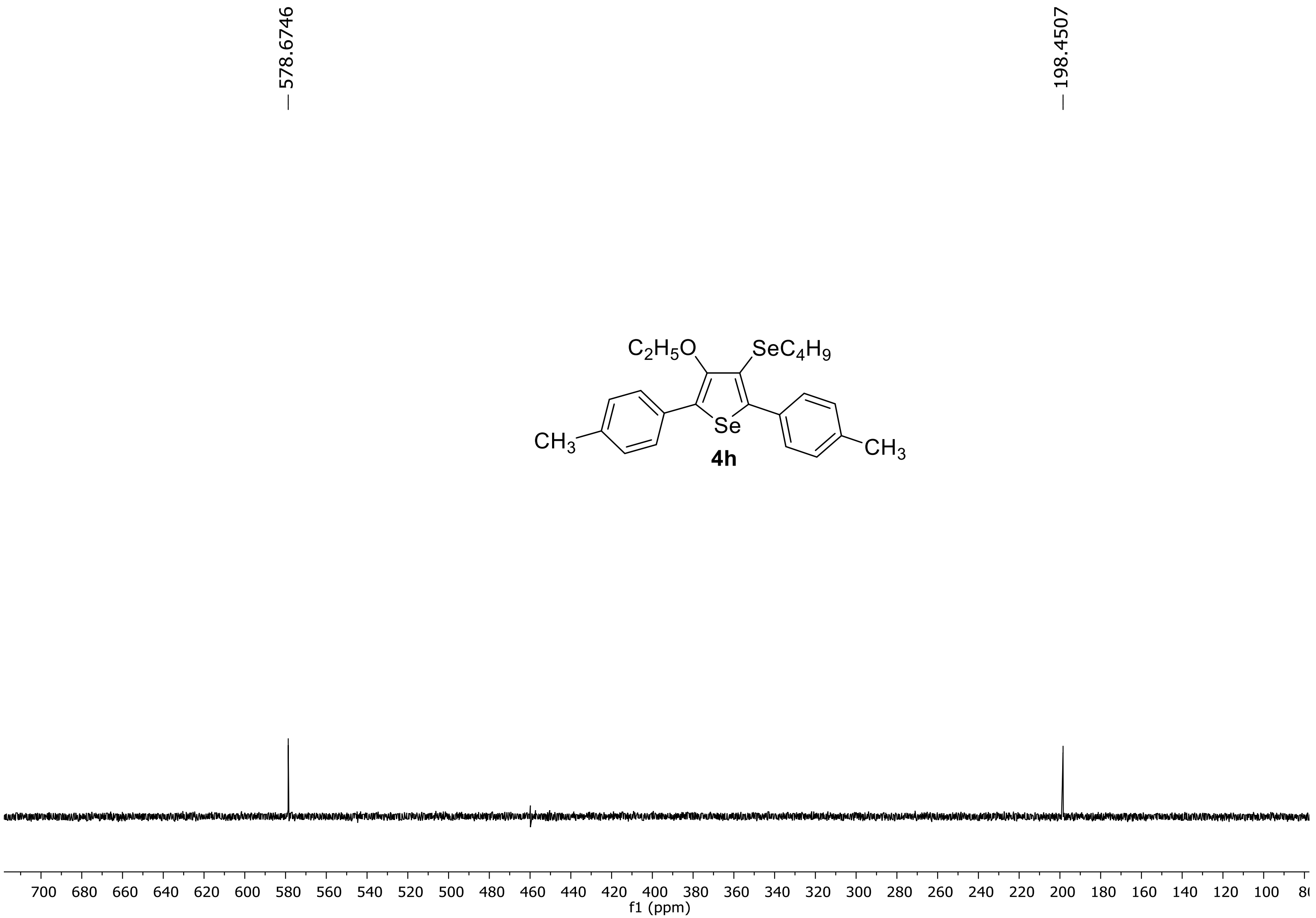

Figure S26: ^{77}Se NMR (76 MHz, $CDCl_3$) spectrum of compound **4h**.

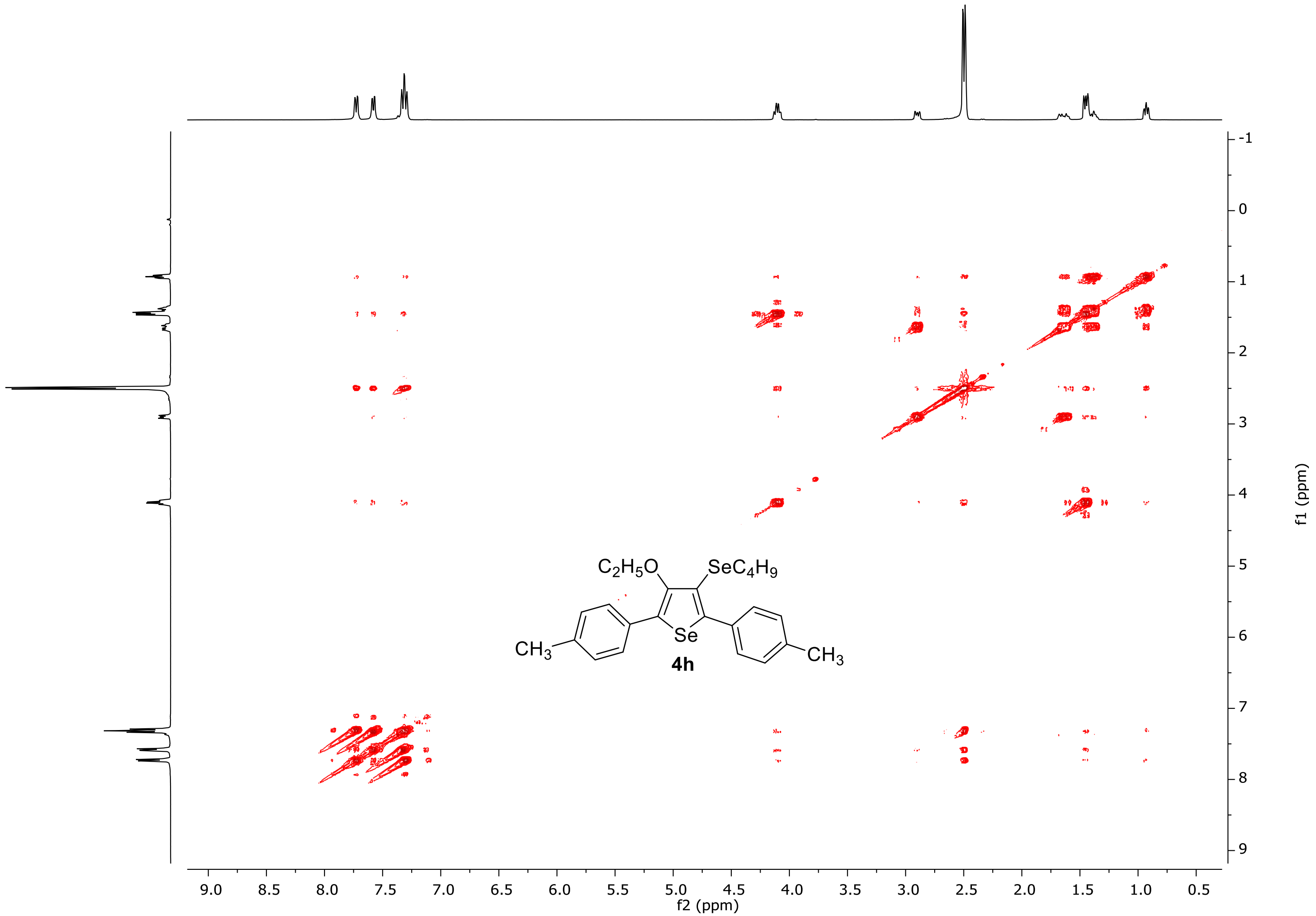

Figure S27: COSY NMR-2D (400 MHz, $CDCl_3$) spectrum of compound **4h**.

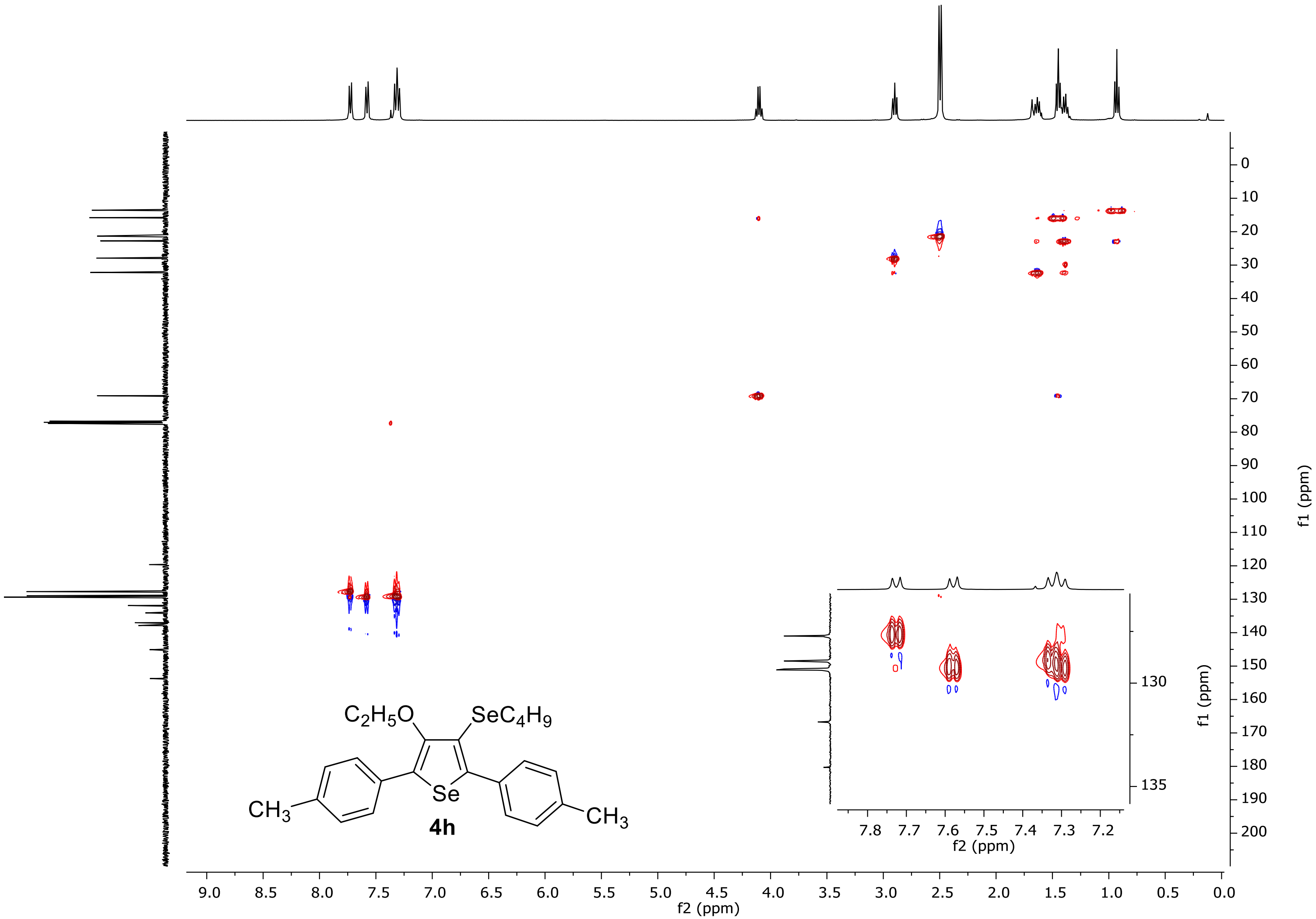

Figure S28: ^{1}H-^{13}C HSQC NMR-2D (400 MHz, $CDCl_3$) spectrum of compound **4h**.

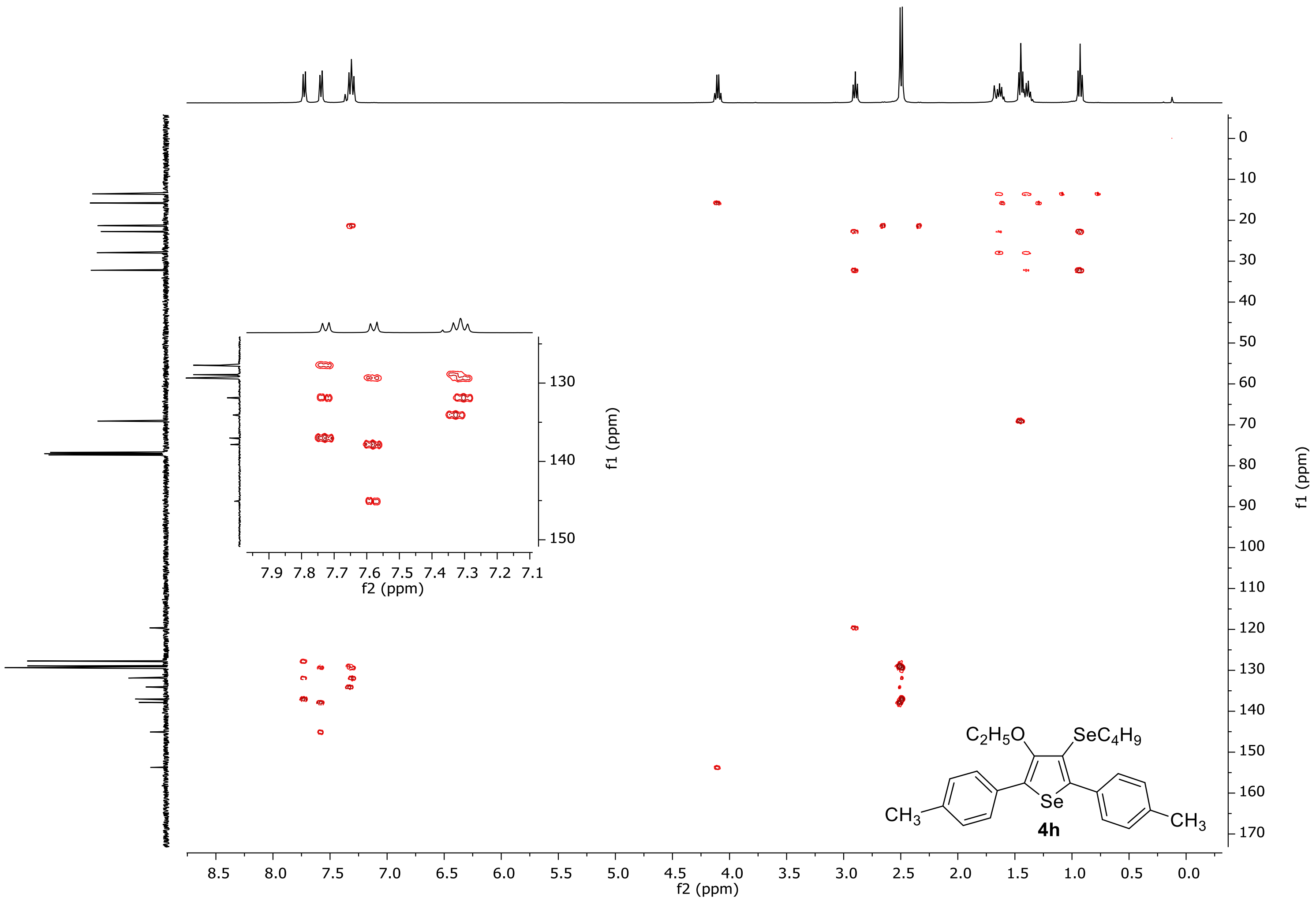

Figure S29: ^{1}H-^{13}C HMBC NMR-2D (400 MHz, $CDCl_3$) spectrum of compound **4h**.

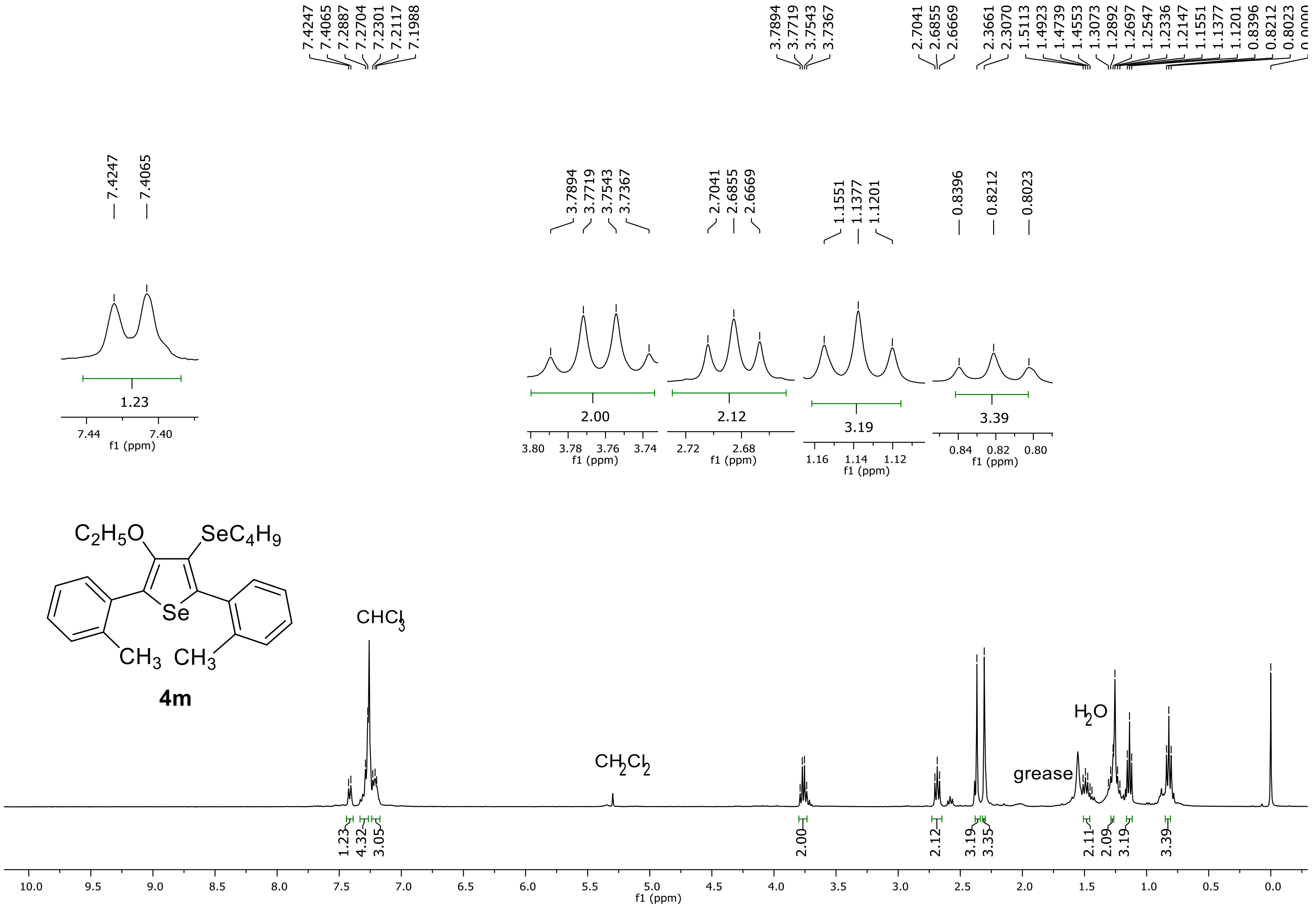

Figure S30: ^{1}H NMR (400 MHz, $CDCl_3$) spectrum of compound **4m**.

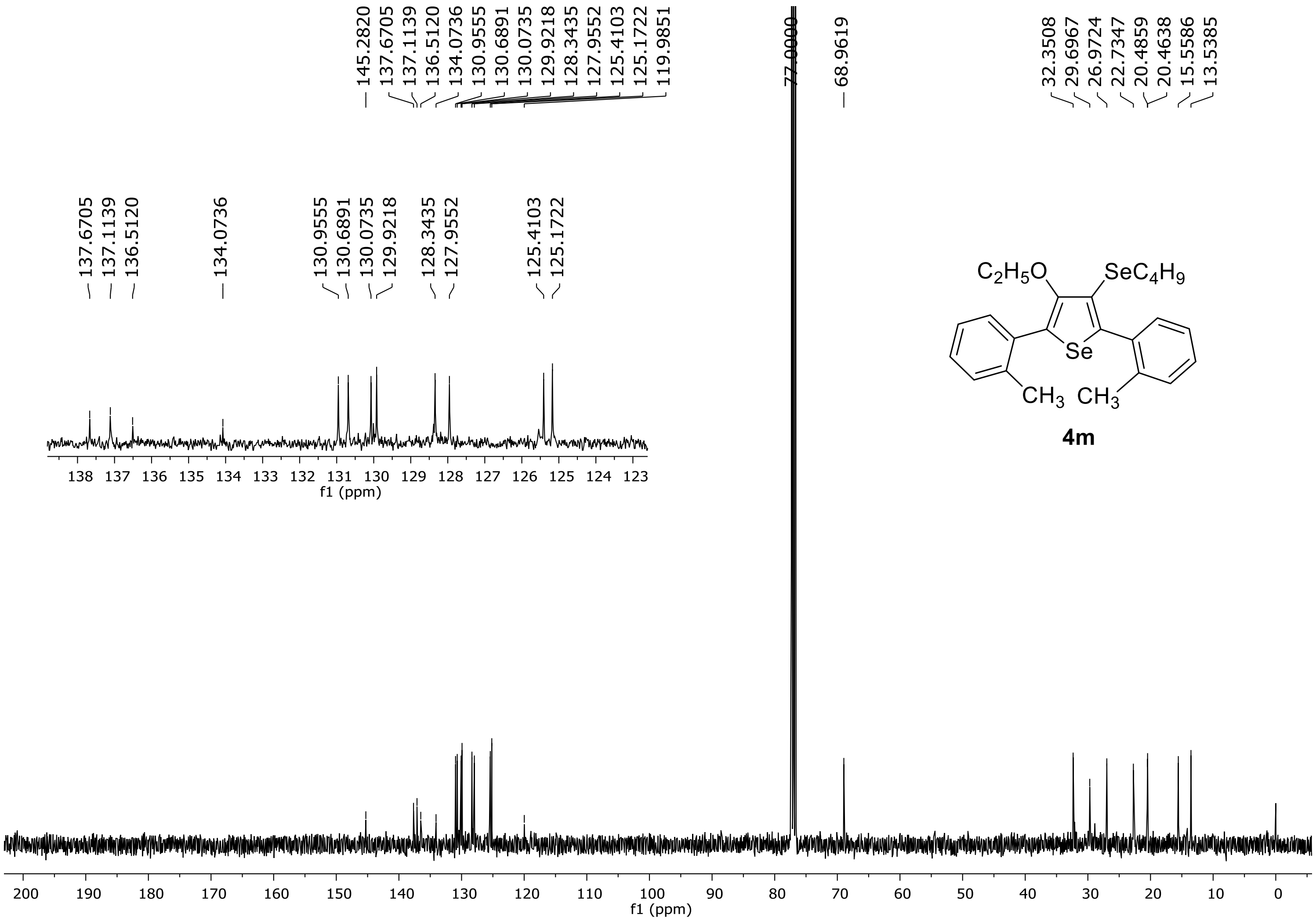

Figure S31: ^{13}C NMR (100 MHz, $CDCl_3$) spectrum of compound **4m**.

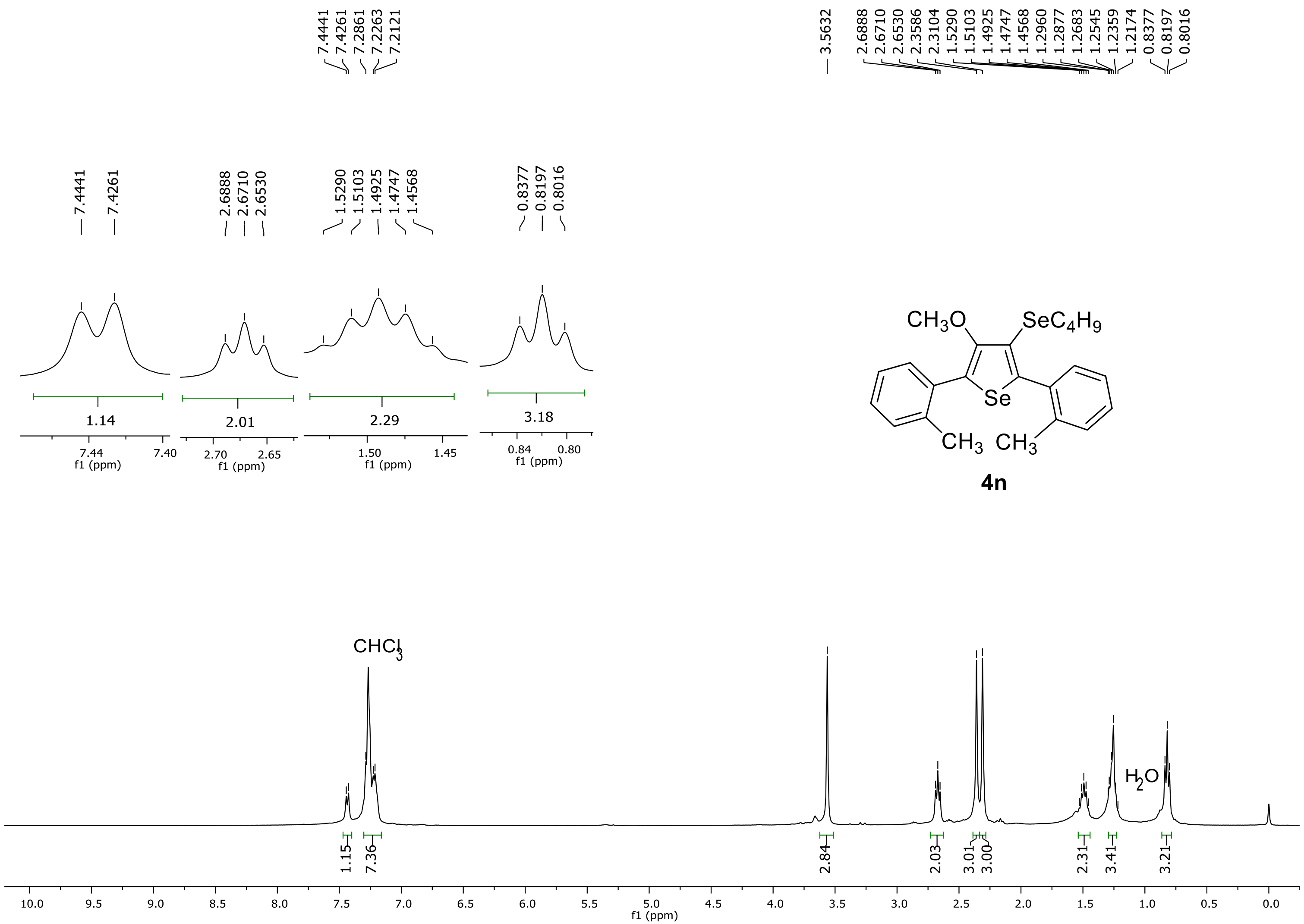

Figure S32: ^{1}H NMR (400 MHz, CDCl$_3$) spectrum of compound **4n**.

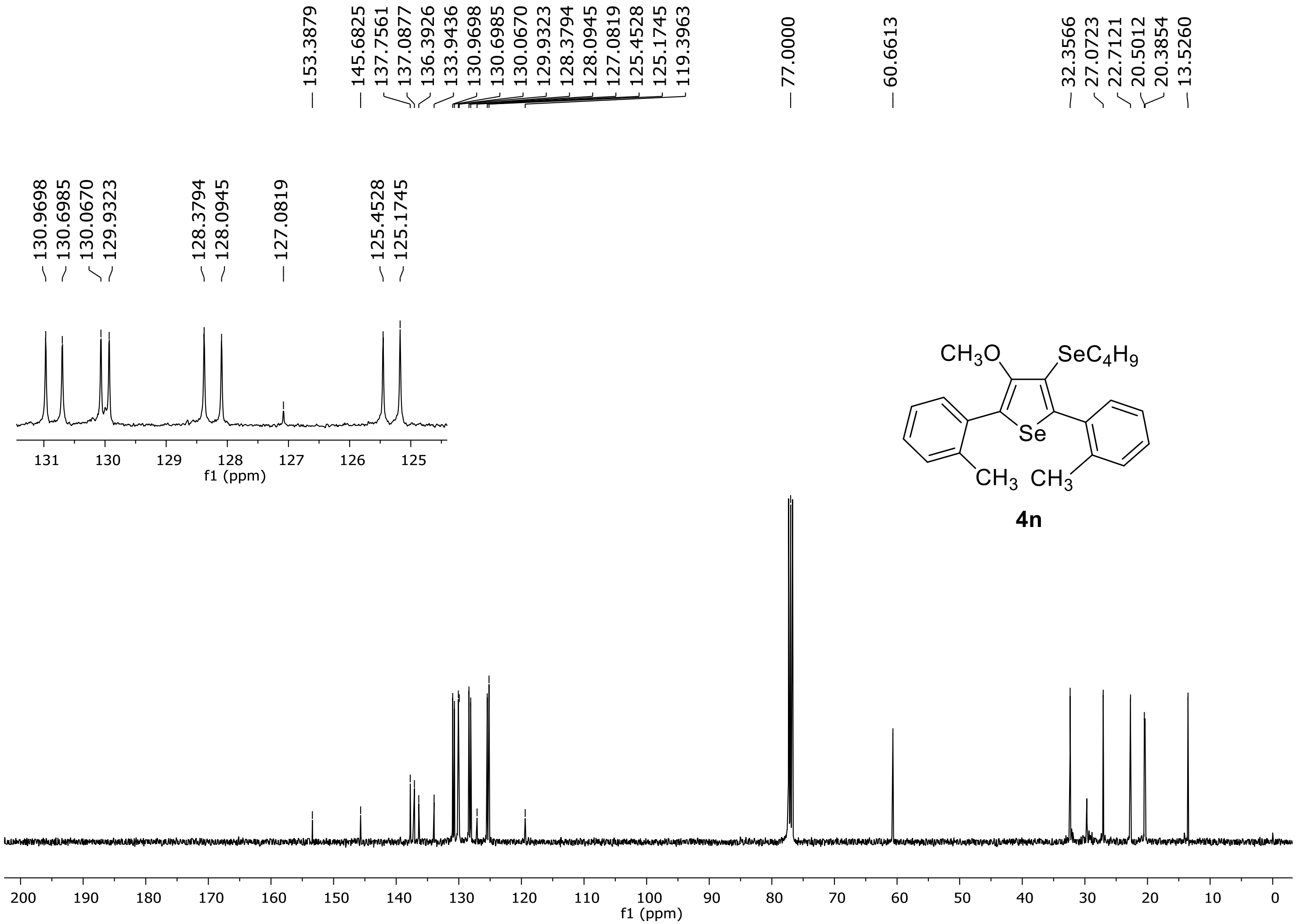

Figure S33: ^{13}C NMR (100 MHz, $CDCl_3$) spectrum of compound **4n**.

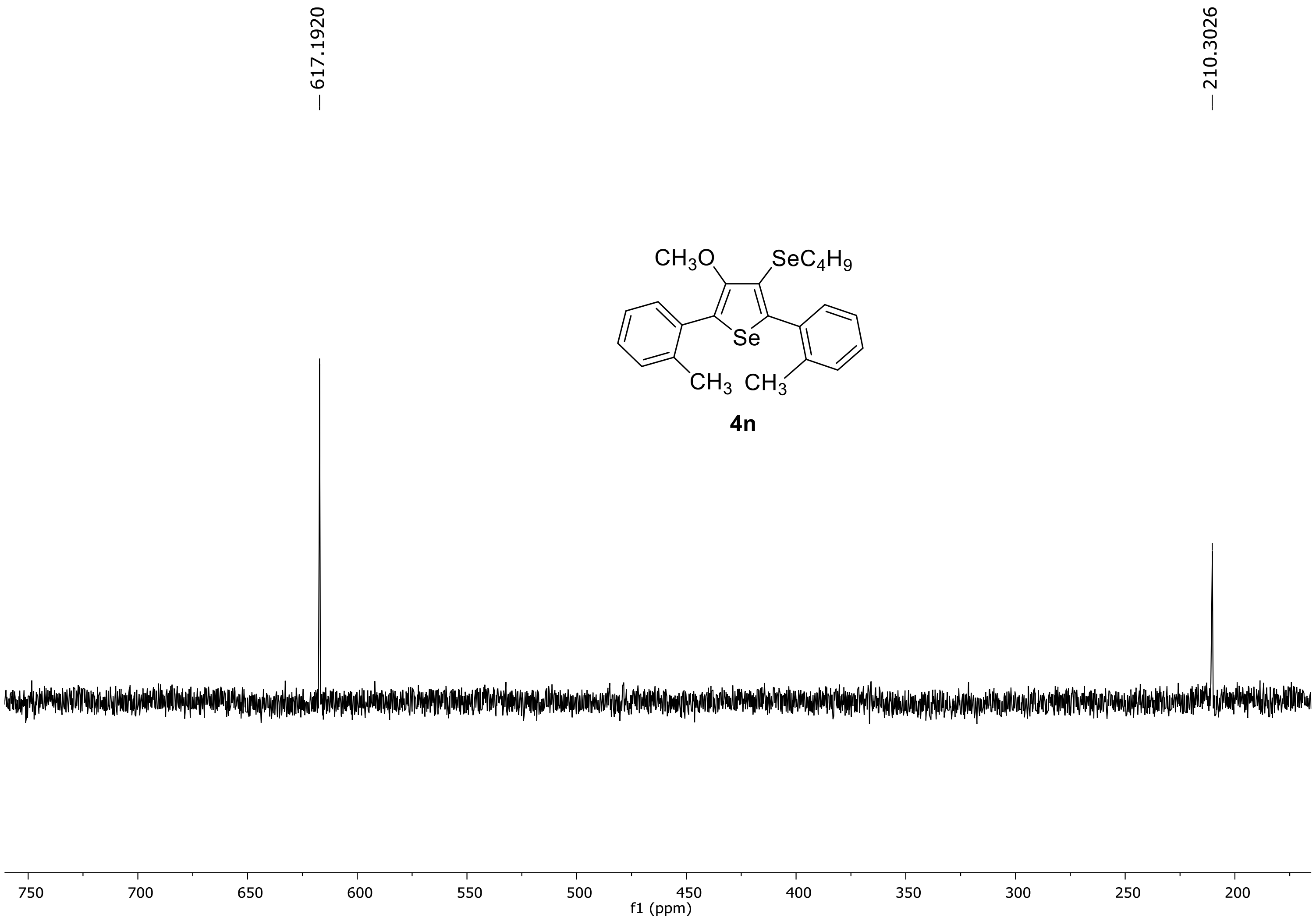

Figure S34: ^{77}Se NMR (76 MHz, $CDCl_3$) spectrum of compound **4n**.

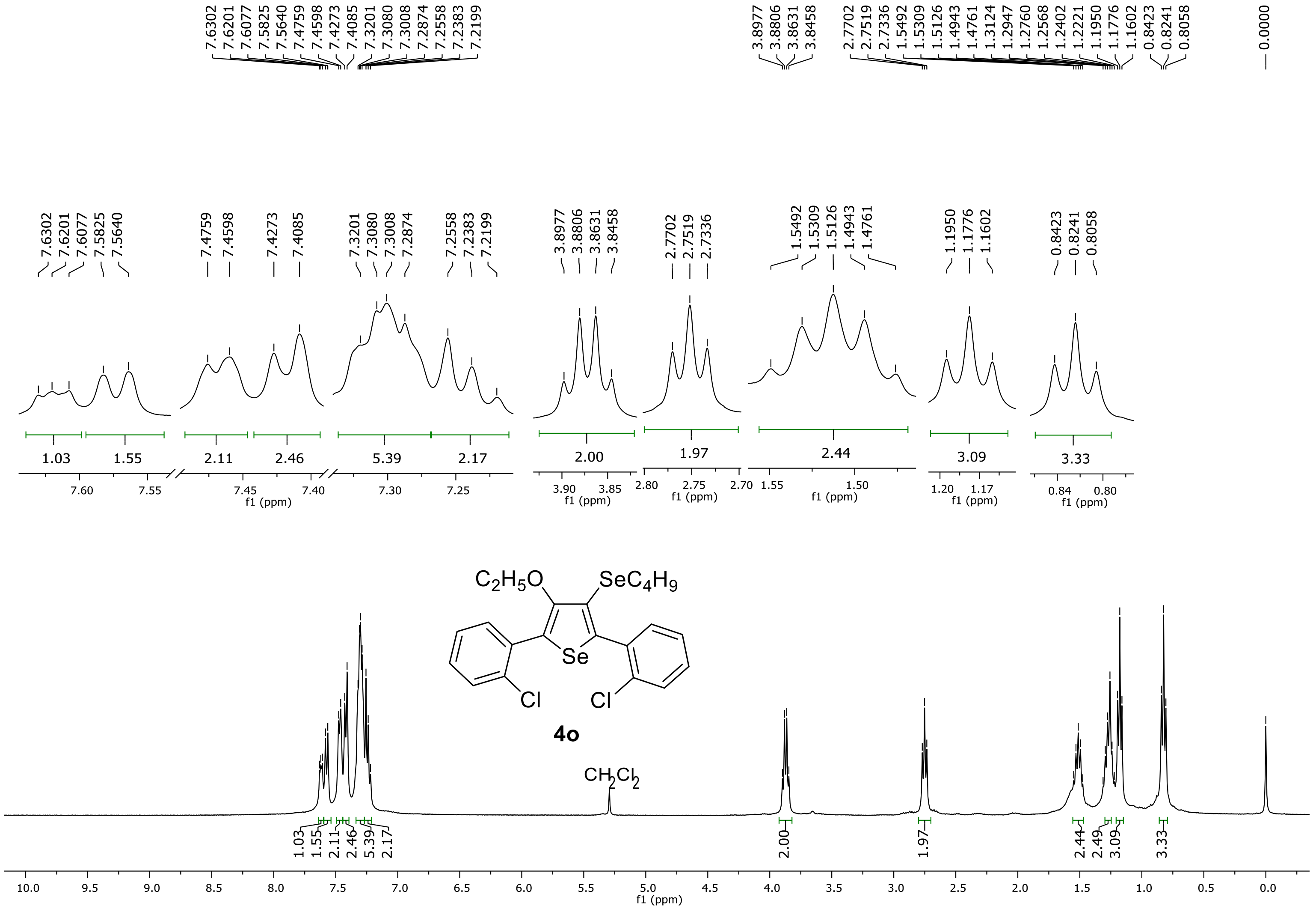

Figure S35: ^{1}H NMR (400 MHz, $CDCl_3$) spectrum of compound **4o**.

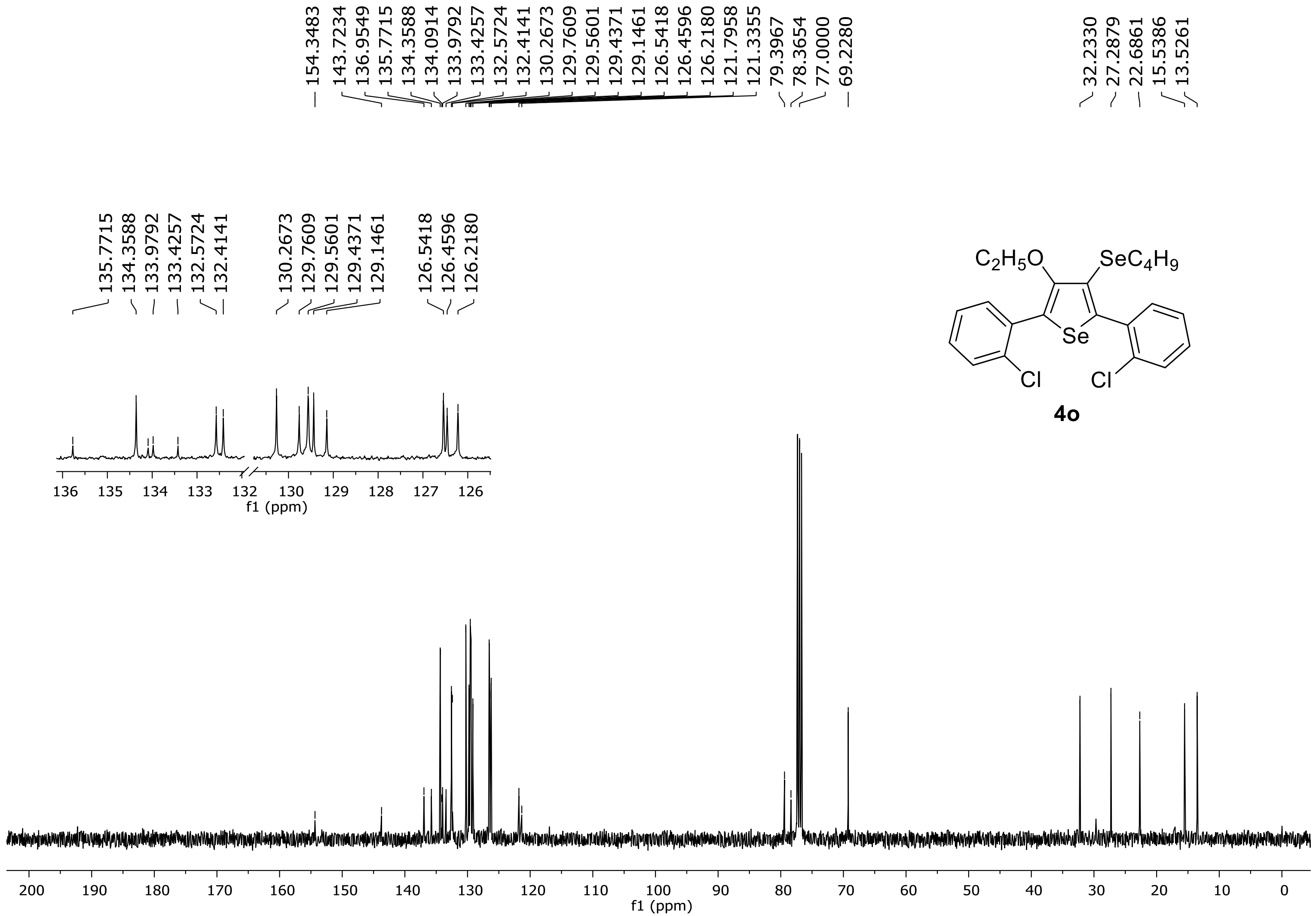

Figure S36: ^{13}C NMR (100 MHz, $CDCl_3$) spectrum of compound **4o**.

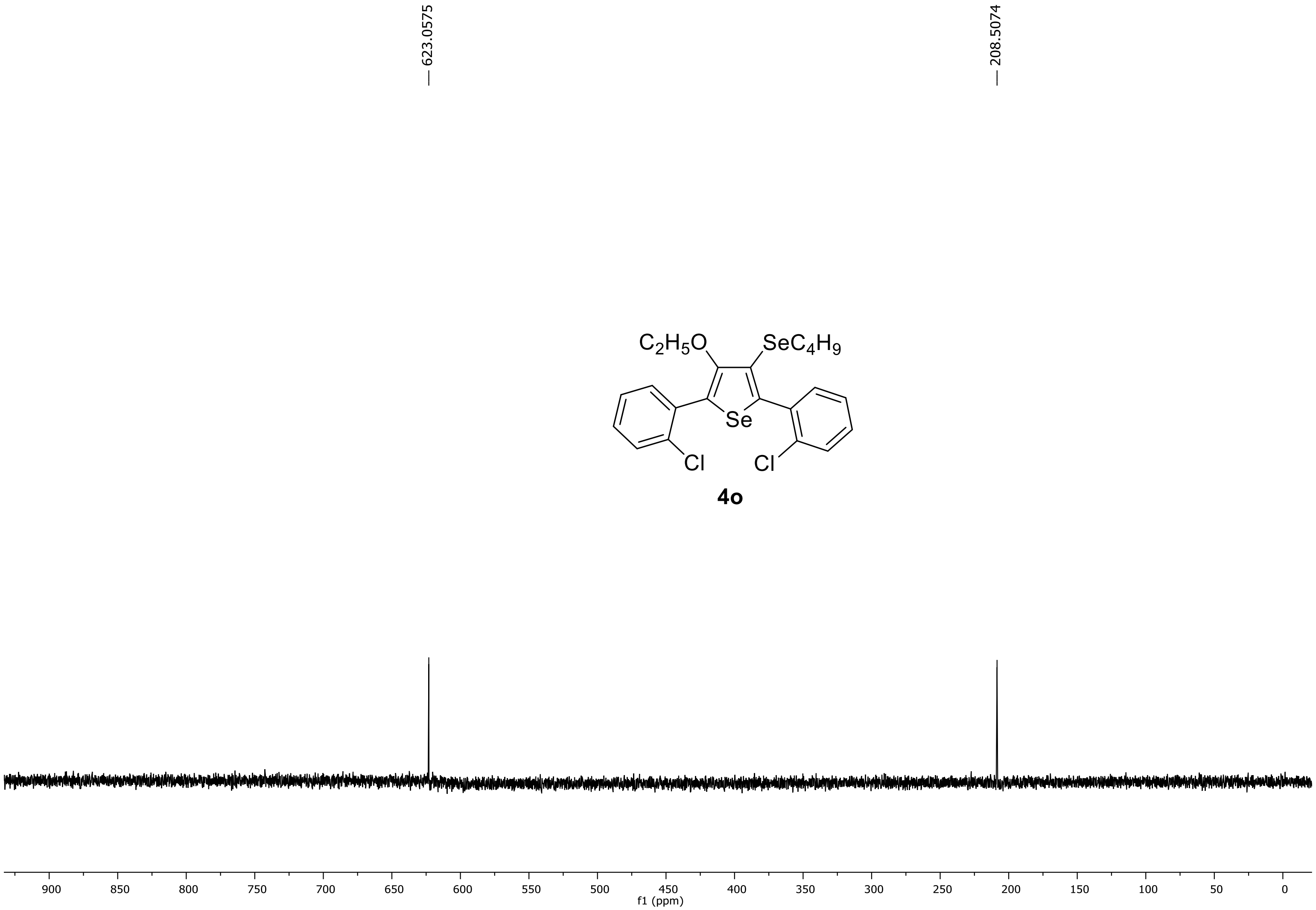

Figure S37: ^{77}Se NMR (76 MHz, $CDCl_3$) spectrum of compound **4o**.

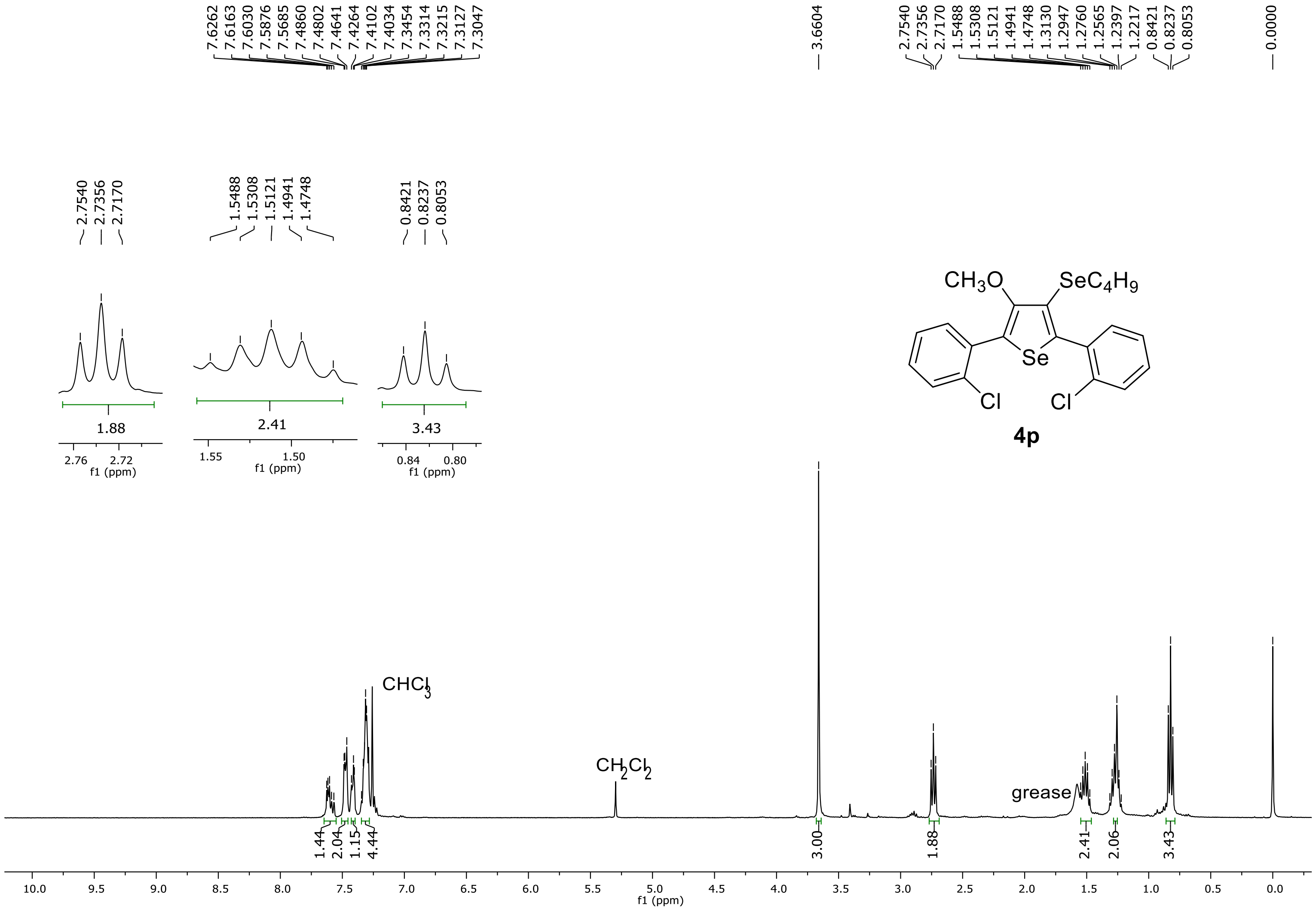

Figure S38: ^{1}H NMR (400 MHz, $CDCl_3$) spectrum of compound **4p**.

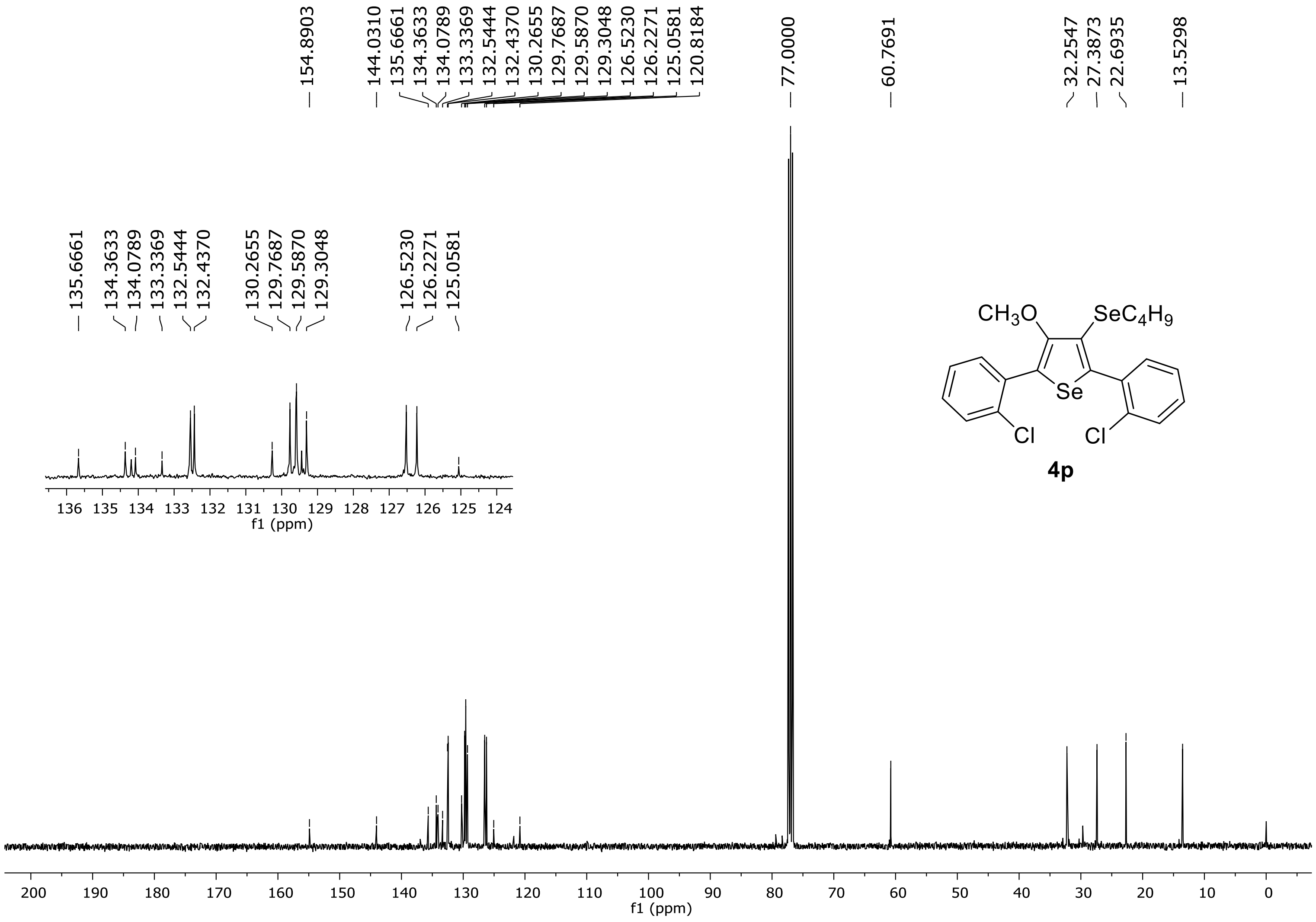

Figure S39: ^{13}C NMR (100 MHz, $CDCl_3$) spectrum of compound **4p**.

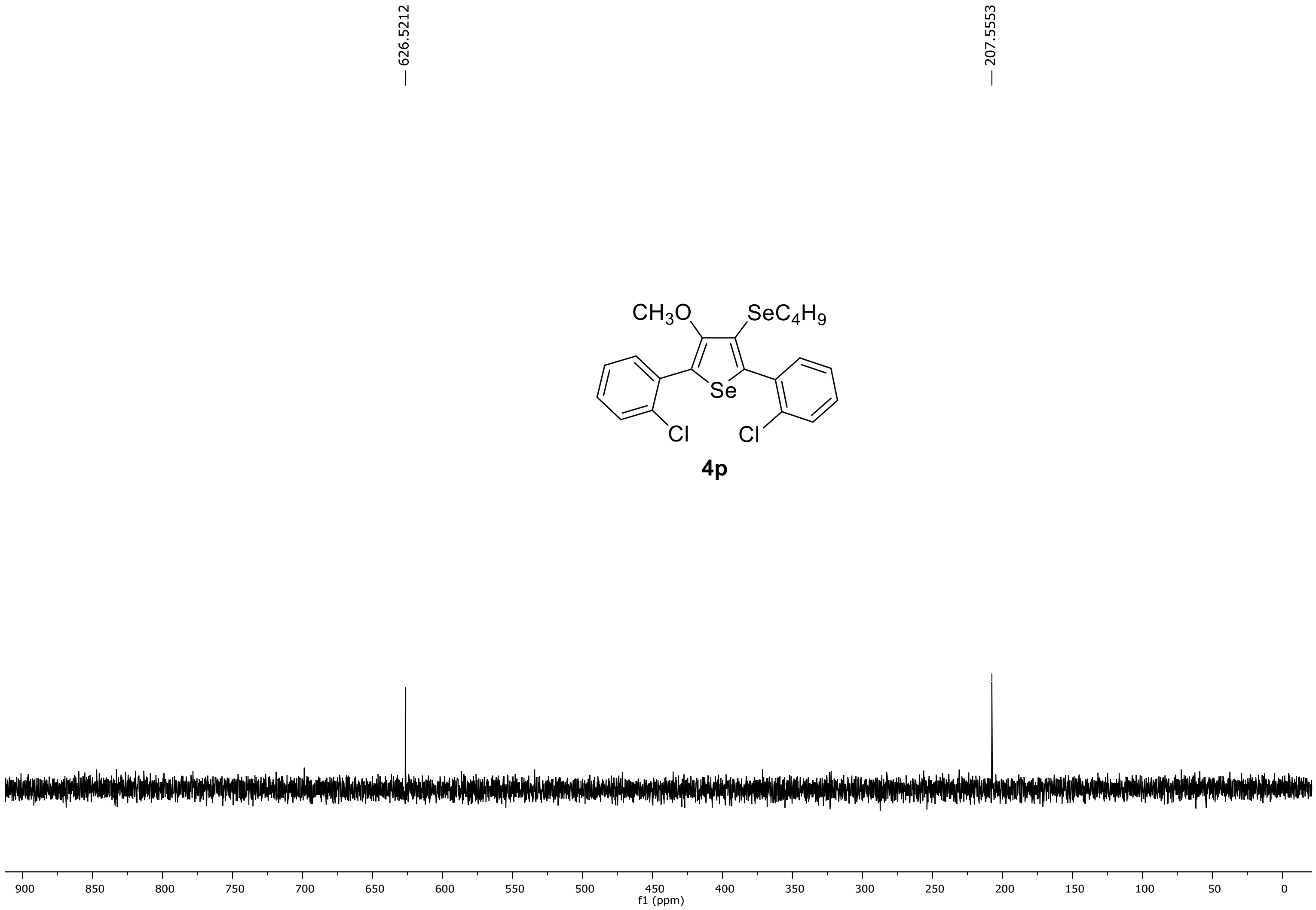

Figure S40: ^{77}Se NMR (76 MHz, $CDCl_3$) spectrum of compound **4p**.
